# Interactions on the Fermi Sphere in an All-Electron World: The Projector Augmented Wave Implementation of the Mott–Jones Hamilton Population and the Three Archetypal Sphere Packings

**DOI:** 10.1021/acs.jpcc.6c02214

**Published:** 2026-09-08

**Authors:** Leah C. Garman, Daniel C. Fredrickson

**Affiliations:** Department of Chemistry, 5228 University of Wisconsin-Madison, 1101 University Avenue, Madison, Wisconsin 53706, United States

## Abstract

The development of computational chemistry packages to translate the wavefunctions from a planewave DFT calculation to atom-centered functions has greatly contributed to the elucidation of bonding and stability in solid-state compounds. Fewer tools are available for the analysis of how nonlocalized, nearly-free electron character can be associated with stability, as proposed by the Mott–Jones (MJ) model. To bridge this divide, we developed the Mott–Jones Hamilton Population (MJHP) as a framework to extract the energetic contributions from interacting planewave pairs in a system’s electronic structure. This method illustrates how the MJ effects can be tied to the overall energy of a system and the origins of an electronic pseudogap for a variety of intermetallic phases. However, its implementation has so far been limited to DFT calculations with norm-conserving pseudopotentials. In this article, we present the Projector Augmented Wave realization of the MJHP (PAW-MJHP), aiming to bypass the limitations, computational costs, and high barrier of use associated with the norm-conserving pseudopotentials. We begin by extending the original MJHP equations to the PAW-DFT method and testing its application with the PAW-MJHP analysis of elemental Zn. This test case illustrates the influence of the PAW atomic dataset on the MJHP results, where softer pseudo-wavefunctions lead to unintuitive splittings in the band energy from the MJ effect with destabilizing interactions occurring lower in energy than stabilizing interactions. Based on these results, we propose a series of guiding principles for the development of new atomic datasets optimized for use with the MJHP. After establishing this framework, we apply the PAW-MJHP analysis and optimized atomic datasets to three example systems: CsCl-type CuZn and AgCd, and AuCu_3_-type AlNi_3_. In each case, the PAW-MJHP analysis confirms the MJ origins of electronic stability. In the process, we demonstrate the role that MJ interactions can play in structures based on each of the three archetypal simple packings: body-centered cubic (CuZn and AgCd), face-centered cubic (AlNi_3_), and hexagonal close-packed (Zn).

## Graphical abstract

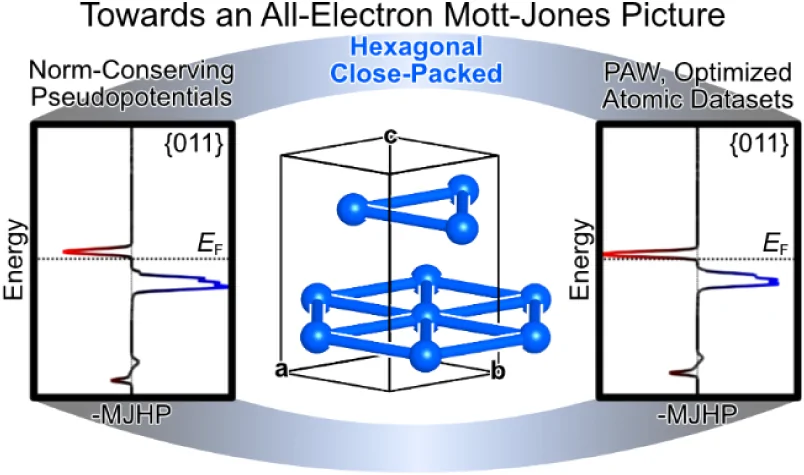

## 1 Introduction

Material design in intermetallics is enhanced by the availability of tools for understanding the chemical factors that underlie stability in terms of simple models. For example, the Crystal Orbital Hamilton Population reveals bonding and antibonding interactions between neighboring atoms,^1,2^ the DFT Chemical Pressure method investigates the space requirements of atoms in a structure,^3,4^ the Electron Localization Function and the Electron Localizability Indicator visualize electron pair probabilities to reveal chemical bonds,^5,6^ and the Bader charge analysis obtains atomic charges from zero-flux surfaces in charge density maps.^7,8^ Still needed, however, are tools for modeling the often nonlocalized character of the frontier orbitals that can be crucial for understanding the stability, properties, and reactivity of intermetallic phases.^9–11^ In particular, it remains challenging to determine definitively the mechanisms stabilizing Hume-Rothery phases, a family of intermetallic compounds with highly delocalized valence electrons.

The electronic stability of these phases, as evidenced by a pseudogap at the Fermi energy (*E*_F_), is often interpreted using the Mott–Jones (MJ) model.^12–16^ In this scheme, one begins by considering a metal as a homogeneous electron gas stabilized by a cationic background charge, with the electrons occupying free-electron planewave states within a sphere of reciprocal space centered around the origin, known as the Fermi sphere. The diameter of this sphere reflects the system’s valence electron concentration, the average number of valence electrons per atom ratio, in that it encloses the number of free-electron orbitals needed to accommodate all of the electrons present. Once the Fermi sphere is constructed, we imagine the effect of concentrating the background positive charge into an array of ion cores that weakly perturbs these states. When states on opposite sides of the Fermi sphere (with opposite wavevectors) interact in this situation, they can couple to produce a splitting in energy between their otherwise degenerate electronic levels. When the system is able to fill the lower energy states while leaving the higher energy states unoccupied, it is stabilized with respect to the free-electron system.

An alluring aspect of the MJ effect, from the viewpoint of experimental solid-state chemistry, is that the strength of these interactions can be qualitatively read from an X-ray diffraction pattern.^14^ The most intense diffraction peaks correspond to the reciprocal lattice vectors, {**G**_*hkl*_}, strongly coupling planewave electron states with **k** and **k′** such that **k**–**k′** = **G**_*hkl*_, especially where |**k**| = |**k′**| = *k*_F_, the radius of the Fermi sphere. This condition yields a prediction of the preferred valence electron concentration.^14^

The practical application of the MJ model for Hume-Rothery phases,^14,17–32^ polar intermetallics,^33–35^ and quasicrystal approximants^33,36–56^ has primarily relied on two factors: (1) the coincidence of the magnitude of the principal X-ray diffraction vectors with the diameter of the hypothetical free-electron Fermi sphere, and (2) the presence of a pseudogap at the *E*_F_ in the density of states (DOS) distribution. The assumption is that the pseudogap forms from the coupling of electron states on the Fermi sphere by these strongly diffracting reciprocal lattice vectors. However, the satisfaction of these conditions does not establish a causal relationship between them but is only suggestive of the presence of a MJ effect. Another challenge can be ambiguity in the effective valence electron concentration of a compound’s nearly-free electron orbitals. For example, it is straightforward to assign a Cu atom one valence electron, coming from the *4s* ^1^ configuration, but it is more difficult to assign a nearly-free valence electron count to atoms with open *d*-shells, such as Fe.^21^

For these reasons, we recently developed the Mott–Jones Hamilton Population as a theoretical framework to connect the MJ effect to the overall energy and electronic structure of a system.^57^ The MJHP extracts the Hamilton Populations for specific pairs of planewaves in the electronic structure, allowing contributions to the band energy from the MJ effects to be detected and quantitatively assessed. The MJHP results can be used to provide both (1) a comprehensive view of a system’s potential for MJ effects, identifying key reciprocal lattice vectors, and (2) a focused assessment for individual families of reciprocal lattice vectors. The application of these tools confirmed the role of MJ effects in a series of examples, including CuZn (β-brass, CsCl-type), CuZn_3_ (δ-brass, ZrTiP_0.68_-type), Cu_5_Zn_8_ (γ-brass, own type), Al_4_Cu_9_ (own type), Li_21_Si_5_ (Cu_41_Sn_11_-type), AlCu_3_ (BiF_3_-type), the quasicrystal approximant Zn_11_Au_15_Cd_23_, and Mg_17_Al_12_ (α-Mn-type).^57^

Despite these successes, further development of the MJHP is needed for its widespread application. Its implementation so far is based on the Hartwigsen–Goedecker–Hutter (HGH) norm-conserving pseudopotentials.^58^ While these pseudopotentials offer simplicity and transparency, several challenges arise from their use. The first difficulty is a question of the dependence of the MJHP results on the form of the HGH pseudopotentials. Without testing the method against other models for atoms, questions remain about the transferability of the results. Second, a large planewave basis set is often required to accurately model the hard pseudo-wavefunctions generated by these potentials. These large basis sets are computationally expensive, requiring long computational times and high memory capacity to adequately converge the numerical results. This is particularly cumbersome for calculations involving first-row transition metals, whose 3*d* orbitals have sharp features near their core regions, and some of these atoms figure prominently in Hume-Rothery compounds (e.g., Cu or Zn). Finally, the norm-conserving pseudopotential calculations have largely been supplanted by the Projector Augmented Wave (PAW) method, which offers greater computational efficiency and a clearer foundation in the all-electron wavefunctions.^59^

In this article, we introduce a PAW implementation of the MJHP, referred to as the PAW-MJHP, to address these challenges. The PAW method is based on a linear transformation that maps the pseudo-wavefunctions onto the all-electron wavefunctions, enabling the core–electron information to be retained (within the PAW atomic datasets, which take the role of pseudopotentials in the norm-conserving method) while converging the numerical results with a basis set size similar to that needed for an ultrasoft pseudopotential calculation.^59–61^ It has distinct conceptual advantages over those of pseudopotential methods: The pseudo-wavefunctions are directly related to their all-electron counterparts. In addition, the PAW atomic datasets are easily varied through using any of numerous open-source software packages,^62–64^ allowing the influence of the atomic datasets on the MJHP results to be established. Also, the softening of the pseudo-wavefunctions in PAW leads to decreased basis set sizes and improved computational efficiency. Finally, the PAW method is arguably the most popular method for performing electronic structure calculations for solid-state compounds, ensuring a lower barrier to use.

Here, we begin with the derivation of the PAW implementation of the MJHP and then test the PAW-MJHP analysis with different PAW atomic datasets, using elemental Zn as a demonstration case. Over the course of these tests, guiding principles will emerge for the optimization of atomic datasets for the MJHP analysis. Finally, we compare the norm-conserving pseudopotential and PAW-MJHP results across three example systems: CsCl-type CuZn^65^ and AgCd,^66^ and AuCu_3_-type AlNi_3_.^67^ When combined with our results for elemental Zn, these case studies will demonstrate the potential for MJ interactions to emerge in structures based on all three of the classic sphere packings: the body-centered cubic, face-centered cubic, and hexagonal close-packed arrangements.

## 2 Experimental Section

### 2.1 Electronic Structure Calculations

The nonspin-polarized ground-state electronic structures of elemental Al, Ni, Cu, Zn, Ga, Pd, Ag, Cd, Pt, and Au, and the binary compounds CuZn,^68^ AgCd,^69^ and AlNi_3_ ^70^ were calculated with the ABINIT^71–73^ package using density functional theory (DFT) with the Perdew–Burke–Ernzerhof (PBE)^74^ parametrization of the generalized gradient approximation and the projector augmented wave (PAW) method.^59–61^ Two sets of PAW-DFT calculations were done: one using the standard Jollet–Torrent–Holzwarth (JTH) atomic datasets,^75^ referred to as the original atomic datasets, with the second using atomic datasets optimized for the MJHP analysis when necessary (as described in Section 2.3). For comparison, electronic structure calculations were also performed using the local density approximation (LDA) and Hartwigsen–Goedecker–Hutter norm-conserving pseudopotentials (HGH-NCPPs).^58^

In each case, the crystal structures were optimized with an initial relaxation of the atomic positions within a fixed cell, followed by the simultaneous release of atomic positions and cell parameters. At each step of the algorithm, the electronic self-consistent field (SCF) cycle was iterated until convergence of the residuals of the potential between two sequential steps differed by less than 1.0 × 10^–10^. The geometry optimizations were performed on a *k*-point mesh that converged the total energy to <3 meV/atom. In preparation for the MJHP analysis and the calculation of the density of states (DOS) distributions, static calculations were performed using the optimized geometries, employing a *k*-point mesh at least two times as dense as the original mesh to ensure a high-resolution sampling of the frontier orbitals. The DOS distributions were calculated on these fine *k*-point grids using the tetrahedron method.^76^ Detailed computational parameters, including the *k*-point meshes, energy cutoffs, and optimized geometries, may be found in the Supporting Information.

### 2.2 The PAW-MJHP Analysis

The MJ effects in each system were investigated with the MJHP analysis using the pawMJHP_analysis^77^ and MJHP_analysis^57^ programs for the PAW and HGH-NCPP DFT calculations, respectively. For the *MJHP*(*E*, 2θ) analysis, the ground-state density from the single-point energy calculations was read for the simulation of the powder X-ray diffraction patterns. The reciprocal lattice vectors with non-negligible diffraction intensity were folded back into the Brillouin zone, and a non-self-consistent calculation was performed with ABINIT on the resulting set of high-symmetry *k*-points using the SCF density as input. The local effective potential, pseudo-wavefunctions, and atomic datasets were then processed according to the analysis described in Section 3.2 for the PAW calculations and, previously, for the HGH-NCPP DFT calculations.^57^

In the *MJHP_HKL_* (*E*) analysis, output from the ground-state SCF calculations is read and processed following the routine described in Section 3.2.

### 2.3 Atomic Dataset Generation

The MJHP-optimized PAW atomic datasets for Al, Ni, Zn, Ga, Ag, Cd, Pt, and Au were created using the *atompaw* software package.^62^ The standard JTH atomic datasets were used as a starting point and then modified according to the process outlined in Section 4.^75^ Following these modifications, all other input parameters were systematically varied to produce the lowest differences in the equation of state calculated with the optimized atomic datasets and the WIEN2k^78^ all-electron reference results using the Δ software package.^79,80^

The electronic structures for the Δ calculations were obtained using the atomic positions and lattice parameters provided in the Δ software package without further optimizations. Single-point energy calculations for the Δ evaluation were carried out on the elemental crystal structures at seven volumes ranging from 0.94 *V*_*cell*_ to 1.06 *V*_*cell*_, where *V*_*cell*_ is the equilibrium unit cell volume from the geometry provided in the Δ software package. The static calculations were performed on a 32 × 32 × 32 *k*-point mesh for the hexagonal close-packed (hcp) systems (Zn and Cd) and Ga and a 40 × 40 × 40 *k*-point grid for the face-centered cubic (fcc) systems (Al, Ni, Ag, Pt, and Au). In all calculations, the kinetic energy cutoff that determines the size of the planewave basis set at a given *k*-point (*ecut*) and the energy cutoff for the fine mesh grid used to convert the spherical atom-centered grids onto the mesh grid (*pawecut*) were set to 40 Ha, the physical temperature broadening of the occupation numbers (*tsmear*) was set to 0.002 Ha, and the smearing of the kinetic energy cutoff (*ecutsm*) was set to 0.5 Ha. The volumes and total energies for the seven geometries were extracted and read into the Δ software package to determine the equilibrium volume, the bulk modulus, and the first derivative of the bulk modulus with respect to pressure. These parameters were used to evaluate the difference between the equation of state, the Δ-value, between the all-electron and PAW calculations (see the Supporting Information for a formal definition).

The mean Δ-value for the original atomic datasets for Al, Ni, Zn, Ga, Ag, Cd, Pt, and Au is 0.42 meV/atom,^75^ while the average Δ-value for the MJHP-optimized atomic datasets of the same elements is 0.66 meV/atom. Although the original atomic datasets have lower Δ-values, the MJHP-optimized atomic datasets are under the 0.95 meV/atom threshold to be considered in good agreement with the reference dataset.^81^ As a note, none of these materials have particularly small bulk moduli, so the Δ-value serves as a valid comparison metric.

The quality of the atomic datasets was also assessed by comparing the logarithmic derivatives of the all-electron and pseudo-radial wavefunctions with respect to *r*, which are output by the *atompaw* software during the atomic dataset generation. The decomposition of the band energies into contributions from reciprocal lattice vectors of different magnitudes was performed with the *box_the_band* script, which is part of the *pawMJHP_analysis* package.^77^ The finalized atomic datasets optimized for MJHP analysis are also available with this package.

## 3 The PAW Mott–Jones Hamilton Population

### 3.1 The Mott–Jones Hamilton Population in the PAW Formalism

The Mott–Jones Hamilton Population (MJHP) provides a theoretical framework for directly connecting the Mott–Jones effect to a system’s overall energy and electronic structure. So far, though, its implementation has focused on the HGH norm-conserving pseudopotential method. We now extend the implementation of the MJHP to the Projector Augmented Wave (PAW) formalism, the current workhorse of electronic structure calculations on inorganic materials.

At the crux of the PAW method is a linear transformation that maps the all-electron wavefunctions that we aim to calculate, |ψ**_k_**_,__*n*_⟩, onto simpler pseudo-wavefunctions, |ψ̃**_k_**_,__*n*_⟩:

1 $$|\psi_{k,n}\rangle =|{\mathrm{\psi ̃}}_{k,n}\rangle +\underset{i}{\sum }\left(|\phi_{i}\rangle -|{\mathrm{\phi ̃}}_{i}\rangle \right)\langle {\mathrm{p̃}}_{i}\left|{\mathrm{\psi ̃}}_{k,n}\right\rangle$$

where ϕ_*i*_, ϕ̃_*i*_, and *p̃_i_* are the all-electron partial waves, the pseudo-partial waves, and the projector functions, respectively, and *i* runs over the different partial waves and projector functions for the atomic sites.^82^ This relationship highlights the fundamental advantages of the PAW formalism over pseudopotential methods: the pseudo-wavefunctions used within the SCF cycles in the DFT calculation directly correspond to all-electron wavefunctions, which can be constructed at any time.

Being smooth, the pseudo-wavefunctions are easily expressed as a linear combination of planewaves:

2 $${\mathrm{\psi ̃}}_{k,n}\left(r\right)=\frac{1}{\sqrt{V_{cell}}}\underset{hkl}{\sum }c_{hkl,k,n}e^{i(G_{hkl}+k)\cdot r}$$

where **G**_*hkl*_ is the reciprocal lattice vector *h* **a** ^*^ + *k* **b** ^*^ + *l* **c** ^*^, and *V*_*cell*_ is the unit cell volume. Modeling the pseudo-wavefunctions requires smaller planewave basis sets compared to those needed for all-electron wavefunctions or norm-conserving pseudopotential methods, leading to enhanced computational efficiency.

The conceptual and computational gains associated with the PAW method come at the cost of the complexity in its implementation. For instance, the PAW method uses two different grid types to sample the space. The first is a regular mesh grid that runs over all space, on which the pseudo-wavefunctions are defined. Within the augmentation spheres about each atomic site, however, radial grids are used for calculations related to partial waves and projector functions. As we will see, this distinction adds a layer of intricacy to the derivation of the MJHP, particularly for its effective-nonlocal potential energy terms.

A simple place to start in building the PAW implementation of the MJHP is with the system’s total band energy. It is found by adding the contributions from the kinetic energy (*E* ^*kinetic*^), the local effective potential energy (*E* ^*eff*‑*local*^, including the Hartree and exchange-correlation terms), and the nonlocal effective energy (*E* ^*eff*‑*nonlocal*^, which includes all terms involving the PAW projectors):

3 $$E^{band}=E^{kinetic}+E^{\mathrm{eff-local}}+E^{\mathrm{eff-nonlocal}}$$

The contribution of the MJHP to the kinetic energy is simple to assess. As each planewave contributes to the kinetic energy individually, it is not expected to participate in the MJ effect, which arises from interactions between different planewaves. As in the original MJHP, our attention will thus focus on only the potential energy terms.

The first of these terms is the effective-local potential energy contribution. The total effective-local potential energy can be resolved into a sum of contributions from pairs of planewaves:

4 $$E^{\mathrm{eff-local}}=\underset{k,n}{\sum }w_{k}o_{k,n}\underset{hkl}{\sum }\underset{h^{\prime }k^{\prime }l^{\prime }}{\sum }c_{hkl,k,n}^{*}c_{h^{\prime }k^{\prime }l^{\prime },k,n}{Ṽ}_{h-h^{\prime },k-k^{\prime },l-l^{\prime }}^{\mathrm{eff-local}}$$

where *w*_**k**_ gives the multiplicity of each *k*-point within the irreducible wedge of the reciprocal lattice, *o*_*n*,**k**_ gives the band occupations, and ${Ṽ}_{h-h^{\prime },k-k^{\prime },l-l^{\prime }}^{\mathrm{eff-local}}$ is the Fourier component of the pseudo-effective potential, *Ṽ*^*eff*‑*local*^(**r**) from **G**_*h*–*h*′,*k*–*k*′,*l*–*l*′_. In the context of the MJHP, we are interested in collecting only a narrow set of these planewave pairs that satisfy the Mott–Jones conditions: (1) their wavevectors, (**k** + **G**_*hkl*_) and (**k** + **G**_*h*′*k*′*l*′_), must be separated by the reciprocal lattice vector of interest, **G**_*HKL*_ = **G**_*h*–*h*′,*k*–*k*′,*l*–*l*′_ = (**k** + **G** *_hkl_*) – (**k** + **G**_*h*′*k*′*l*′_), and (2) the vectors should lie near the proposed free-electron Fermi sphere. To impose the latter condition, the wavevectors must reside within the reciprocal space shell defined by:

5 $$\frac{\left|G_{HKL}\right|}{2}-\Delta_{shell}\leq \left|k+G_{hkl}\right|\leq \frac{\left|G_{HKL}\right|}{2}+\Delta_{shell}$$

where, Δ_*shell*_ is an adjustable parameter (set to 0.075 Å^–1^ for all systems; see the Supporting Information for a look at the dependence of the results on this parameter) that defines the thickness of the shell.

To filter only those contributions from planewave pairs that meet these criteria, we apply to the terms in eq 4 a weighting function of the form:

$$S_{HKL}\left(k,hkl,h^{\prime }k^{\prime }l^{\prime }\right)=\delta_{h-h^{\prime },H}\delta_{k-k^{\prime },K}\delta_{l-l^{\prime },L}e^{-{\left(|k+G_{hkl}|-|k+G_{h^{\prime }k^{\prime }l^{\prime }}|\right)}^{2}/\sigma }$$ 6

Here, δ_*h*–*h*′,_ *_H_* δ_*k*–*k*′,_ *_K_* δ_*l*–*l*′,*L*_ ensures the potential energy contribution from a pair of planewaves is zero unless *h*–*h*′ = *H*, *k*–*k*′ = *K*, and *l*–*l*′ = *L*. The Gaussian function is included to give full weight to those pairs of planewaves that reside on the faces of the Jones Zone and to gradually fade the contributions in proportion to the distance from this face. The σ parameter controls how sharply the contributions are extinguished (set to 0.1 Å^–2^ for all calculations performed here; see the Supporting Information for further details). Using this weighting function, we arrive at the effective-local potential energy contribution to the MJHP:

7 $$MJHP_{HKL,k,n}^{\mathrm{eff-local}}=\underset{hkl}{\sum }\underset{h^{\prime }k^{\prime }l^{\prime }}{\sum }S_{HKL}\left(k,hkl,h^{\prime }k^{\prime }l^{\prime }\right)w_{k}c_{hkl,k,n}^{*}c_{h^{\prime }k^{\prime }l^{\prime },k,n}{Ṽ}_{HKL}^{eff,local}$$

So far, the implementation of the MJHP is the same regardless of whether we consider the norm-conserving pseudopotential or the PAW method. The effective-nonlocal energy, however, depends intrinsically on the PAW formalism. The total effective-nonlocal energy is found by calculating the expectation values of the nonlocal operator derived from the projector functions with the pseudo-wavefunctions:

8 $$E^{\mathrm{eff-nonlocal}}=\underset{k,n}{\sum }w_{k}o_{k,n}\underset{ij}{\sum }{\langle \mathrm{\Psi ̃}}_{k,n}|{\mathrm{p̃}}_{i}{\rangle D}_{ij}{\langle \mathrm{p̃}}_{j}|{\mathrm{\Psi ̃}}_{k,n}\rangle$$

The nonlocal matrix element, *D*_*ij*_, is the combination of three independent terms:

9 $$D_{ij}=D_{ij}^{1}-{\mathrm{D̃}}_{ij}^{1}+{Ď}_{ij}$$

$D_{ij}^{1}$ and ${\mathrm{D̃}}_{ij}^{1}$ are the effective potentials from the all-electron and pseudo-partial waves, respectively, while *Ď*_*ij*_ gives the effective potential from the compensation charge, which ensures the Coulomb potentials in one on-site term have no contributions to neighboring spheres.^60,83^ When using ABINIT, the nonlocal matrix element, *D*_*ij*_, is output for each atomic site at the end of the PAW DFT calculation. The effective-nonlocal energy can be simply expressed as a sum, over each *k*-point, band, atomic position, and *i*-index, of the element-wise products of the nonlocal matrix and a density matrix, ρ_*ij*_, whose elements encode the occupation and density for the on-site terms and are defined for each partial wave at one atomic site:

10 $$E^{\mathrm{eff-nonlocal}}=\underset{k,n}{\sum }w_{k}o_{k,n}\underset{ij}{\sum }\rho_{ij,k,n}D_{ij}$$

This density matrix can in turn be expressed as a sum of interactions between planewave pairs by expanding the pseudo-wavefunctions on a real spherical harmonic basis set:

$$\rho_{ij,k,n}=\left\langle {\mathrm{\Psi ̃}}_{k,n}\right|{\mathrm{p̃}}_{i}\rangle \left\langle {\mathrm{p̃}}_{j}\right|{\mathrm{\Psi ̃}}_{k,n}\rangle =\frac{{\left(4\pi \right)}^{2}}{V_{cell}}\underset{hkl}{\sum }\underset{h^{\prime }k^{\prime }l^{\prime }}{\sum }c_{hkl,k,n}^{*}c_{h^{\prime }k^{\prime }l^{\prime },k,n}Y_{i}\left({Û}_{k+G_{hkl}}\right)Y_{j}\left({Û}_{k+G_{h^{\prime }k^{\prime }l^{\prime }}}\right)e^{i\left(G_{h^{\prime }k^{\prime }l^{\prime }}-G_{hkl}\right)\cdot R}{\overset{¯}{\mathrm{p̃}}}_{i}\left(\left|k+G_{hkl}\right|\right){\overset{¯}{\mathrm{p̃}}}_{j}\left(\left|k+G_{h^{\prime }k^{\prime }l^{\prime }}\right|\right)$$ 11

where *Y*_*i*_(*Û*_**k** + **G** *_hkl_*_) is the real spherical harmonic for partial wave *i* evaluated along the direction of **k** + **G**_*hkl*_, **R** is the atomic position at the center of the partial waves *i* and *j*, and ${\overset{®}{\mathrm{p̃}}}_{i}\left(\left|k+G_{hkl}\right|\right)$ is the radial Fourier transform of the projector function, *p̃*_*i*_ (see the Supporting Information for a formal definition).

In an analogous manner to the effective-local potential, we aim to extract only those pairs of planewaves that satisfy the Mott–Jones conditions. We do so by embedding the weighting function, *S_HKL_* (**k**, *hkl*, *h*′*k*′*l*′), into the definition of the density matrix in eq 11 to define the MJHP-density matrix, $\rho_{HKL,ij,k,n}^{MJHP}$ :

$$\rho_{HKL,ij,k,n}^{MJHP}=\frac{{\left(4\pi \right)}^{2}}{V_{cell}}\underset{hkl}{\sum }\underset{h^{\prime }k^{\prime }l^{\prime }}{\sum }S_{HKL}\left(k,hkl,h^{\prime }k^{\prime }l^{\prime }\right)w_{k}c_{hkl,k,n}^{*}c_{h^{\prime }k^{\prime }l^{\prime },k,n}Y_{i}\left({Û}_{k+G_{hkl}}\right)Y_{j}\left({Û}_{k+G_{h^{\prime }k^{\prime }l^{\prime }}}\right)e^{-iG_{HKL}\cdot R}{\overset{¯}{\mathrm{p̃}}}_{i}\left(\left|k+G_{hkl}\right|\right){\overset{¯}{\mathrm{p̃}}}_{j}\left(\left|k+G_{h^{\prime }k^{\prime }l^{\prime }}\right|\right)$$ 12

Then, the effective-nonlocal energy contribution to the MJHP is found by substituting the MJHP-density matrix into eq 10:

13 $$MJHP_{HKL,k,n}^{\mathrm{eff-nonlocal}}=\underset{ij}{\sum }\rho_{HKL,ij,k,n}^{MJHP}D_{ij}$$

One final point requires our attention. The $D_{ij}^{1}-{\mathrm{D̃}}_{ij}^{1}$ terms in the nonlocal *D*_*ij*_ matrix account for the total on-site energetic contributions. Therefore, the spherical kinetic energy (*E* ^1,*kinetic*^; see the Supporting Information) contributions from the all-electron and pseudo-partial waves are also contained in this term. In the MJHP, our focus is on the potential energy contributions from pairs of planewaves, so the on-site kinetic energy must be removed from the effective-nonlocal energy. Removing these terms yields the kinetic-energy-corrected effective-nonlocal energy:

14 $$MJHP_{HKL,k,n}^{\mathrm{eff-nonlocal,corr}}=\underset{ij}{\sum }\left(\rho_{HKL,ij,k,n}^{MJHP}D_{ij}-\rho_{HKL,ij,k,n}^{MJHP}K_{n_{i},l_{i},n_{j},l_{j}}\delta_{l_{i},l_{j}}\delta_{m_{i},m_{j}}\right)$$

where *n*_*i*_, *l*_*i*_, and *m*_*i*_ are the *nlm* atomic orbital quantum numbers associated with partial wave *i*; $K_{n_{i},l_{i},n_{j},l_{j}}$ is the on-site kinetic energy matrix (defined in the Supporting Information); and the Kronecker deltas restrict mixing to occur only between partial waves with the same angular momentum quantum numbers. The total MJHP for crystal orbital *n* at *k*-point **k** is found by simply summing the effective-local potential energy and the corrected effective-nonlocal energy terms:

15 $$MJHP_{HKL,k,n}=MJHP_{HKL,k,n}^{\mathrm{eff-local}}+MJHP_{HKL,k,n}^{\mathrm{eff-nonlocal,corr}}$$

### 3.2 Application of the PAW-MJHP

With this definition of the PAW-MJHP, we can now explore the MJ effects in the PAW electronic structures of intermetallic systems using the tools we developed previously. First, a broad assessment of the potential MJ effects in a system can be obtained by resolving the MJHP by band energy and diffraction angle:

16 $$MJHP\left(E,2\theta \right)=\underset{HKL}{\sum }\underset{k,n}{\sum }w_{k}MJHP_{HKL,k,n}\;\delta \left(\varepsilon_{k,n}-E\right)\delta \left(2\theta_{HKL}-2\theta \right)$$

where {ε_**k**,*n*_} are the band energies and the sharpness of the δ-functions is mitigated during visualization using height-conserving, normalized Gaussian functions. The *MJHP*(*E*, 2θ) collects the potential energy contributions from different reciprocal lattice vectors according to their wavevector magnitudes, $\left|G_{HKL}\right|=\frac{2\pi }{d_{HKL}}$ , and converts them to degrees 2θ using Bragg’s Law. The conversion allows them to be viewed in the style of a powder diffraction pattern, with one key difference: whereas diffraction intensities are always ≥0, the *MJHP*(*E*, 2θ) values are positive and negative for destabilizing and stabilizing interactions, respectively. This analysis is performed specifically at *k*-points that map to the centers of Jones Zone faces for the reciprocal lattice vectors with non-negligible diffraction intensity.

Once the key reciprocal lattice vectors have been identified, we perform a more focused assessment of the MJ effects at those points through the calculation of curves of the form:

$$MJHP_{HKL}\left(E\right)=\underset{HKL}{\sum }\underset{k,n}{\sum }w_{k}MJHP_{HKL,k,n}\delta \left(\varepsilon_{k,n}-E\right)$$ 17

The *MJHP*_*HKL*_(*E*) function collects the potential energy from pairs of planewaves for which the wavevectors for both waves lie within the spherical shell defined in eq 5. Here, a very dense *k*-point mesh is used to ensure a high resolution of the subtle effects of the frontier orbitals responsible for the MJ interactions. The curves are plotted as *–MJHP*_*HKL*_(*E*), so that stabilizing interactions appear on the right, following the tradition of the Crystal Orbital Overlap and Crystal Orbital Hamilton Populations.^1,84–86^ Finally, the integration of the MJHP allows us to quantify the contributions of the MJ effects across different families of reciprocal lattice vectors:

18 $$iMJHP_{HKL}=\frac{1}{N_{atoms}}\int_{-\infty }^{E_{F}}MJHP_{HKL}\left(E\right)dE$$

where *N*_*atoms*_ is the number of atoms in the unit cell.

## 4 Atomic Dataset Optimization

We have now seen how the definition of the MJHP can be adapted for PAW-DFT calculations. As we turn to the application of this method, however, some care must be given to the way atoms are modeled with the PAW formulation. To illustrate this point, we consider elemental Zn, whose Mg (hcp)-type structure has previously been connected to MJ stabilization.^32^ In Figure 1a, we plot the DOS distribution and the *MJHP*_*HKL*_(*E*) curve for the {011} family of reciprocal lattice vectors calculated with the HGH norm-conserving pseudopotentials (HGH-NCPPs) using the original version of the MJHP analysis (the corresponding *MJHP*(*E*, 2θ) plot is given in the Supporting Information). The *E*_F_ lies directly in a pseudogap, a sign of potential MJ effects, and the *MJHP*_*HKL*_(*E*) curve for the {011} family of reciprocal lattice vectors possesses strong stabilizing and destabilizing potential energy contributions below and above the *E*_F_, respectively. The *iMJHP* value of −19.04 meV/atom confirms the stabilization bestowed by the MJ effect.

**Figure 1:**
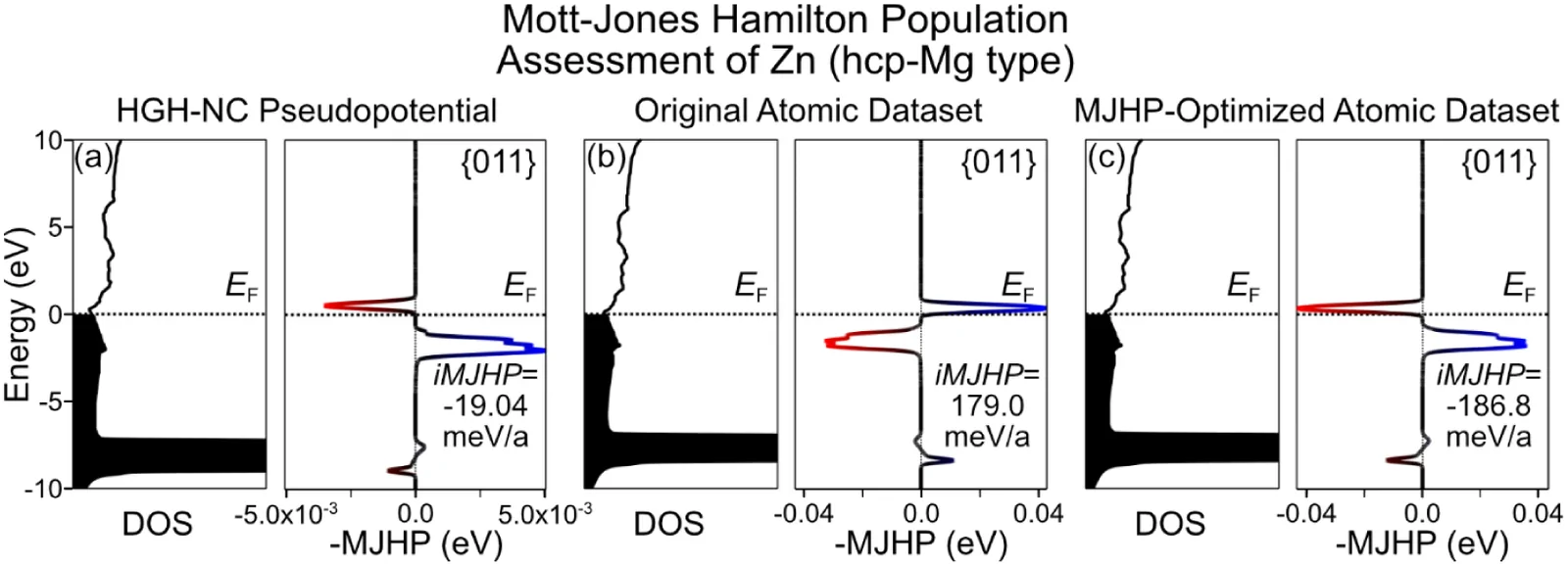
Comparison of MJHP results for elemental (hcp-Mg type) Zn. DOS distribution and *MJHP*_*HKL*_(*E*) curve for (a) the HGH norm-conserving pseudopotential implementation of the MJHP, (b) the PAW version of the MJHP calculated using the original JTH atomic dataset, and (c) the PAW-MJHP results found using an atomic dataset optimized for this purpose. meV/atom is abbreviated to meV/a in the MJHP plots.

In Figure 1b, we plot the corresponding DOS distribution and *MJHP*_*HKL*_(*E*) function calculated with the PAW method using an atomic dataset for Zn taken from the well-established JTH package of PAW atomic datasets that includes most of the stable elements of the periodic table.^75^ The overall curvature of the DOS and *MJHP*_*HKL*_(*E*) plots between the two DFT models are strikingly similar, indicating good matching in the reconstruction of the overall electronic structure of Zn. However, there is one profound difference: the signs of the *MJHP*_*HKL*_(*E*) curve are inverted. The curve changes from destabilizing below the *E*_F_ to stabilizing above the *E*_F_. While there is clearly a relationship between planewave interactions and the pseudogap in the DOS distribution, the contributions appear backward. How could such a different result arise? As shown in the previous section, the chief differences between the HGH-NCPP and PAW definitions of the MJHP appear in the *E* ^*eff*‑*nonlocal*^ term, where in the latter, the parameters of the PAW atomic datasets play a prominent role.

The PAW atomic datasets contain all the necessary parameters to accurately reconstruct the all-electron wave functions from a set of valence pseudo-wavefunctions in a DFT calculation, such as the all-electron partial waves, pseudo-partial waves, projector functions, and radial grids. The merit of a PAW atomic dataset is judged primarily based on three factors: (1) the Δ-value, a measure of the difference between the equation of states derived from ground-state PAW calculations performed using the dataset and reference all-electron calculations; (2) the alignment of the logarithmic derivatives of the pseudo-radial wavefunctions, $D_{l}\left(E\right)={\frac{\partial \log(R_{nl}\left(E,r\right))}{\partial r}|}_{r=r_{c}}$ (where *r*_*c*_ is the radius of the augmentation sphere), with the logarithmic derivatives for the all-electron radial wavefunctions; and (3) the computational efficiency, quantified by the magnitude of the *ecut* parameter needed to converge the energy with respect to the planewave basis set. Within this scope of requirements, there exists a nearly infinite combination of reasonable input parameters that govern the production of the atomic datasets.

During the generation of the PAW atomic datasets, the logarithmic derivatives of the all-electron and pseudo-radial wavefunctions are revealing. The poles, visible as sharp features in the all-electron wavefunctions, represent bound states. The matching of the pseudo-wavefunction curves to the all-electron wavefunction curves is representative of the pseudo-radial wavefunctions’ ability to accurately reconstruct the scattering potential of the atomic orbitals. Better alignment between these curves indicates a higher degree of accuracy in the reconstruction.

In Figure 2a, we show the logarithmic derivatives of the all-electron and pseudo-radial wavefunctions for the original atomic dataset (oAD) of Zn from the JTH set in black and green, respectively. The pseudo-radial wavefunctions for all three angular momentum channels align closely to their all-electron counterparts at low energies. Tracing the curves at higher energies, however, we find unexpected oscillations in the pseudo-wavefunction curves following the *4s* and *4p* bound states. These features, highlighted with green stars, represent bound states at the spurious eigenvalues of the pseudo-Hamiltonian and lead to erroneous scattering properties. They are known as ghost states.^87,88^ Their presence above the occupied atomic orbitals is not concerning for general applications, e.g., the calculation of the total energy. The MJHP, though, is focused on the states at the Fermi energy, so more consideration must be given to the behavior of the datasets at higher energies.

**Figure 2:**
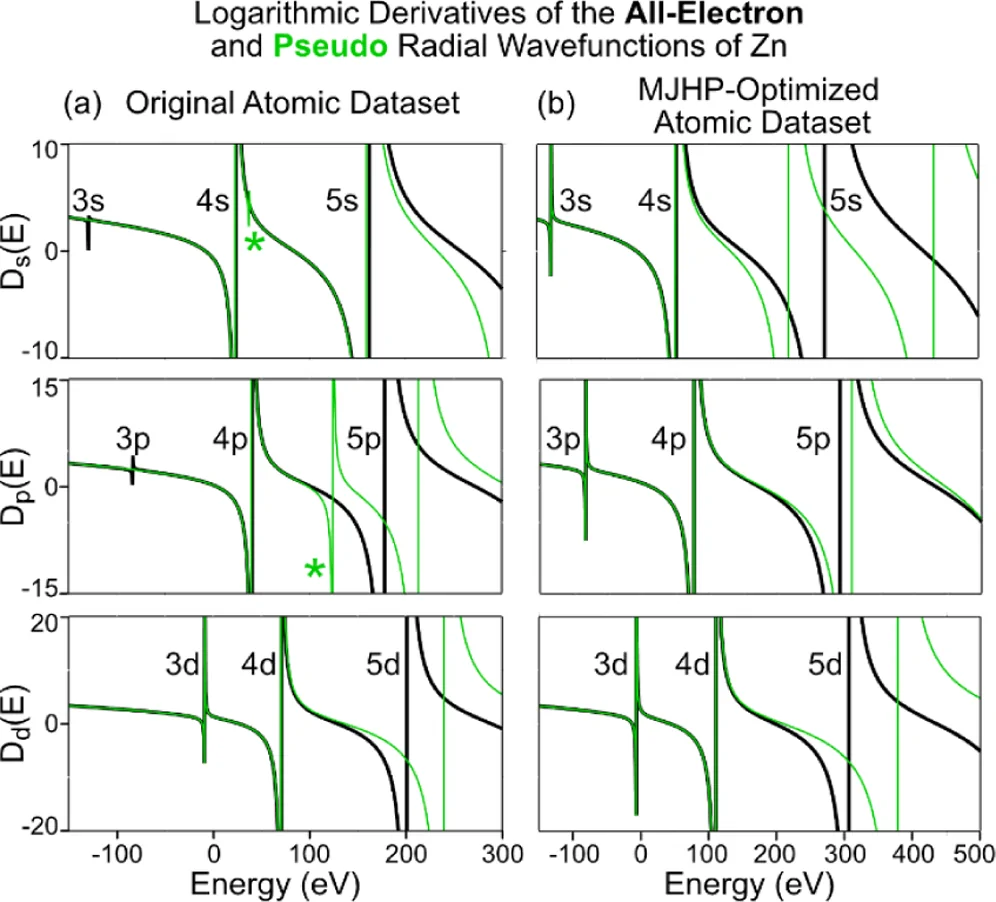
Logarithmic derivatives of the all-electron (black) and pseudo (green) radial wavefunctions for atomic Zn found with (a) the original PAW atomic dataset and (b) the MJHP-optimized atomic dataset.

The possible presence of ghost states is not the only way that the parametrization of an atomic dataset can influence the MJHP results. In the PAW-DFT method, different atomic datasets can distribute energy across the potential energy terms and planewave pair combinations differently. For example, broader, softer projector functions may increase computational efficiency but will also tend to map low-energy planewaves onto more atomic orbital character. Conversely, projector functions that capture more of the sharper features of an all-electron wavefunction will be less poised to conflate the orbital and free-electron character into a smooth background effect.

To investigate this variability, we developed a program to partition the band energy into contributions from pairs of planewaves and bin them according to the magnitude of the reciprocal lattice vector, |**G**_*HKL*_|, corresponding to the difference of their wavevectors:

19 $$E^{band}\left(\left|G_{HKL}\right|\right)=\underset{k,n}{\sum }\underset{hkl}{\sum }\underset{h^{\prime }k^{\prime }l^{\prime }}{\sum }\left(E_{k,n,h-h^{\prime },k-k^{\prime },l-l^{\prime }}^{kinetic}\;\delta \left(\left|G_{h-h^{\prime },k-k^{\prime },l-l^{\prime }}\right|-\left|G_{HKL}\right|\right)+E_{k,n,h-h^{\prime },k-k^{\prime },l-l^{\prime }}^{eff‐local}\;\delta \left(|G_{h-h^{\prime },k-k^{\prime },l-l^{\prime }}\right|-\left|G_{HKL}\right|\right)+E_{k,n,h-h^{\prime },k-k^{\prime },l-l^{\prime }}^{eff‐nonlocal}\;\delta \left(\left|G_{h-h^{\prime },k-k^{\prime },l-l^{\prime }}\right|-\left|G_{HKL}\right|\right))$$

where the δ-function bins the energetic contributions by the magnitude of the reciprocal lattice vector.

When the **|G**_000_| (infinite wavelength) contribution dominates, a large function of the band energy is attributed to the smooth background, resulting in softer pseudo-wavefunctions at the cost of hiding some of the sharper all-electron orbital features. In contrast, when the band energy is more distributed to other |**G**_*HKL*_| (shorter wavelength, higher frequency) terms, more localized, sharp all-electron features can be recovered from the augmentation region.

In Figure 3a, we plot the *E* ^*band*^(|**G**_*HKL*_|) function for Zn calculated with the oAD, including in the sum all bands within ±10 eV of the *E*_F_. The energy contributions are binned such that the |**G**_*HKL*_| values are rounded up to the nearest integer and truncated at 10 (see the Supporting Information for the untruncated plot). Almost 60% of the total band energy between the energy range of −10 and 10 eV is placed in the smooth |**G**_000_| term. Therefore, over half of the total band energy resides in the smooth background, meaning a simple planewave contribution to a pseudo-wavefunction can imply significant atomic orbital character. In this situation, it is difficult to model the nearly-free electron states envisioned by the MJ model.

**Figure 3:**
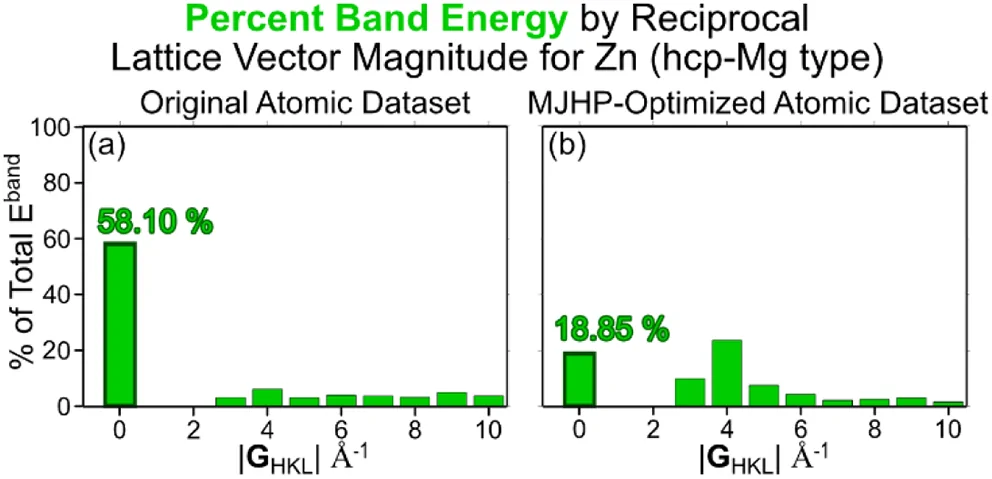
Percent of the band energy, summed from all bands within ±10 eV of the *E*_F_, contributed by reciprocal lattice vectors of different lengths in (hcp-Mg type) Zn for (a) the original atomic dataset and (b) the MJHP-optimized atomic dataset. The lengths of the reciprocal lattice vectors are rounded up to the nearest integer.

Having noted the presence of ghost states and the heavy reliance on the low |**G**_*HKL*_| vectors in the oAD for Zn, let’s set out to calculate a new atomic dataset for Zn that addresses these issues. One avenue to accomplish this goal is to incorporate more all-electron orbital character into the PAW atomic datasets by treating more semicore states as part of the valence set. Toward this end, we promote all *n_val_* – 1 atomic orbitals to valence orbitals, such that our new atomic dataset for Zn incorporates 20 electrons in the valence set [3s^2^4s^2^3p^6^3d^10^] compared to only 12 electrons in the oAD [4s^2^3d^10^]. Secondary levers for tuning the atomic datasets to incorporate more all-electron orbital character include switching the algorithm used to generate the pseudo-wavefunctions (*ps_scheme* in the *atompaw* program), changing the scheme used to calculate the all-electron effective potential (*Vloc_scheme*), and lowering the radius used to generate the pseudopotential from the all-electron effective potential. Following these modifications for Zn, we systematically varied the input parameters to minimize the differences between the equation of states for ground-state DFT calculations conducted with the new dataset and reference all-electron calculations.

An examination of the logarithmic derivatives of the all-electron and pseudo-radial wavefunctions for the new dataset in Figure 2b reveals an absence of ghost states in the vicinity of the frontier *4s* and *4p* orbitals while maintaining a similar degree of matching between the curves as compared to the oAD. In addition, the |**G**_*HKL*_|-binned band energies (Figure 3b) show a drastic reduction in the **G**_000_ term to less than 20% of the band energy compared to almost 60% in the oAD. Therefore, more energetic weight is being associated with the shorter-wavelength, higher-frequency reciprocal lattice vectors, suggesting a better recovery of the more localized, all-electron-like features in direct space.

How does this adjustment of the atomic dataset affect the *MJHP*_*HKL*_(*E*) analysis? In Figure 1c, we replot the DOS distribution and the *MJHP*_*HKL*_(*E*) curve for the {011} family of reciprocal lattice vectors in Zn metal, this time calculated using the new atomic dataset. The general curvature of these plots is similar to that of the HGH-NCPPs and the PAW oAD results, indicating an accurate reconstruction of the overall electronic structure. However, the *MJHP*_*HKL*_(*E*) curve now has a more intuitive arrangement of the stabilizing and destabilizing potential energy features. Stabilization now appears below *E*_F_, while destabilization is seen above *E*_F_. By removing the presence of ghost states and redistributing the energetic terms toward a more all-electron-like picture, the characteristic stabilizing-to-destabilizing splitting in the MJHP can be extracted.

The process we just followed for Zn suggests that additional criteria should be applied to the PAW atomic datasets when considering their suitability for MJHP analysis. First, the atomic dataset must accurately reconstruct the scattering potential of not only the occupied electronic states but also the higher energy electronic states as well. Second, the AD must capture more of the sharp all-electron orbital effects by minimizing how much of the total energy is smoothed into the background. We refer to PAW parametrizations that meet these criteria as MJHP-optimized atomic datasets (MJHPoAD). Of the remaining nine elements explored in this article (Al, Ni, Cu, Zn, Ga, Pd, Ag, Cd, Pt, and Au), all except Cu and Pd required a reoptimization of their JTH atomic datasets to satisfy our additional criteria, as detailed in the Supporting Information.

These revised potentials offer the opportunity to investigate the generality of the MJ-derived pseudogap at 2 electrons/atom observed specifically for elemental Zn in Figure 1. In the Supporting Information, we show the corresponding results for hypothetical hcp-type forms of elemental Ga, Pd, and Ag. In each case, an analogous DOS minimum and crossing in the {011} MJHP curve from stabilizing to destabilizing contributions are detectable at 2 electrons per atom. The mismatch of their actual Fermi energies from this optimized band filling is consistent with none of these elements adopting the hcp structure under ambient conditions.

## 5 Results and Discussion

In the previous sections, we constructed the PAW implementation of the MJHP and explored the influence of the PAW atomic datasets on the MJHP results for elemental Zn. Let’s now explore how the MJHP results evolve in a series of binary test cases: CuZn (CsCl-type), AgCd (CsCl-type), and AlNi_3_ (AuCu_3_-type). As we will see, the overall electron structure features remain invariant across the different codes and atomic datasets. However, differences in the character and strength of the MJ effects highlight variations between the DFT methods and emphasize the need for the MJHP-optimized PAW atomic datasets.

### 5.1 Comparison of the MJHP Results for CsCl-Type CuZn and AgCd

We now consider how the changes to the Zn atomic dataset manifest on going from the element to compounds. A model system is provided by β-brass, CuZn, a CsCl-type phase (Figure 4a) whose stability is commonly ascribed to the MJ effect (additional results for the CsCl-type NiAl, AuAl, and PdGa are given in the Supporting Information). In Figure 5a, we review our previous MJHP analysis of this compound with the HGH norm-conserving pseudopotential (HGH-NCPP) DFT method. An initial sign for potential MJ effects is the broad pseudogap in the DOS that spans the *E*_F_. The *MJHP*(*E*, 2θ) plot (middle) associates this pseudogap with strong stabilizing (blue) and destabilizing (red) potential energy contributions below and above the *E*_F_, respectively, from the {110} family of reciprocal lattice vectors. These features match the diffraction angle of the hypothetical Fermi sphere diameter, 2θ_*FS*_, for the valence electron concentration of 1.5 electrons/atom in CuZn. The *MJHP*_*HKL*_(*E*) curve (right) confirms the stabilizing MJ effects and the correlation of the pseudogap with the optimization of the MJ interactions for the {110} family of reciprocal lattice vectors, with an integrated *MJHP*_*HKL*_ (*iMJHP*_HKL_) value of −23.4 meV/atom.

**Figure 4:**
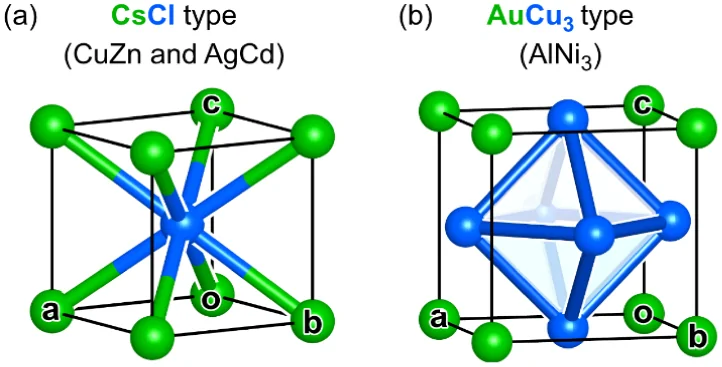
Crystal structures used for illustrating the PAW-MJHP method: (a) the CsCl type, as adopted by CuZn and AgCd, and (b) the AuCu_3_ type, as exemplified by AlNi_3_.

**Figure 5:**
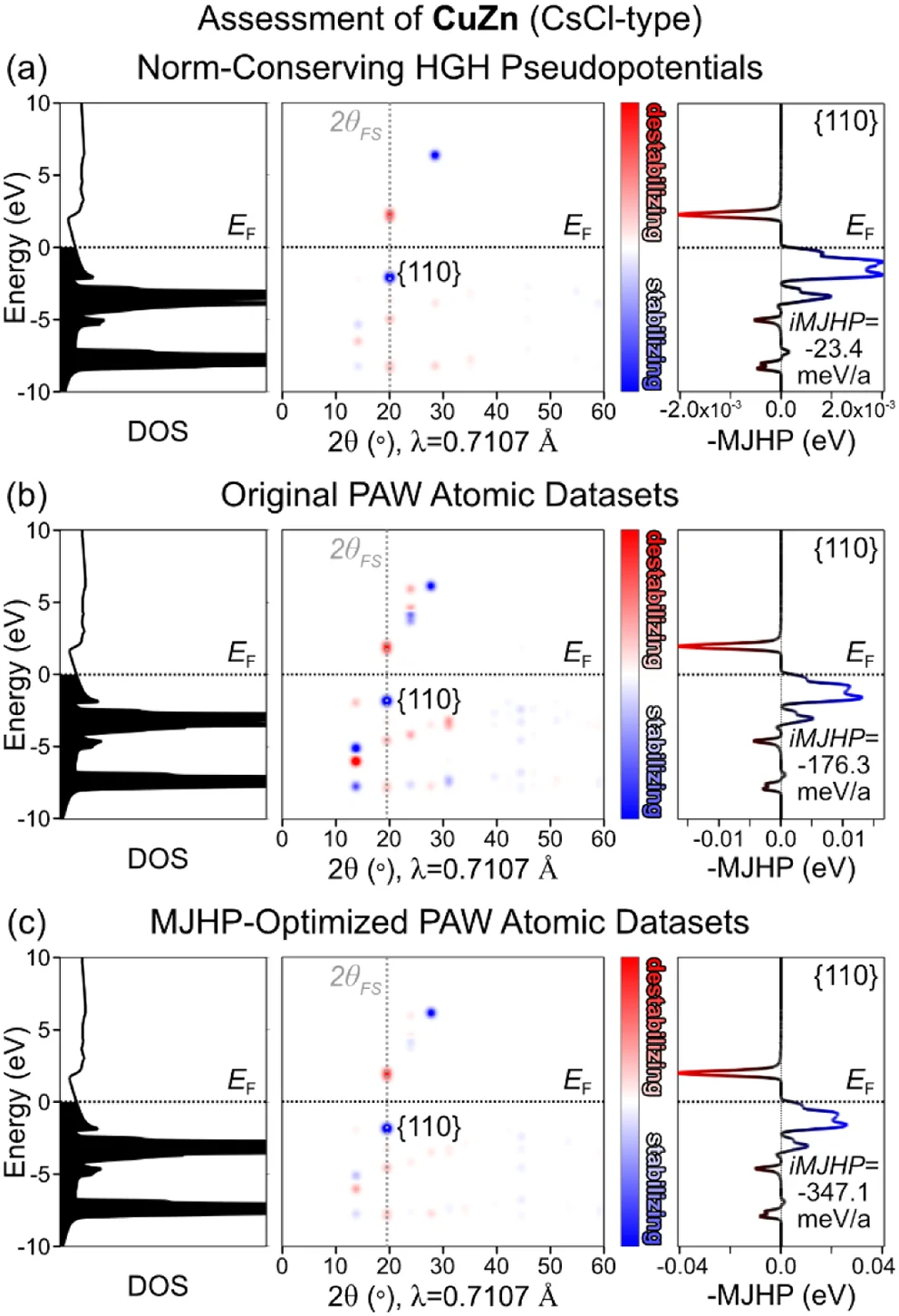
Comparison of MJHP results in CuZn (CsCl-type). DOS distribution, *MJHP*(*E*, 2θ) plot, and *MJHP_HKL_* (*E*) curve for (a) the HGH norm-conserving pseudopotential implementation of the MJHP, (b) the PAW version of the MJHP calculated using the original atomic datasets, and (c) the PAW-MJHP results found using atomic datasets optimized for this purpose.

In Figure 5b, we consider the PAW-MJHP results obtained with the original atomic dataset (oAD). These results largely agree with the HGH-NCPP implementation, particularly the alignment of the pseudogap in the DOS distribution with the strong pair of stabilizing and destabilizing contributions from the {110} family of reciprocal lattice vectors in the *MJHP*_*HKL*_(*E*) curve. The reversal that we saw for elemental Zn with the oAD is not observed here. In fact, the magnitude of the stabilizing effect has increased significantly, with the *iMJHP*_*HKL*_ value now being −176.3 meV/atom.

How does incorporating the MJHP-optimized atomic dataset for Zn affect these results (as we noted above, the atomic dataset for Cu required no adjustments)? In Figure 5c, we plot the MJHP results for CuZn obtained using the MJHP-optimized PAW atomic dataset. The features in the electronic structure highlighted previously persist in the MJHP-optimized PAW atomic dataset results. However, the *iMJHP*_*HKL*_ value has now increased in magnitude to a value of −347.1 meV/atom. This increase can be attributed to the optimization of the Zn atomic dataset, where the inclusion of more of the all-electron orbital character shifts the bands’ energy to the higher magnitude reciprocal lattice vectors, resulting in more intense MJHP features. This change illustrates how different DFT methods and atomic datasets distribute the energy and energetic terms differently, mapping varying amounts of atomic orbital character (or none in the case of HGH-NCPP) to different planewaves. As such, the *iMJHP*_*HKL*_ values are best used within the context of a single system to compare the MJ effects across different families of reciprocal lattice vectors.

So far, we have established the validity of the PAW-MJHP method and the need for MJHP-optimized atomic datasets, but given the increased all-electron character made explicit in the MJHP-optimized datasets, is there any computational gain relative to the HGH-NCPPs? In Table 1, we report the computational costs, as measured by the central processing unit (CPU) time in hours, required to perform the self-consistent ground-state DFT calculations needed for the MJHP analysis. The CPU time for the HGH-NCPP calculation of CuZn requires over 60% more computational time than that needed for either of the PAW calculations, highlighting the large basis set sizes needed to converge the sharp features in the wavefunctions of the norm-conserving pseudopotentials. Across the two PAW calculations, the MJHP-optimized atomic dataset requires around 35% more CPU time as compared to the oAD. This increase in computational resources can be understood from the inclusion of more electrons in the valence set of the MJHP-optimized atomic dataset for Zn, leading to a higher number of bands and harder pseudo-wavefunctions.

**Table 1:** CPU Time for DFT Calculations on CuZn (CsCl-Type)

Structure	Calc. type	CPU time (h)
CuZn (CsCl-type)	HGH-NCPP	145.89
CuZn (CsCl-type)	PAW, Original AD	67.49
CuZn (CsCl-type)	PAW, Optimized AD	90.87

Similar trends are found for AgCd, another CsCl-type compound, but with different elements. We begin with the HGH-NCPP MJHP analysis of AgCd in Figure 6a, where we recognize the familiar signatures of the MJ effect: strong stabilizing and destabilizing potential energy contributions below and above the *E*_F_, respectively, from the {110} family of reciprocal lattice vectors. These peaks align with the broad pseudogap around the *E*_F_ in the DOS distribution. The stabilizing MJ effect leads to an *iMJHP*_*HKL*_ value of −15.2 meV/atom, around the same magnitude found for CuZn using this method.

**Figure 6:**
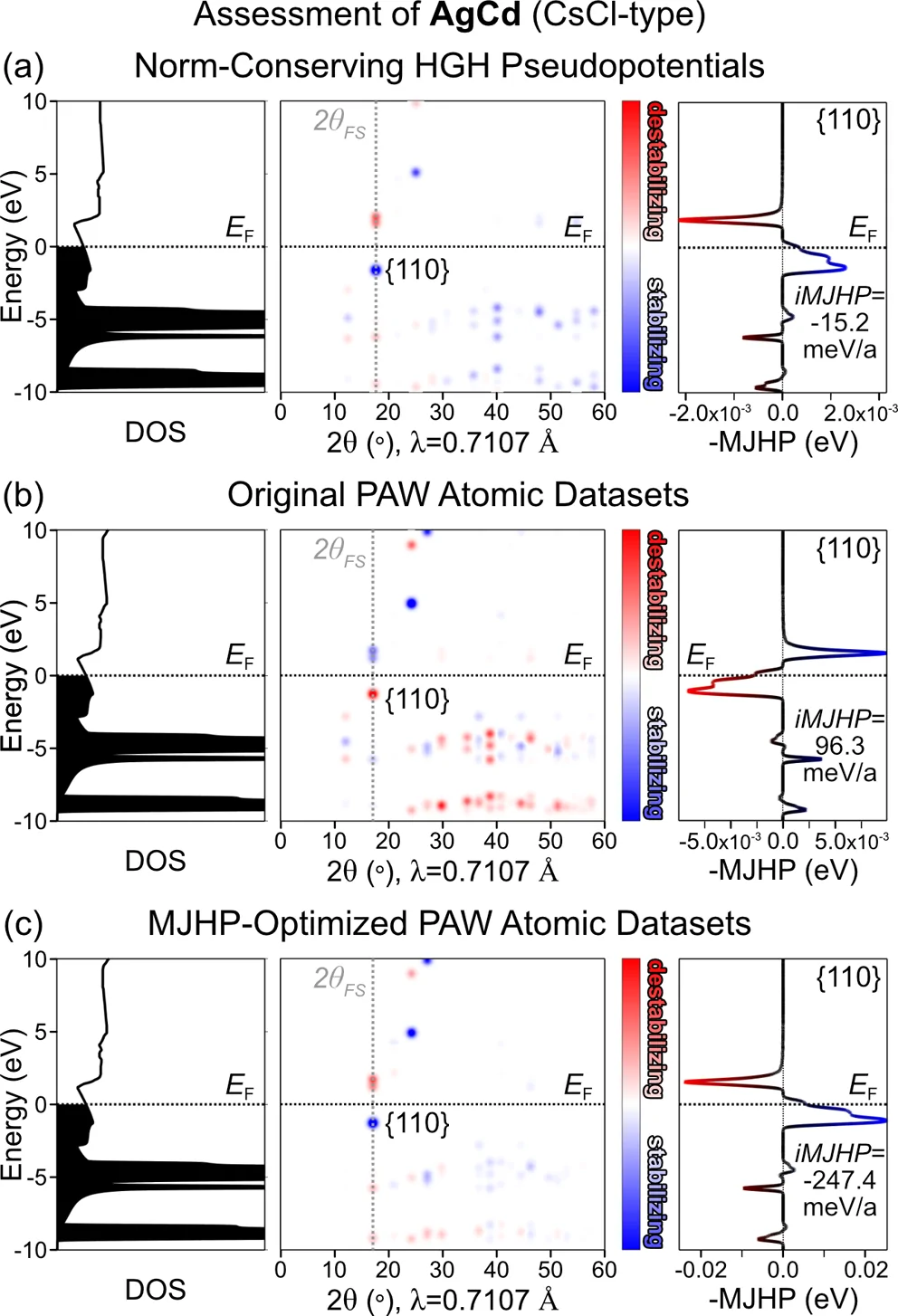
Comparison of MJHP results for CsCl-type AgCd using (a) HGH norm-conserving pseudopotentials, (b) PAW with the original atomic datasets, and (c) PAW using the atomic datasets optimized for the MJHP analysis.

In Figure 6b, we plot the PAW-MJHP results for AgCd found by using the oADs for both Ag and Cd. Like elemental Zn (hcp-Mg type), but unlike CuZn, we see a complete inversion of the signs of the *MJHP*_HKL_(*E*) curve for the {110} family of vectors, with strong destabilizing potential energy contributions below the *E*_F_ splitting to strong stabilizing contributions above the *E*_F_. This manifests in a positive *iMJHP*_*HKL*_ value of 96.3 meV/atom, suggesting the MJ effect is working to maximize destabilizing planewave interactions below the *E*_F_, a counterintuitive result. It appears that our additional criteria for PAW atomic datasets are required.

This conclusion is supported by the PAW-MJHP analysis of AgCd done using the MJHP-optimized atomic datasets, as shown in Figure 6c. The pseudogap at the *E*_F_ in the DOS distribution and the overall shape of the *MJHP_HKL_* (*E*) curve for the {110} family of vectors are strikingly similar to those in the oAD results. However, the potential energy contributions now switch from stabilizing contributions below the *E*_F_ to destabilizing contributions above the *E*_F_ with an *iMJHP*_*HKL*_ value of −247.4 meV/atom. This value is on the same order of magnitude as that obtained for elemental Zn and CsCl-type CuZn using the MJHP-optimized atomic datasets.

The computational costs of the DFT calculations for AgCd are listed in Table 2. Like CuZn, the PAW calculation using the oADs requires significantly less CPU time than the HGH-NCPP method. However, the time required for the MJHP-optimized PAW calculation is similar to that of the HGH-NCPP calculation. This time difference can be reconciled by examining the number of electrons included in the atomic valence set. The HGH-NCPPs and the oADs treat 11 and 12 valence electrons in Ag and Cd, respectively, whereas the MJHP-optimized atomic datasets have higher counts of 19 and 20 valence electrons. The expanded valence sets in the MJHP-optimized atomic datasets require more bands and harder pseudo-wavefunctions, resulting in longer computational times.

**Table 2:** Computational Costs for Calculations on AgCd (CsCl-Type)

Structure	Calc. type	CPU time (h)
AgCd (CsCl-type)	HGH-NCPP	54.37
AgCd (CsCl-type)	PAW, Original AD	25.17
AgCd (CsCl-type)	PAW, Optimized AD	55.38

### 5.2 Comparison of the MJHP Results in AuCu_3_-Type AlNi_3_

Having considered some cases where the application of the MJ model is straightforward, let’s turn to a system with a more complicated situation: AlNi_3_ (AuCu_3_-type, Figure 4b; see the Supporting Information for corresponding results for other instances of this type: AuCu_3_ itself and PdGa_3_).^67^ This structure is the primitive variant of a face-centered cubic lattice with different colorings of the cell corner (Al) and face-center (Ni) positions. Its valence electron concentration of 0.75 electrons/atom (considering the Al and Ni atoms as bringing 3 and 0 valence electrons, respectively) is significantly lower than the CsCl-type structures we analyzed above.

We begin by seeing what can be learned from the original HGH-NCPP-based analysis. In Figure 7a, we plot the DOS distribution for the HGH-NCPP calculation of AlNi_3_. An initial sign for potential MJ effects is the deep, narrow pseudogap near *E*_F_. However, this is not the only interesting feature of the DOS. Two other, higher energy pseudogaps are also evident: a broader pseudogap spanning 1 to 4 eV above the *E*_F_ and a second, shallower pseudogap around 6 to 8 eV above the *E*_F_. Could these DOS features be connected to MJ effects from different families of reciprocal-lattice vectors?

**Figure 7:**
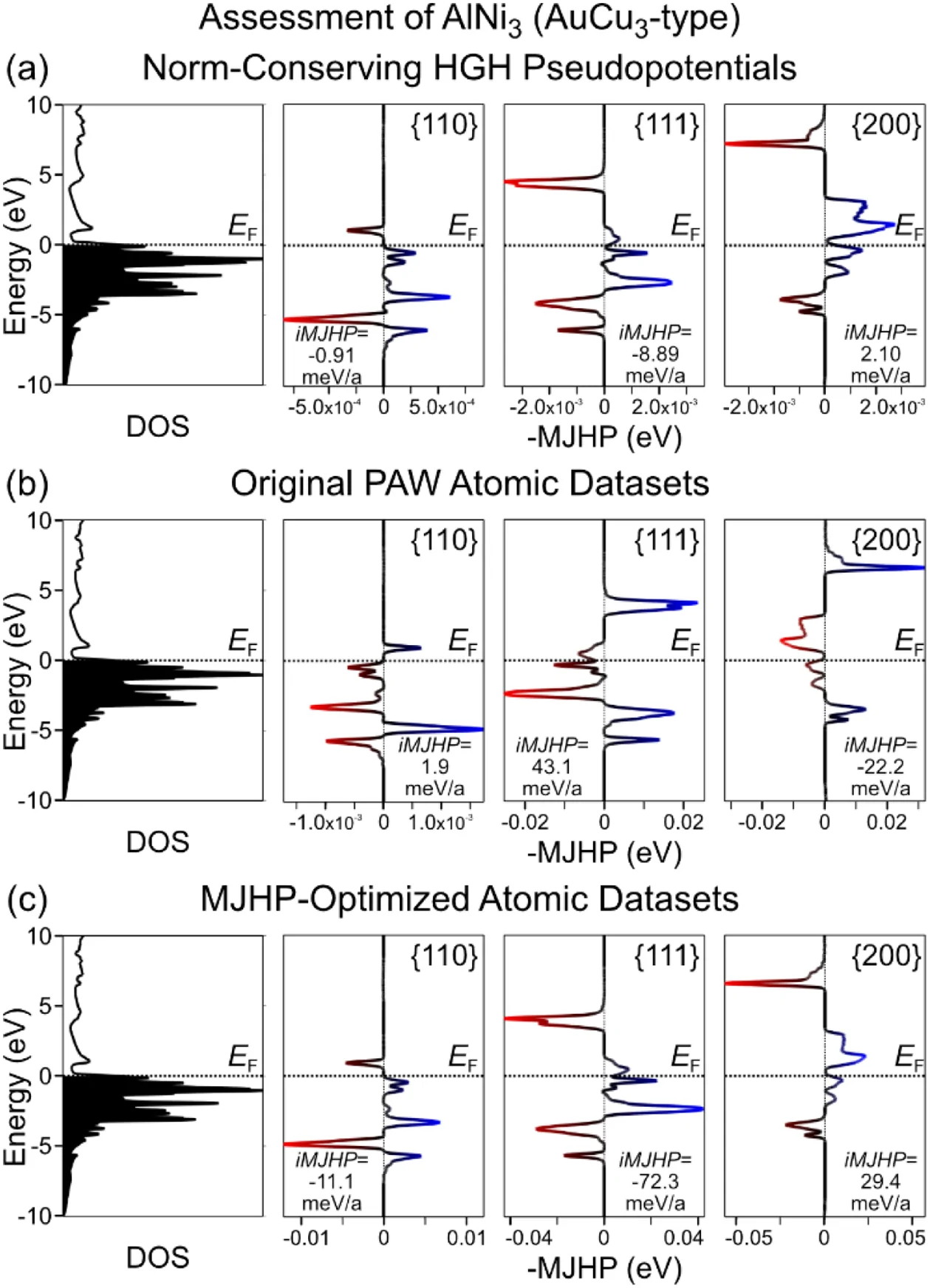
DOS distribution, and *MJHP*_*HKL*_(*E*) curves for the {110}, {111}, and {200} family of vectors for AlNi_3_ (AuCu_3_-type) calculated using (a) the HGH norm-conserving pseudopotentials, (b) PAW with the original atomic datasets, and (c) PAW with the MJHP-optimized atomic datasets.

To explore this connection, we plot the *MJHP*_*HKL*_(*E*) curves for the reciprocal lattice vectors that couple planewaves lying closest to the hypothetical free-electron Fermi sphere of AlNi_3_ (see the Supporting Information for the corresponding *MJHP*(*E*, 2θ) plots). The shortest of these vectors, the {110} family, has stabilizing and destabilizing potential energy contributions directly below and above the *E*_F_, respectively, that align with the upper limit and lower shoulder of the first pseudogap. These contributions result in a relatively small *iMJHP*_*HKL*_ value of −0.91 meV/atom. Moving to the {111} family of vectors, we see stronger stabilizing potential energy contributions below and slightly above the *E*_F_ with strong, sharp destabilizing contributions around 4 eV above the *E*_F_. The placement of the *E*_F_ relative to this splitting is not optimal, falling just shy of the top of the stabilizing peaks. A closer examination of the DOS shows a similar trend, with the *E*_F_ lying slightly below the first pseudogap minima. In fact, like the {110} family of vectors, the bottom of the highest stabilizing peak aligns with the lower edge of the first pseudogap, but, unlike the {110} vectors, the top of the destabilizing peak coincides with the upper edge of the second pseudogap. In effect, the strong splitting from the {111} family of reciprocal lattice vectors extends beyond the splitting from the weaker {110} family of vectors, forming the lower limits of the first pseudogap and the upper limits of the second pseudogap.

The final family of reciprocal lattice vectors we consider, {200}, has the longest lengths of those that are poised to interact with the Fermi sphere. The *MJHP*_*HKL*_(*E*) curve for the {200} family of reciprocal lattice vectors has stabilizing and destabilizing potential energy contributions of about 2 and 8 eV above the *E*_F_, respectively, with comparable strengths to those of the {111} vectors. As the integrals for the *iMJHP*_*HKL*_ values only go up to the *E*_F_, little of the potential stabilization from the MJ effect is included, leading to the positive *iMJHP*_*HKL*_ value of 2.10 meV/atom. Returning to the DOS distribution, the maximum in the DOS separating the first and second pseudogaps align with the bottom of the upper block of stabilizing contributions from the {200} family of vectors. The gentle sloping of the second pseudogap matches the remainder of the broad stabilizing contributions, and finally, the strong, sharp destabilizing potential energy contribution coincides in energy with the top of the third pseudogap.

In this analysis, the DOS features at and above the *E*_F_ can be traced to MJ effects from different reciprocal lattice vectors, where the minima in the DOS represent opportunities for stabilization at different valence electron concentrations. For example, if the system were more electron-rich, the Fermi sphere would lie closer to the planewaves coupled through the {200} family of vectors, and the *E*_F_ would be higher in the DOS distribution lying near the second or third pseudogap. Therefore, this structure appears to offer a variety of MJ effects at different valence-electron concentrations.

Let’s compare these results to those from the PAW method using the oAD (Figure 7b) and the MJHP-optimized atomic datasets (Figure 7c). The trends observed previously in elemental Zn and CsCl-type AgCd persist here. Specifically, in the oAD results, the *MJHP*_*HKL*_(*E*) curves for all three families of reciprocal lattice vectors are reversed, leading to positive *iMJHP*_*HKL*_ values for the {110} and {111} families of reciprocal lattice vectors and a negative value for the {200} family. In contrast, when the PAW calculation is performed using the MJHP-optimized atomic datasets, the more intuitive splitting in the *MJHP*_HKL_(*E*) curves is observed, with stabilizing MJ effects occurring below the destabilizing effects. The *iMJHP*_*HKL*_ values of −11.1 meV/atom and −72.3 meV/atom for the {110} and {111} vectors, respectively, reflect this splitting. In all three calculations, the *iMJHP*_*HKL*_ value for the {110} family is around an order of magnitude smaller compared to the *iMJHP*_*HKL*_ value for the {111} vectors.

The situation regarding computational costs for the AlNi_3_ DFT calculations (Table 3) is reminiscent of that obtained for AgCd. Going from the HGH-NCPP method to PAW with the oAD yields significant savings in terms of CPU time. These gains are largely lost, however, when adopting the MJHP-optimized PAW atomic datasets. Even so, these datasets allow the MJHP analysis to be used with the PAW formalism without significantly adding computational time, removing the reliance on a specific pseudopotential formulation, and making the method easier to adopt by new users.

**Table 3:** Comparison of the Computational Hours Needed for the AlNi_3_ (AuCu_3_-Type) DFT Calculations

Structure	Calc. type	CPU time (h)
AlNi_3_ (AuCu_3_-type)	HGH-NCPP	110.45
AlNi_3_ (AuCu_3_-type)	PAW, Original AD	62.12
AlNi_3_ (AuCu_3_-type)	PAW, Optimized AD	100.03

## 6 Conclusions

The Mott–Jones Hamilton Population (MJHP) is a theoretical framework that connects MJ interactions between planewaves to a system’s energetics. However, the broader application of this method has faced barriers associated with its lack of transferability and use of hard pseudopotentials that require large basis sets and have low adoption within the solid-state and materials chemistry community. In this article, we have derived and implemented the Projector Augmented Wave (PAW) version of the MJHP to overcome these limitations.

The PAW implementation overcomes many of the challenges of the HGH-NCPP-based method. The mutability of the PAW atomic datasets allows us to track the influence of the atomic dataset parametrization on the MJHP results. In elemental Zn (hcp-Mg type), for example, the stabilizing and destabilizing splitting of the MJ effect is inverted depending on the form of the atomic dataset. From this analysis, a series of guiding principles for the generation of atomic datasets optimized for use with the MJHP were established: there should be no ghost states near the frontier orbitals, and the system’s total band energy should be distributed away from the lowest wavevector reciprocal lattice vectors. These conditions are met chiefly by including more electrons in the valence set.

The influence of the PAW atomic datasets and agreement with the HGH-NCPP version of the MJHP is further verified through three example systems: CuZn (CsCl-type), AgCd (CsCl-type), and AlNi_3_ (AuCu_3_-type). Across these cases, the results of the PAW-MJHP analysis using the MJHP-optimized atomic datasets align closely with their HGH-NCPP method counterparts, illustrating the transferability of these results across different DFT models. In the CsCl-type examples, the strength of the {110} family of reciprocal lattice vectors was emphasized, confirming earlier results. In AlNi_3_, three sets of reciprocal lattice vectors, {110}, {111}, and {200}, were connected to significant DOS features at and above the *E*_F_. These pseudogaps at different electron fillings point to this structure’s potential for electronic stabilization at a variety of valence electron concentrations, an idea we are excited to explore further in future research.

A second potential advantage of the PAW method is enhanced computational efficiency that results from the use of softer pseudo-wavefunctions. This hope is affirmed by the computational costs, as measured by the total CPU time, for the DFT calculations on the binary systems using the original atomic datasets. However, when the atomic datasets optimized for the MJHP analysis are used, the computational time increases due to the larger valence sets and higher number of bands. This increase in CPU time results in similar computational costs as for the HGH-NCPP DFT calculations but greater compatibility with the methodology used for most modern DFT calculations on materials and more electrons explicitly modeled.

Over the course of our case studies here, we have sampled all three of the simple sphere packings commonly adopted by metals: bcc (parent structure to the CsCl-type adopted by CuZn and AgCd), hcp (elemental Zn), and fcc (parent structure to the AuCu_3_ type formed by AlNi_3_). The MJ effect appears to play an important energetic role in each case. However, it is notable that while the bcc case was dominated by a single reciprocal lattice vector {110}, the fcc-based case offered a variety of potential lattice vectors that could be used to achieve electronic stabilization at different electron concentrations. It will be interesting to see if this distinction extends to a broad range of compounds based on these two arrangements, and whether it has implications for the emergence of superstructures (e.g., γ-brass from the bcc structure).

## Supplementary Material



## Notes

Additional equations used in the derivation of the PAW-MJHP method; computational details and optimized geometries of crystal structures; analysis of MJHP-optimized atomic datasets; *MJHP*(*E*, 2θ) plots for selected structures; PAW-MJHP results for additional Mg (hcp)-, CsCl-, and AuCu_3_-type structures; dependence of MJHP results for CuZn on the values of the Δ_*shell*_ and σ parameters (PDF)
