# Supplementary material for: Interactions on the Fermi Sphere in an All-Electron World: The Projector Augmented Wave Implementation of the Mott–Jones Hamilton Population and the Three Archetypal Sphere Packings
Source: J Phys Chem C Nanomater Interfaces. 2026 Sep 8;130(38):13273–86. doi: 10.1021/acs.jpcc.6c02214 (PMC13618042; doi:10.1021/acs.jpcc.6c02214)
Supplement: Supplementary file 1 [file jp6c02214_si_001.pdf]

SUPPORTING INFORMATION

Interactions on the Fermi Sphere in an All-Electron World: The Projector Augmented Wave Implementation of the Mott-Jones Hamilton Population and the Three Archetypical Sphere Packings

Leah C. Garman and Daniel C. Fredrickson*

Department of Chemistry, University of Wisconsin-Madison, 1101 University Avenue, Madison, Wisconsin 53706, United States

*email: danny@chem.wisc.edu

S1. Additional Equations Used in the Derivation of the PAW-MJHP Analysis

The derivation of the effective-nonlocal potential energy requires the spherical projector functions, originally defined on the radial grids about each atomic site, to be transformed onto the reciprocal space grid. This is accomplished by performing a radial Fourier transform of the projector functions:

$$\bar{\tilde{p}}_i(|\mathbf{k}+\mathbf{G}_{hkl}|) = \int_0^{r_c^{\mathbf{R}}} r^2 dr\, \tilde{p}_i(r) j_l(|\mathbf{k}+\mathbf{G}_{hkl}|r) \quad \text{(S1)}$$

where $j_l(|\mathbf{k}+\mathbf{G}_{hkl}|r)$ is the spherical Bessel function for the angular momentum quantum number *l*, *r* is the radial grid index, and $r_c^{\boldsymbol{R}}$ is the radius of the augmentation region for atomic site **R**. The transformed projector function, $\bar{\tilde{p}}_i(|\mathbf{k}+\mathbf{G}_{hkl}|)$, is then applied in the calculation of the effective-nonlocal energy defined in equation 11 of the main text.

This effective-nonlocal energy term must be corrected by removing the spherical on-site kinetic energy contributions, $E^{1,kinetic}$:

$$E^{1,kinetic} = \sum_{\boldsymbol{k},n} w_{\boldsymbol{k}} o_{n,\boldsymbol{k}} \sum_{ij} \rho_{ij} K_{n_i,l_i,n_j,l_j} \delta_{l_i,l_j} \delta_{m_i,m_j} \quad \text{(S2)}$$

where K_{n_i,l_i,n_j,l_j} is the kinetic energy difference between the all-electron and pseudo-partial waves and the Kronecker deltas ensure mixing only occurs between partial waves with the same angular momentum quantum numbers. When using the program *atompaw*, the K_{n_i,l_i,n_j,l_j} matrix is output in the atomic dataset file for each element.

S2. Computational Details and Optimized Geometries for the Elemental Crystal Structures

Table S1. Computational details for the geometrical optimizations using ABINIT.

Structure	Atomic Dataset[a]	Coarse E Cutoff (Ha)	Fine E Cutoff (Ha)	*k*-point Mesh[b]	No. *k*-points	Coarse FFT Grid	Fine FFT Grid	Total E/Cell (Ha)
Al (fcc)	original	28	28	12×12×12	72	30×30×30	30×30×30	-2.1059
Al (fcc)	optimized	28	28	12×12×12	72	30×30×30	30×30×30	-81.6042
Ni (fcc)	original	44	44	12×12×12	72	36×36×36	36×36×36	-170.8689
Ni (fcc)	optimized	44	44	12×12×12	72	36×36×36	36×36×36	-170.8700
Cu (fcc)	original	24	24	12×12×12	72	27×27×27	27×27×27	-197.9641
Zn (hcp)	HGH-NC	135	-	16×16×8	150	54×54×96	-	-120.9532
Zn (hcp)	original	38	38	16×16×8	150	36×36×64	36×36×64	-128.6241
Zn (hcp)	optimized	38	38	16×16×8	150	36×36×64	36×36×64	-455.4459
Ga (α form)	original	22	22	8×8×8	105	45×45×45	45×45×45	-316.3104
Ga (α form)	optimized	22	22	8×8×8	105	45×45×45	45×45×45	-1040.6203
Pd (fcc)	original	24	48	12×12×12	72	30×30×30	40×40×40	-128.8211
Ag (fcc)	original	24	24	12×12×12	72	30×30×30	30×30×30	-38.2215
Ag (fcc)	optimized	24	24	12×12×12	72	30×30×30	30×30×30	-147.9674
Cd (hcp)	original	32	32	16×16×8	150	36×36×64	36×36×64	-95.1061
Cd (hcp)	optimized	32	32	16×16×8	150	36×36×64	36×36×64	-337.6037
Pt (fcc)	original	24	24	12×12×12	72	30×30×30	30×30×30	-27.0238
Pt (fcc)	optimized	24	24	12×12×12	72	30×30×30	30×30×30	-121.3692
Au (fcc)	original	26	26	12×12×12	72	30×30×30	30×30×30	-34.1561
Au (fcc)	optimized	26	26	12×12×12	72	30×30×30	30×30×30	-137.7858

[a]In this and all following tables of ABINIT calculation parameters, "HGH-NC" refers to calculations performed with the HGH norm-conserving pseudopotentials, "original" refers to PAW calculations

performed using the original JTH atomic datasets, and "optimized" refers to PAW calculations performed using the atomic datasets optimized for use with the MJHP.

[b]For all calculations, the *k*-point shift was set to 0.0×0.0×0.0.

Table S2. Computational details for ABINIT calculations used for the MJHP and DOS distribution analysis.

Structure	Atomic Dataset	Coarse E Cutoff (Ha)	Fine E Cutoff (Ha)	*k*-point Mesh	No. *k*-points	Coarse FFT Grid	Fine FFT Grid	Total E/Cell (Ha)
Al (fcc)	original	28	28	32×32×32	897	27×27×27	27×27×27	-2.1056
Al (fcc)	optimized	28	28	32×32×32	897	27×27×27	27×27×27	-8.1604
Ni (fcc)	original	44	44	32×32×32	897	30×30×30	30×30×30	-170.8688
Ni (fcc)	optimized	44	44	32×32×32	897	30×30×30	30×30×30	-170.8699
Cu (fcc)	original	24	24	32×32×32	897	24×24×24	24×24×24	-197.9640
Zn (hcp)	HGH-NC	135	-	32×32×16	918	54×54×96	-	-120.9528
Zn (hcp)	original	38	38	32×32×16	918	30×30×54	30×30×54	-128.6238
Zn (hcp)	optimized	38	38	32×32×16	918	30×30×54	30×30×54	-455.4457
Ga (α form)	original	22	22	16×16×16	657	36×36×40	36×36×40	-316.3106
Ga (α form)	optimized	22	22	16×16×16	657	36×36×40	36×36×40	-1040.6206
Pd (fcc)	original	24	48	32×32×32	897	24×24×24	36×36×36	-128.8210
Ag (fcc)	original	24	24	32×32×32	897	27×27×27	27×27×27	-38.2216
Ag (fcc)	optimized	24	24	32×32×32	897	27×27×27	27×27×27	-147.9675
Cd (hcp)	original	32	32	32×32×16	918	30×30×60	30×30×60	-95.1059
Cd (hcp)	optimized	32	32	32×32×16	918	30×30×60	30×30×60	-337.6036
Pt (fcc)	original	24	24	32×32×32	897	24×24×24	24×24×24	-27.0238
Pt (fcc)	optimized	24	24	32×32×32	897	24×24×24	24×24×24	-121.3692
Au (fcc)	original	26	26	32×32×32	897	27×27×27	27×27×27	-34.1563
Au (fcc)	optimized	26	26	32×32×32	897	27×27×27	27×27×27	-137.7861

Table S3. Optimized cell parameters from ABINIT, converted to the conventional cell.

Structure	Atomic Dataset	a (Å)	b (Å)	c (Å)	α (°)	β (°)	γ (°)
Al (fcc-Cu type)	original	4.0410	4.0410	4.0410	90	90	90
Al (fcc-Cu type)	optimized	4.0429	4.0429	4.0429	90	90	90
Ni (fcc-Cu type)	original	3.5237	3.5237	3.5237	90	90	90
Ni (fcc-Cu type)	optimized	3.5227	3.5227	3.5227	90	90	90
Cu (fcc-Cu type)	original	3.6245	3.6245	3.6245	90	90	90
Zn (hcp-Mg type)	HGH-NC	2.5838	2.5838	4.7625	90	90	120
Zn (hcp-Mg type)	original	2.6629	2.6629	4.9236	90	90	120
Zn (hcp-Mg type)	optimized	2.6617	2.6617	4.9229	90	90	120

Ga (α-Ga type)	original	7.7286	4.6252	4.5608	90	90	90
Ga (α-Ga type)	optimized	7.7086	4.6255	4.5520	90	90	90
Pd (fcc-Cu type)	original	3.9476	3.9476	3.9476	90	90	90
Ag (fcc-Cu type)	original	4.1518	4.1518	4.1518	90	90	90
Ag (fcc-Cu type)	optimized	4.1465	4.1465	4.1465	90	90	90
Cd (hcp-Mg type)	original	3.0499	3.0499	5.6586	90	90	120
Cd (hcp-Mg type)	optimized	3.0461	3.0461	5.6490	90	90	120
Pt (fcc-Cu type)	original	3.9762	3.9762	3.9762	90	90	90
Pt (fcc-Cu type)	optimized	3.9724	3.9724	3.9724	90	90	90
Au (fcc-Cu type)	original	4.1612	4.1612	4.1612	90	90	90
Au (fcc-Cu type)	optimized	4.1560	4.1560	4.1560	90	90	90

Table S4. Fractional atomic coordinates for the face-centered cubic (fcc-Cu type) elemental structures: Al, Ni, Cu, Pd, Ag, Pt, and Au.

Wyckoff[a]	Atom	*x*	*y*	*z*
4*a*	1	0	0	0
	2	0.5	0.5	0
	3	0.5	0	0.5
	4	0	0.5	0.5

[a]Wyckoff labels are provided for one instance of each symmetry-distinct site in this and all following tables of atomic coordinates.

Table S5. Fractional atomic coordinates for hexagonal close-packed (hcp-Mg type) elemental structures: Zn and Cd.

Wyckoff	Atom	*x*	*y*	*z*
2*c*	1	1/3	2/3	0.25
	2	2/3	1/3	0.75

Table S6. Optimized fractional atomic coordinates for Ga (α-Ga type) found using the original atomic dataset.

Wyckoff	Atom	*x*	*y*	*z*
8*f*	Ga1	0.6563	0	0.5840
	Ga2	0.8437	0	0.0840
	Ga3	0.1563	0	0.9160
	Ga4	0.3437	0	0.4160

Table S7. Optimized fractional atomic coordinates for Ga (α-Ga type) found using the optimized atomic dataset.

Wyckoff	Atom	*x*	*y*	*z*
8*f*	Ga1	0.6563	0	0.5850
	Ga2	0.8437	0	0.0850
	Ga3	0.1563	0	0.9150
	Ga4	0.3437	0	0.4150

S3. Computational Details and Elemental Crystal Structures Used to Perform the Δ-Calculations

The Δ-value provides a single metric to evaluate the reliability of a PAW atomic dataset by comparing the equation-of-states from a reference all-electron calculation and a calculation performed using the PAW atomic dataset:

$$\Delta = \sqrt{\frac{\int_{V_{cell}^{initial}}^{V_{cell}^{final}} \left(E_{reference}(V_{cell}) - E_{PAW}(V_{cell})\right)^2 dV}{V_{cell}^{final} - V_{cell}^{initial}}} \quad \text{(S3)}$$

where $E_{reference}(V_{cell})$ and $E_{PAW}(V_{cell})$ are the energies from the all-electron reference calculation and the PAW calculation, respectively, at the unit cell volume, V_{cell}. In this analysis, the WEIN2k all-electron results are used as a reference. The thresholds for excellent and good agreements with the all-electron reference material are Δ-values of less than 0.3 and 0.95 meV/atom, respectively.

Table S8. Computational details for the Δ-value calculation using ABINIT.

Structure	Atomic Dataset	Coarse E Cutoff (Ha)	Fine E Cutoff (Ha)	*k*-point Mesh	No. *k*-points
Al (fcc-Cu type)	original	40	40	40×40×40	1661
Al (fcc-Cu type)	optimized	40	40	40×40×40	1661
Ni (fcc-Cu type)	original	40	40	40×40×40	1661
Ni (fcc-Cu type)	optimized	40	40	40×40×40	1661
Cu (fcc-Cu type)	original	40	40	40×40×40	1661
Zn (hcp-Mg type)	original	40	40	32×32×32	1734
Zn (hcp-Mg type)	optimized	40	40	32×32×32	1734
Ga (α-Ga type)	original	40	40	30×30×30	3856
Ga (α-Ga type)	optimized	40	40	30×30×30	3856
Pd (fcc-Cu type)	original	40	40	40×40×40	1661
Ag (fcc-Cu type)	original	40	40	40×40×40	1661
Ag (fcc-Cu type)	optimized	40	40	40×40×40	1661
Cd (hcp-Mg type)	original	40	40	32×32×32	1734
Cd (hcp-Mg type)	optimized	40	40	32×32×32	1734
Pt (fcc-Cu type)	original	40	40	40×40×40	1661
Pt (fcc-Cu type)	optimized	40	40	40×40×40	1661
Au (fcc-Cu type)	original	40	40	40×40×40	1661
Au (fcc-Cu type)	optimized	40	40	40×40×40	1661

Table S9. Computational parameters for Al (fcc-Cu type) used during the SCF cycles of the ABINIT calculations for the determination of Δ for the original atomic dataset.

Calculation No.	Volume (Å^3)	Coarse FFT Grid	Fine FFT Grid	Total E/Cell (Ha)
1	13.6941	30×30×30	30×30×30	-57.1271
2	14.5870	30×30×30	30×30×30	-57.2216
3	15.5178	32×32×32	32×32×32	-57.2724
4	16.4874	32×32×32	32×32×32	-57.2874
5	17.4965	32×32×32	32×32×32	-57.2734
6	18.5460	32×32×32	32×32×32	-57.2364
7	19.6367	36×36×36	36×36×36	-57.1815

Table S10. Computational parameters for Al (fcc-Cu type) used during the SCF cycles of the ABINIT calculations for the determination of Δ for the optimized atomic dataset.

Calculation No.	Volume (Å^3)	Coarse FFT Grid	Fine FFT Grid	Total E/Cell (Ha)
1	13.6941	30×30×30	30×30×30	-2220.3890
2	14.5870	30×30×30	30×30×30	-2220.4842
3	15.5178	32×32×32	32×32×32	-2220.5347
4	16.4874	32×32×32	32×32×32	-2220.5500
5	17.4965	32×32×32	32×32×32	-2220.5379
6	18.5460	32×32×32	32×32×32	-2220.4998
7	19.6367	36×36×36	36×36×36	-2220.4451

Table S11. Computational parameters for Ni (fcc-Cu type) used during the SCF cycles of the ABINIT calculations for the determination of Δ for the original atomic dataset.

Calculation No.	Volume (Å^3)	Coarse FFT Grid	Fine FFT Grid	Total E/Cell (Ha)
1	9.0883	27×27×27	27×27×27	-4649.2427
2	9.6808	27×27×27	27×27×27	-4649.4034
3	10.2986	30×30×30	30×30×30	-4649.4868
4	10.9421	30×30×30	30×30×30	-4649.5096
5	11.6118	30×30×30	30×30×30	-4649.4838
6	12.3083	30×30×30	30×30×30	-4649.4196
7	13.0322	30×30×30	30×30×30	-4649.3259

Table S12. Computational parameters for Ni (fcc-Cu type) used during the SCF cycles of the ABINIT calculations for the determination of Δ for the optimized atomic dataset.

Calculation No.	Volume (Å^3)	Coarse FFT Grid	Fine FFT Grid	Total E/Cell (Ha)
1	9.0883	27×27×27	27×27×27	-4649.2734
2	9.6808	27×27×27	27×27×27	-4649.4325
3	10.2986	30×30×30	30×30×30	-4649.5168
4	10.9421	30×30×30	30×30×30	-4649.5391
5	11.6118	30×30×30	30×30×30	-4649.5130
6	12.3083	30×30×30	30×30×30	-4649.4487
7	13.0322	30×30×30	30×30×30	-4649.3536

Table S13. Computational parameters for Cu (fcc-Cu type) used during the SCF cycles of the ABINIT calculations for the determination of Δ for the original atomic dataset.

Calculation No.	Volume (Å^3)	Coarse FFT Grid	Fine FFT Grid	Total E/Cell (Ha)
1	9.9888	27×27×27	27×27×27	-5386.6795
2	10.6401	27×27×27	27×27×27	-5386.8016
3	11.3190	30×30×30	30×30×30	-5386.8615
4	12.0263	30×30×30	30×30×30	-5386.8767
5	12.7624	30×30×30	30×30×30	-5386.8542
6	13.5279	30×30×30	30×30×30	-5386.8015
7	14.3235	30×30×30	30×30×30	-5386.7275

Table S14. Computational parameters for Zn (hcp-Mg type) used during the SCF cycles of the ABINIT calculations for the determination of Δ for the original atomic dataset.

Calculation No.	Volume (Å^3)	Coarse FFT Grid	Fine FFT Grid	Total E/Cell (Ha)
1	12.7501	30×30×54	30×30×54	-1749.8816
2	13.5814	30×30×54	30×30×54	-1749.9620
3	14.4480	30×30×54	30×30×54	-1750.0021
4	15.3508	30×30×54	30×30×54	-1750.0112
5	16.2903	30×30×60	30×30×60	-1749.9954
6	17.2675	30×30×60	30×30×60	-1749.9609
7	18.2830	32×32×60	32×32×60	-1749.9135

Table S15. Computational parameters for Zn (hcp-Mg type) used during the SCF cycles of the ABINIT calculations for the determination of Δ for the optimized atomic dataset.

Calculation No.	Volume (Å^3)	Coarse FFT Grid	Fine FFT Grid	Total E/Cell (Ha)
1	12.7501	30×30×54	30×30×54	-6196.5247
2	13.5814	30×30×54	30×30×54	-6196.6036
3	14.4480	30×30×54	30×30×54	-6196.6426
4	15.3508	30×30×54	30×30×54	-6196.6511
5	16.2904	30×30×60	30×30×60	-6196.6344
6	17.2675	30×30×60	30×30×60	-6196.5993
7	18.2830	32×32×60	32×32×60	-6196.5514

Table S16. Computational parameters for Ga (α-Ga type) used during the SCF cycles of the ABINIT calculations for the determination of Δ for the original atomic dataset.

Calculation No.	Volume (Å^3)	Coarse FFT Grid	Fine FFT Grid	Total E/Cell (Ha)
1	16.9098	48×48×48	48×48×48	-2151.6856
2	18.0122	48×48×48	48×48×48	-2151.7627
3	19.1616	48×48×50	48×48×50	-2151.8032
4	20.3589	50×50×50	50×50×50	-2151.8148
5	21.6050	50×50×54	50×50×54	-2151.8041
6	22.9010	54×54×54	54×54×54	-2151.7762
7	24.2477	54×54×54	54×54×54	-2151.7354

Table S17. Computational parameters for Ga (α-Ga type) used during the SCF cycles of the ABINIT calculations for the determination of Δ for the optimized atomic dataset.

Calculation No.	Volume (Å^3)	Coarse FFT Grid	Fine FFT Grid	Total E/Cell (Ha)
1	16.9098	48×48×48	48×48×48	-7079.0589
2	18.0122	48×48×48	48×48×48	-7079.1330
3	19.1616	48×48×50	48×48×50	-7079.1718
4	20.3589	50×50×50	50×50×50	-7079.1814
5	21.6050	50×50×54	50×50×54	-7079.1690
6	22.9010	54×54×54	54×54×54	-7079.1400
7	24.2477	54×54×54	54×54×54	-7079.0978

Table S18. Computational parameters for Pd (fcc-Cu type) used during the SCF cycles of the ABINIT calculations for the determination of Δ for the original atomic dataset.

Calculation No.	Volume (Å^3)	Coarse FFT Grid	Fine FFT Grid	Total E/Cell (Ha)
1	12.8278	30×30×30	30×30×30	-3505.0440
2	13.6642	30×30×30	30×30×30	-3505.2345
3	14.5361	30×30×30	30×30×30	-3505.3314
4	15.4444	32×32×32	32×32×32	-3505.3555
5	16.3897	32×32×32	32×32×32	-3505.3231
6	17.3728	32×32×32	32×32×32	-3505.2489
7	18.3945	32×32×32	32×32×32	-3505.1440

Table S19. Computational parameters for Ag (fcc-Cu type) used during the SCF cycles of the ABINIT calculations for the determination of Δ for the original atomic dataset.

Calculation No.	Volume (Å^3)	Coarse FFT Grid	Fine FFT Grid	Total E/Cell (Ha)
1	14.9944	30×30×30	30×30×30	-1039.8687
2	15.9720	32×32×32	32×32×32	-1039.9885
3	16.9912	32×32×32	32×32×32	-1040.0471
4	18.0529	32×32×32	32×32×32	-1040.0593
5	19.1579	36×36×36	36×36×36	-1040.0367
6	20.3070	36×36×36	36×36×36	-1039.9883
7	21.5013	36×36×36	36×36×36	-1039.9211

Table S20. Computational parameters for Ag (fcc-Cu type) used during the SCF cycles of the ABINIT calculations for the determination of Δ for the optimized atomic dataset.

Calculation No.	Volume (Å^3)	Coarse FFT Grid	Fine FFT Grid	Total E/Cell (Ha)
1	14.9944	30×30×30	30×30×30	-4026.2160
2	15.9720	32×32×32	32×32×32	-4026.3330
3	16.9912	32×32×32	32×32×32	-4026.3895
4	18.0529	32×32×32	32×32×32	-4026.4001
5	19.1579	36×36×36	36×36×36	-4026.3763
6	20.3070	36×36×36	36×36×36	-4026.3268
7	21.5013	36×36×36	36×36×36	-4026.2587

Table S21. Computational parameters for Cd (hcp-Mg type) used during the SCF cycles of the ABINIT calculations for the determination of Δ for the original atomic dataset.

Calculation No.	Volume (Å^3)	Coarse FFT Grid	Fine FFT Grid	Total E/Cell (Ha)
1	19.1444	32×32×60	32×32×60	-1293.8527
2	20.3925	32×32×60	32×32×60	-1293.9316
3	21.6938	36×36×64	36×36×64	-1293.9707
4	23.0493	36×36×64	36×36×64	-1293.9797
5	24.4601	36×36×64	36×36×64	-1293.9669
6	25.9273	36×36×72	36×36×72	-1293.9389
7	27.4521	36×36×72	36×36×72	-1293.9007

Table S22. Computational parameters for Cd (hcp-Mg type) used during the SCF cycles of the ABINIT calculations for the determination of Δ for the optimized atomic dataset.

Calculation No.	Volume (Å^3)	Coarse FFT Grid	Fine FFT Grid	Total E/Cell (Ha)
1	19.1444	32×32×60	32×32×60	-4593.2082
2	20.3925	32×32×60	32×32×60	-4593.2852
3	21.6938	36×36×64	36×36×64	-4593.3226
4	23.0493	36×36×64	36×36×64	-4593.3300
5	24.4601	36×36×64	36×36×64	-4593.3159
6	25.9273	36×36×72	36×36×72	-4593.2866
7	27.4521	36×36×72	36×36×72	-4593.2473

Table S23. Computational parameters for Pt (fcc-Cu type) used during the SCF cycles of the ABINIT calculations for the determination of Δ for the original atomic dataset.

Calculation No.	Volume (Å^3)	Coarse FFT Grid	Fine FFT Grid	Total E/Cell (Ha)
1	13.0589	30×30×30	30×30×30	-734.8060
2	13.9104	30×30×30	30×30×30	-735.1105
3	14.7980	30×30×30	30×30×30	-735.2678
4	15.7226	32×32×32	32×32×32	-735.3110
5	16.6849	32×32×32	32×32×32	-735.2667
6	17.6858	32×32×32	32×32×32	-735.1567
7	18.7259	36×36×36	36×36×36	-734.9981

Table S24. Computational parameters for Pt (fcc-Cu type) used during the SCF cycles of the ABINIT calculations for the determination of Δ for the optimized atomic dataset.

Calculation No.	Volume (Å^3)	Coarse FFT Grid	Fine FFT Grid	Total E/Cell (Ha)
1	13.0589	30×30×30	30×30×30	-3302.1038

2	13.9104	30×30×30	30×30×30	-3302.3979
3	14.7980	30×30×30	30×30×30	-3302.5482
4	15.7226	32×32×32	32×32×32	-3302.5865
5	16.6849	32×32×32	32×32×32	-3302.5392
6	17.6858	32×32×32	32×32×32	-3302.4269
7	18.7259	36×36×36	36×36×36	-3302.2664

Table S25. Computational parameters for Au (fcc-Cu type) used during the SCF cycles of the ABINIT calculations for the determination of Δ for the original atomic dataset.

Calculation No.	Volume (Å^3)	Coarse FFT Grid	Fine FFT Grid	Total E/Cell (Ha)
1	15.1012	30×30×30	30×30×30	-929.1309
2	16.0858	32×32×32	32×32×32	-929.3208
3	17.1122	32×32×32	32×32×32	-929.4139
4	18.1814	32×32×32	32×32×32	-929.4339
5	19.2943	36×36×36	36×36×36	-929.3998
6	20.4516	36×36×36	36×36×36	-929.3261
7	21.6544	36×36×36	36×36×36	-929.2244

Table S26. Computational parameters for Au (fcc-Cu type) used during the SCF cycles of the ABINIT calculations for the determination of Δ for the optimized atomic dataset.

Calculation No.	Volume (Å^3)	Coarse FFT Grid	Fine FFT Grid	Total E/Cell (Ha)
1	15.1012	30×30×30	30×30×30	-3749.0554
2	16.0858	32×32×32	32×32×32	-3749.2385
3	17.1122	32×32×32	32×32×32	-3749.3265
4	18.1814	32×32×32	32×32×32	-3749.3427
5	19.2943	36×36×36	36×36×36	-3749.3061
6	20.4516	36×36×36	36×36×36	-3749.2298
7	21.6544	36×36×36	36×36×36	-3749.1265

Table S27. Material properties for the elemental crystal structures used in the equation-of-states calculation, determined using the Δ-software package.

Structure	Atomic Dataset	Equilibrium Volume (Å^3/atom)	Bulk Modulus, B (GPa)	$\left.\frac{\partial B}{\partial P}\right\|_{V=V_{eq}}$
Al (fcc-Cu type)	original	16.4701	77.5341	4.639
Al (fcc-Cu type)	optimized	16.4893	77.5339	4.516
Ni (fcc-Cu type)	original	10.8944	199.7785	4.916

Ni (fcc-Cu type)	optimized	10.8959	200.3903	4.772
Cu (fcc-Cu type)	original	11.9148	142.4890	5.032
Zn (hcp-Mg type)	original	15.1832	73.5989	5.373
Zn (hcp-Mg type)	optimized	15.1556	73.7396	5.329
Ga (α-Ga type)	original	20.3321	48.8148	5.297
Ga (α-Ga type)	optimized	20.2425	49.2508	5.238
Pd (fcc-Cu type)	original	15.3358	165.1547	5.524
Ag (fcc-Cu type)	original	17.8444	90.7908	5.813
Ag (fcc-Cu type)	optimized	17.8009	91.0280	5.788
Cd (hcp-Mg type)	original	22.8605	43.5103	6.467
Cd (hcp-Mg type)	optimized	22.7699	44.2114	6.462
Pt (fcc-Cu type)	original	15.6745	248.9293	5.551
Pt (fcc-Cu type)	optimized	15.6323	247.7805	5.481
Au (fcc-Cu type)	original	17.9894	139.6216	6.023
Au (fcc-Cu type)	optimized	17.9241	139.9312	5.985

Table S28. Comparison of the Δ-values and the renormalized Δ-values, Δ_1, for all elements.

Structure	Atomic Dataset	Δ (meV/atom)	Δ_1 (meV/atom)[a]
Al (fcc-Cu type)	original	0.170	0.397
Al (fcc-Cu type)	optimized	0.158	0.369
Ni (fcc-Cu type)	original	0.132	0.182
Ni (fcc-Cu type)	optimized	0.183	0.251
Cu (fcc-Cu type)	original	1.277	2.264
Zn (hcp-Mg type)	original	0.206	0.550
Zn (hcp-Mg type)	optimized	0.649	1.729
Ga (α-Ga type)	original	0.260	0.782
Ga (α-Ga type)	optimized	0.708	2.127
Pd (fcc-Cu type)	original	0.916	1.075
Ag (fcc-Cu type)	original	0.063	0.117
Ag (fcc-Cu type)	optimized	0.860	1.598
Cd (hcp-Mg type)	original	0.197	0.592
Cd (hcp-Mg type)	optimized	0.678	2.021
Pt (fcc-Cu type)	original	1.815	1.397
Pt (fcc-Cu type)	optimized	0.544	0.420
Au (fcc-Cu type)	original	0.524	0.627
Au (fcc-Cu type)	optimized	1.492	1.787

[a]Defined as $\Delta_1 = \frac{V_{ref}B_{ref}}{V_{AE}B_{AE}}\Delta$, where V_{ref} and B_{ref} are the unit cell volume and bulk modulus, respectively, of a reference material (taken as $V_{ref} = 30$ Bohr^3 and $B_{ref} = 100$ GPa). This normalizes all elements to the same reference material, so comparisons can be made across different element types.

Table S29. Averaged Δ-values for the elemental crystal structures modeled with either the original and optimized atomic dataset (Al, Ni, Zn, Ga, Ag, Cd, Pt, and Au).

Atomic Dataset	avg. Δ (meV/atom)	avg. Δ_1 (meV/atom)
original	0.374	0.516
optimized	0.586	1.145

S4. Comparison of the Original and MJHP-Optimized Atomic Datasets

Al (fcc-Cu type)

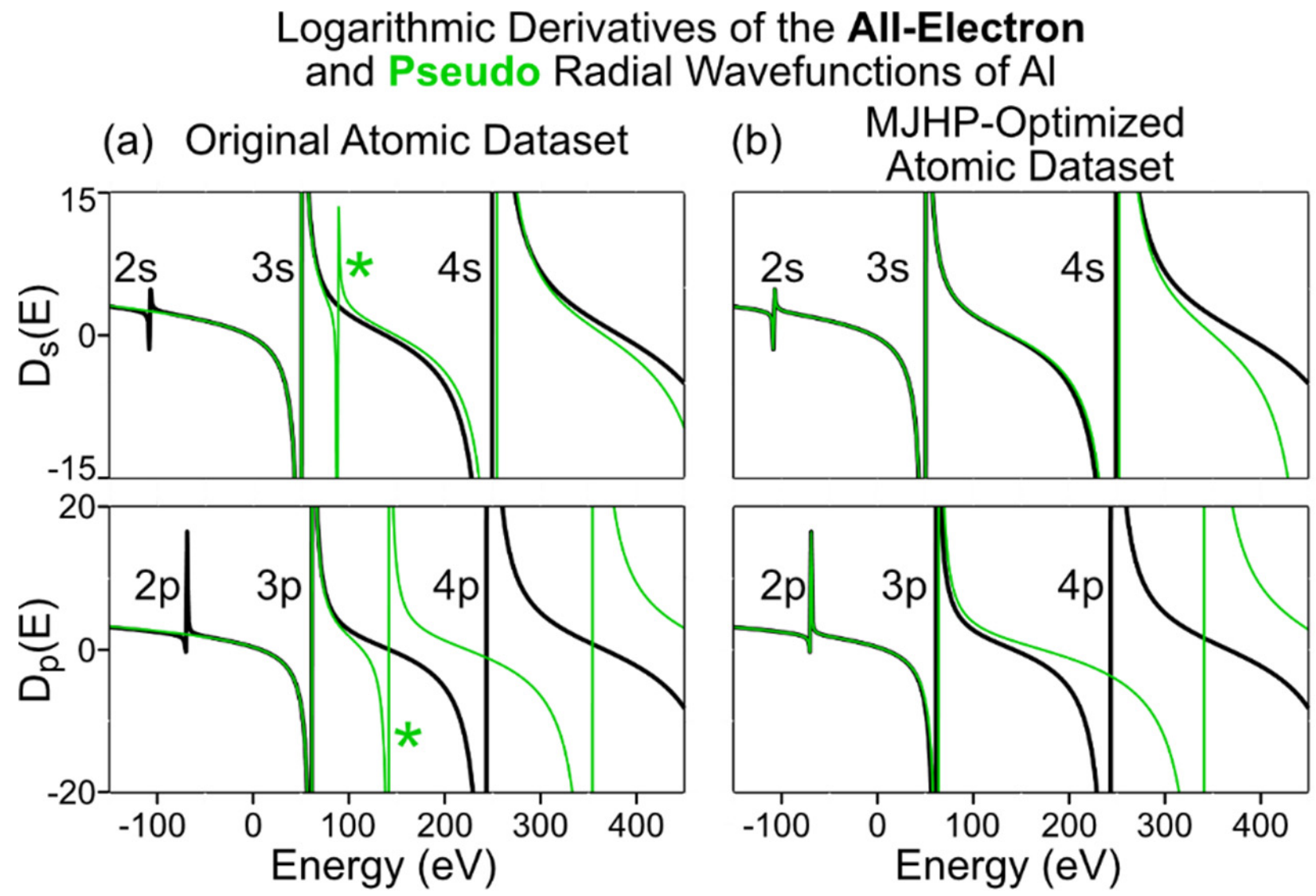

Figure S1. All-electron and pseudo radial wavefunction logarithmic derivative plots for Al (atomic) calculated using the (a) original and (b) optimized atomic dataset.

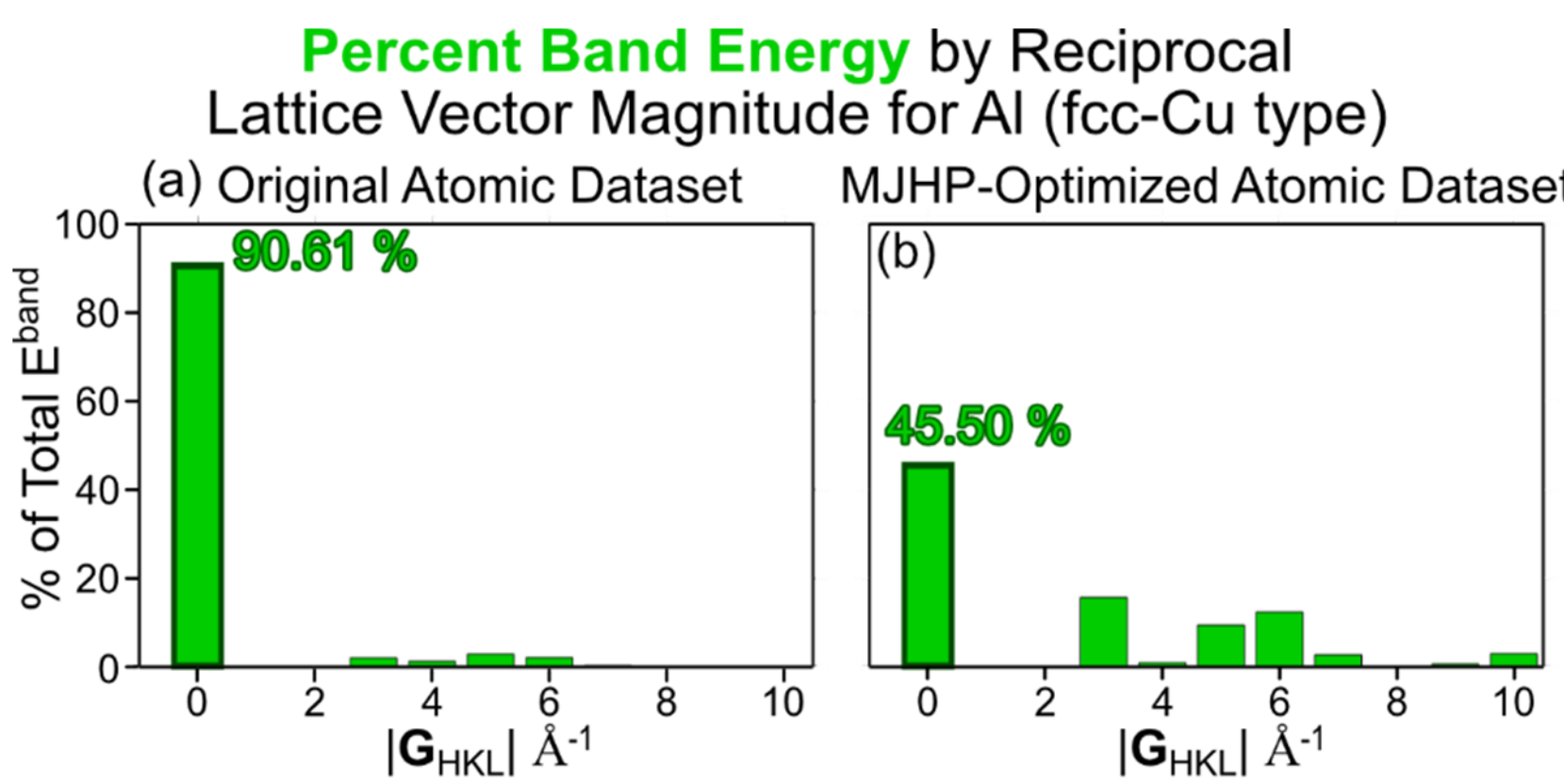

Figure S2. Comparison of the distribution of the percent of the band energy by the reciprocal lattice vector magnitude for Al (fcc-Cu type) calculated using the (a) original and (b) optimized atomic dataset, truncated after $|\mathbf{G}_{HKL}|$ exceeds 10 Å^{-1}.

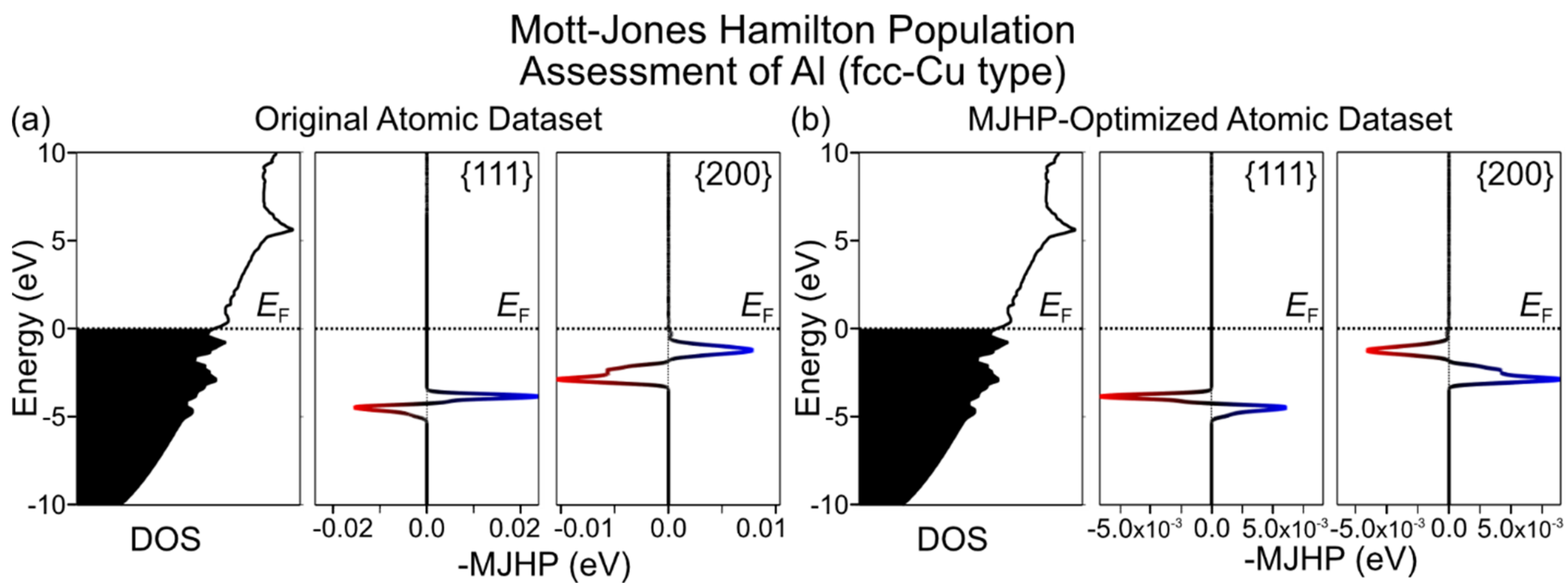

Figure S3. Comparison of the DOS distribution and $MJHP_{HKL}(E)$ curves for the {111} and {200} family of reciprocal lattice vectors in Al (fcc-Cu type) for the (a) original and (b) optimized atomic dataset.

Ni (fcc-Cu type)

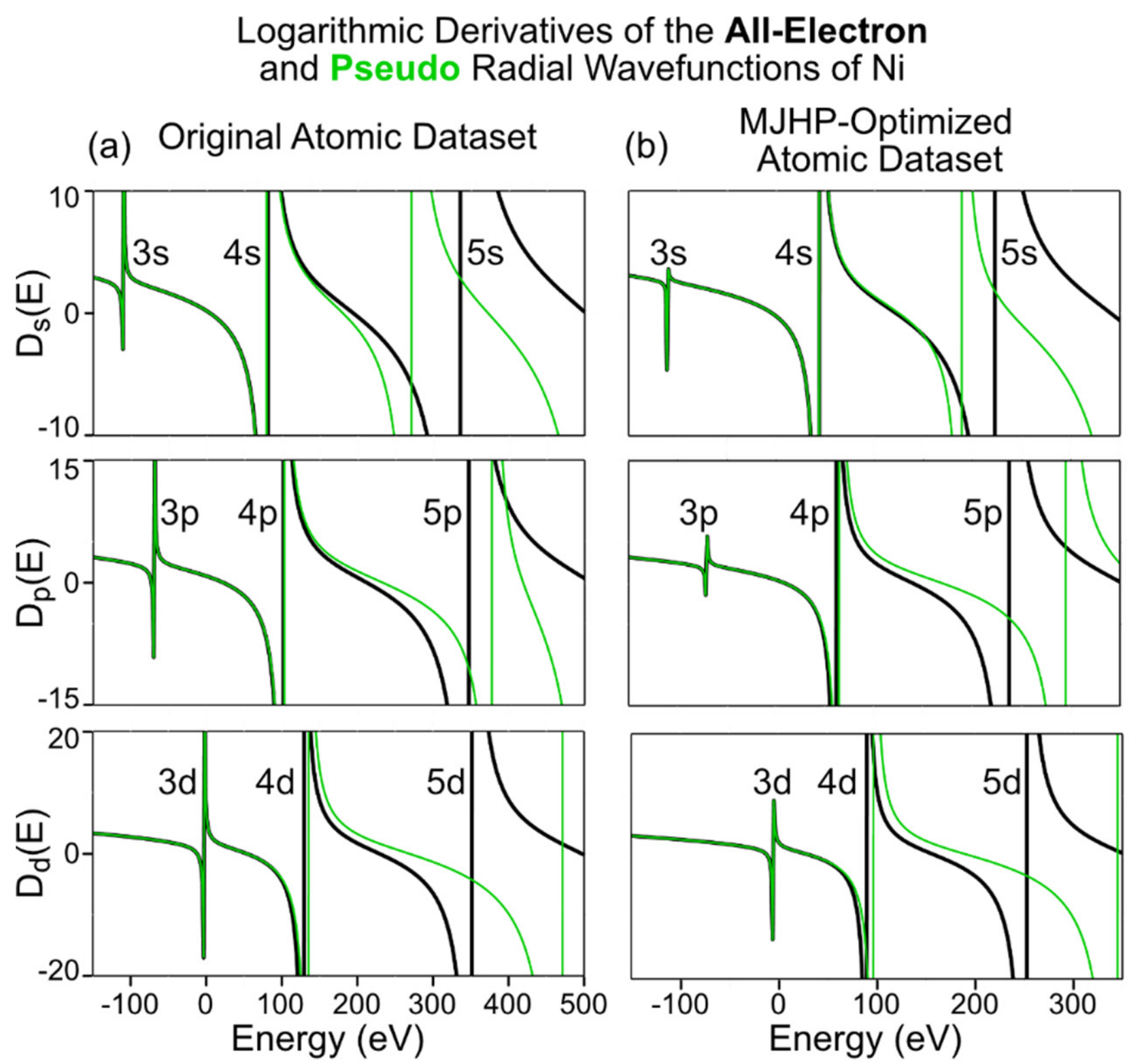

Figure S4. All-electron and pseudo radial wavefunction logarithmic derivative plots for Ni (atomic) calculated using the (a) original and (b) optimized atomic dataset.

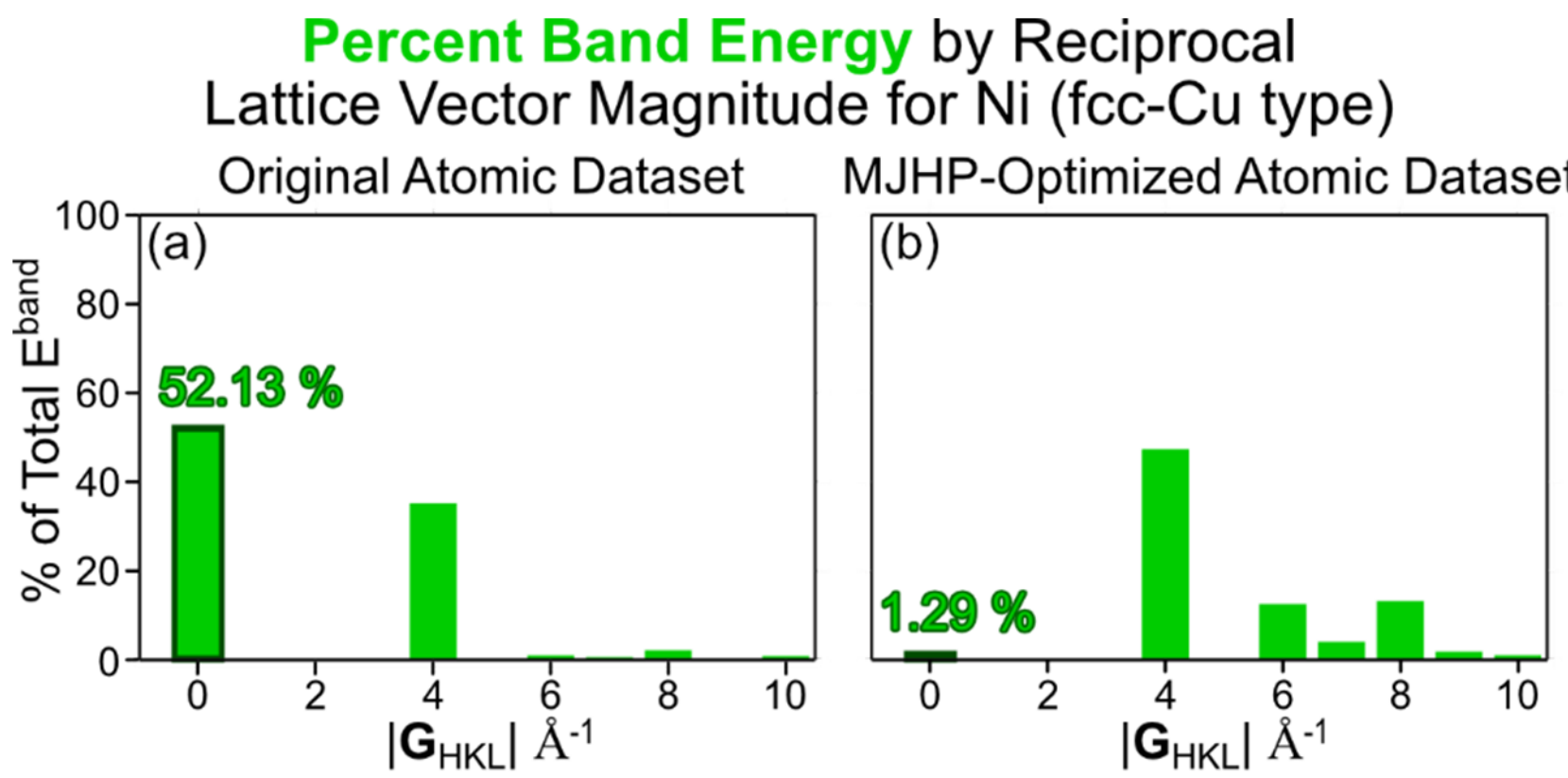

Figure S5. Comparison of the distribution of the percent of the band energy by the reciprocal lattice vector magnitude for Ni (fcc-Cu type) calculated using the (a) original and (b) optimized atomic dataset, truncated after $|\mathbf{G}_{HKL}|$ exceeds 10 Å^{-1}.

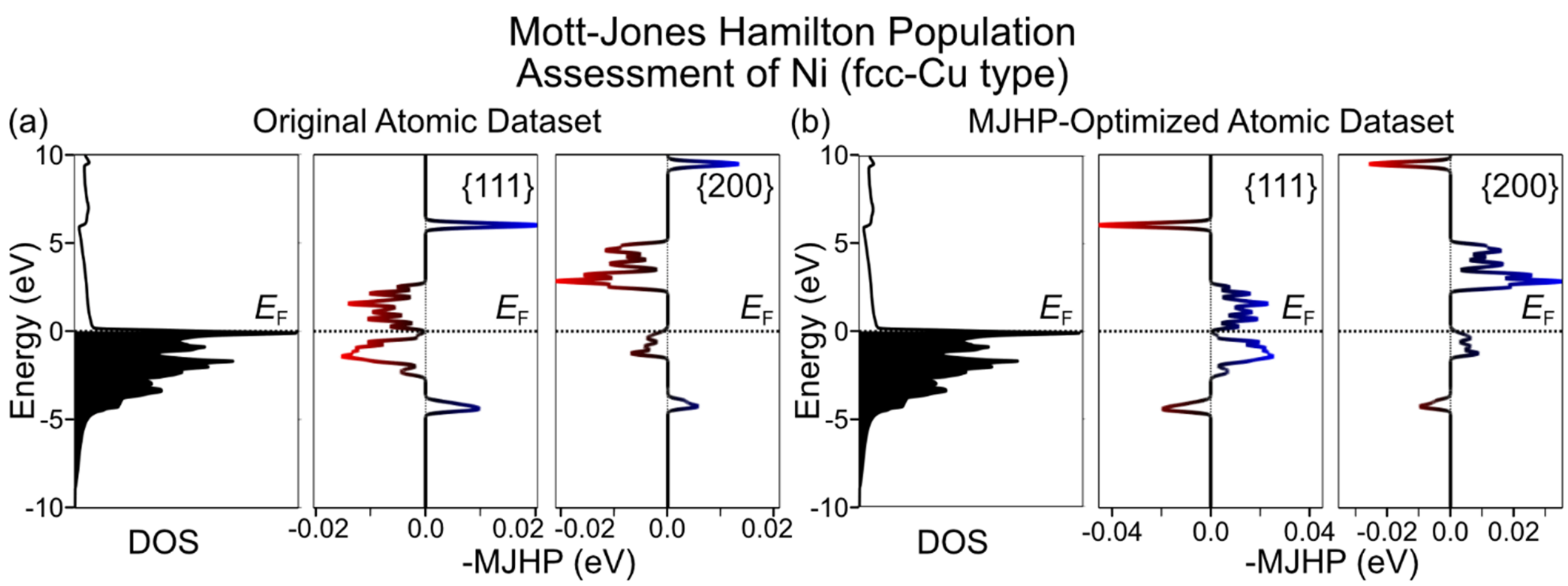

Figure S6. Comparison of the DOS distribution and $MJHP_{HKL}(E)$ curves for the {111} and {200} family of reciprocal lattice vectors in Ni (fcc-Cu type) for the (a) original and (b) optimized atomic dataset.

Cu (fcc-Cu type)

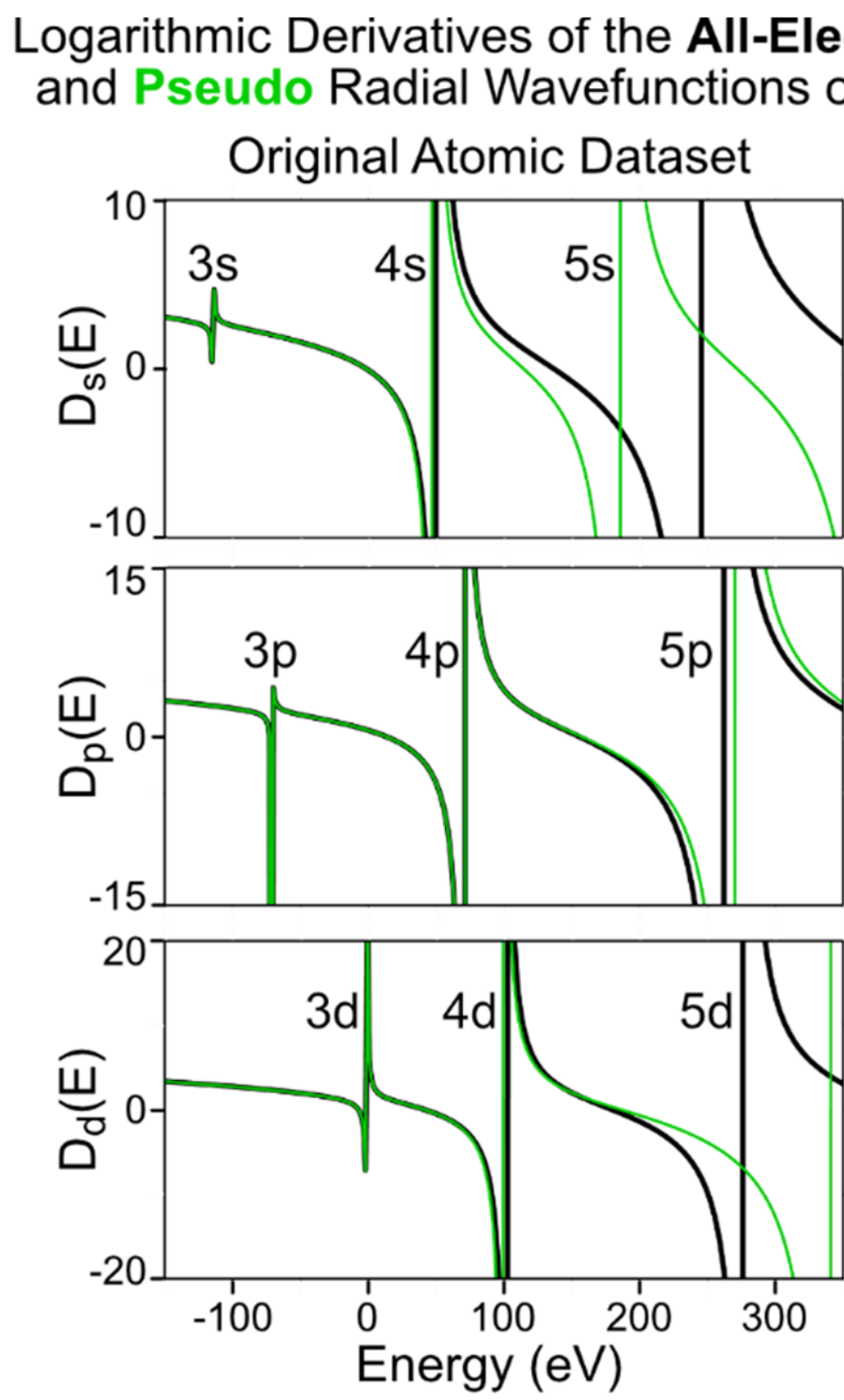

Figure S7. All-electron and pseudo radial wavefunction logarithmic derivative plots for Cu (atomic) calculated using the original atomic dataset.

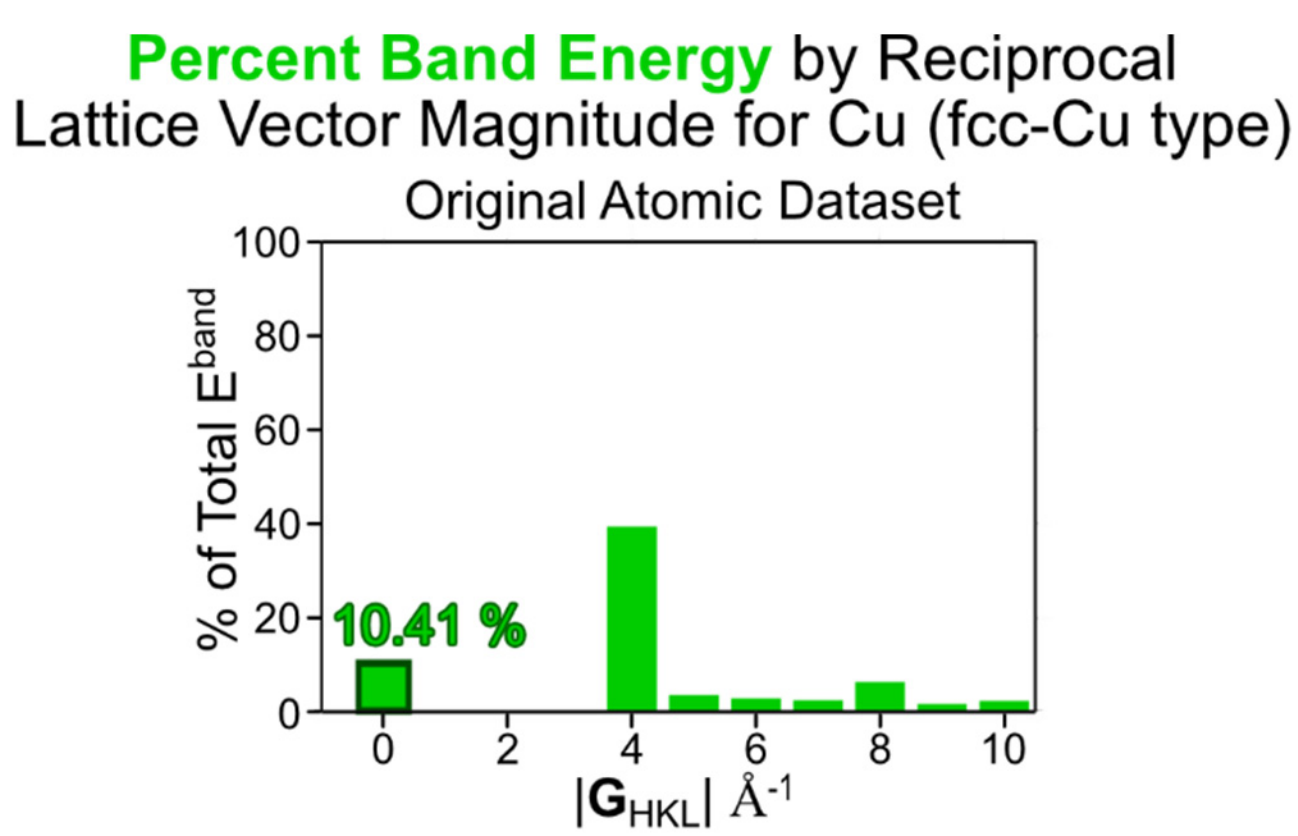

Figure S8. The distribution of the percent of the band energy by the reciprocal lattice vector magnitude for Cu (fcc-Cu type) calculated using the original atomic dataset, truncated after $|\mathbf{G}_{HKL}|$ exceeds 10 Å^{-1}.

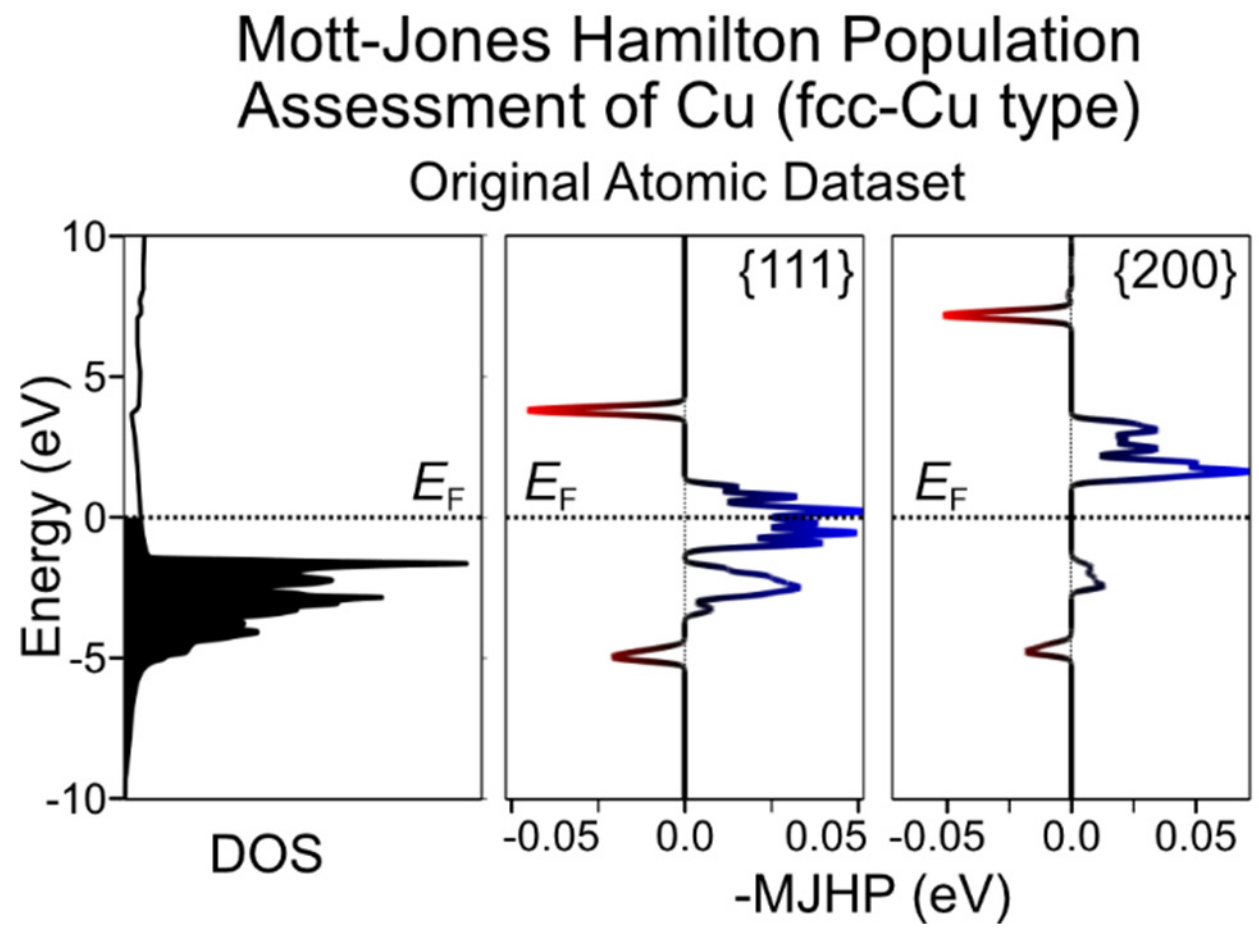

Figure S9. Comparison of the DOS distribution and $MJHP_{HKL}(E)$ curves for the {111} and {200} family of reciprocal lattice vectors in Cu (fcc-Cu type) for the (a) original and (b) MJHP optimized atomic dataset.

Zn (hcp-Mg type)

The logarithmic derivatives of the all-electron and pseudo radial wavefunctions for atomic Zn are found in the main text.

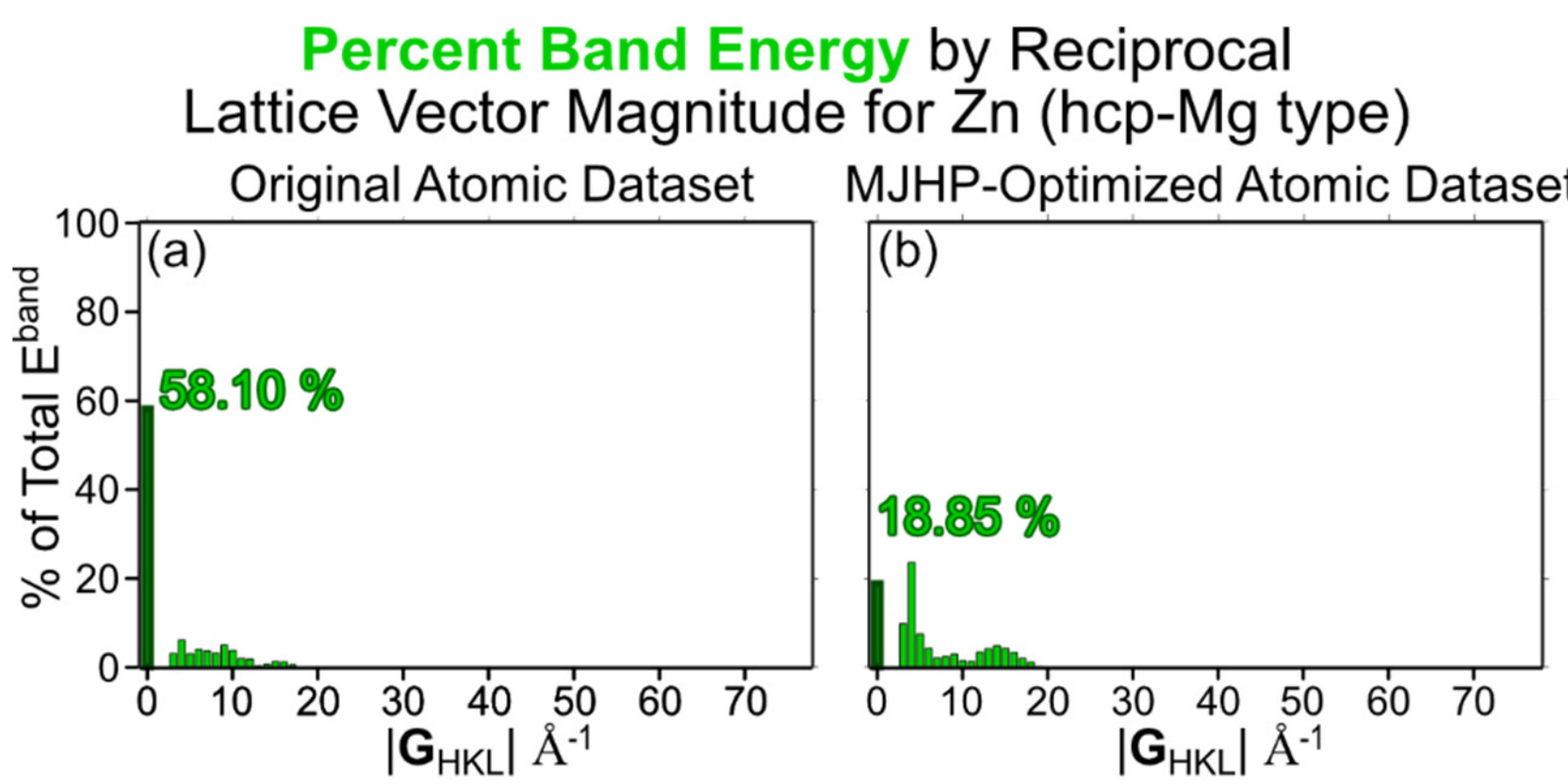

Figure S10. Comparison of the distribution of the percent of the band energy by the reciprocal lattice vector magnitude for Zn (hcp-Mg type) calculated using the (a) original and (b) optimized atomic dataset without truncation of $|\mathbf{G}_{HKL}|$.

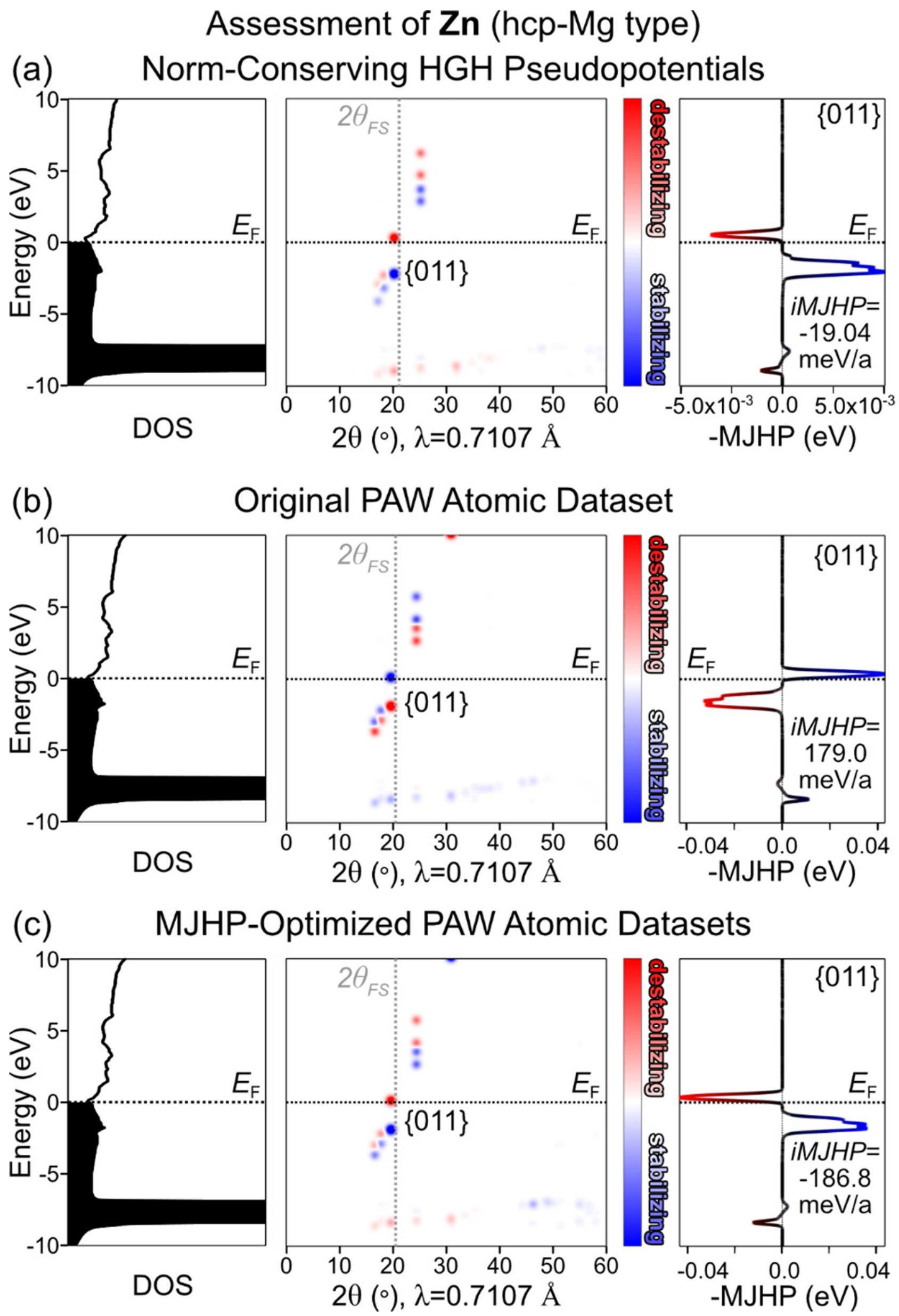

Figure S11. Comparison of the DOS distribution, $MJHP(E, 2\theta)$ plot, and $MJHP_{HKL}(E)$ curve for the {011} family of reciprocal lattice vectors in Zn (hcp-Mg type) for the (a) HGH norm-conserving pseudopotential method, (b) the PAW method with the original atomic dataset, and (c) the PAW method with the MJHP-optimized atomic dataset.

Ga (α-Ga type)

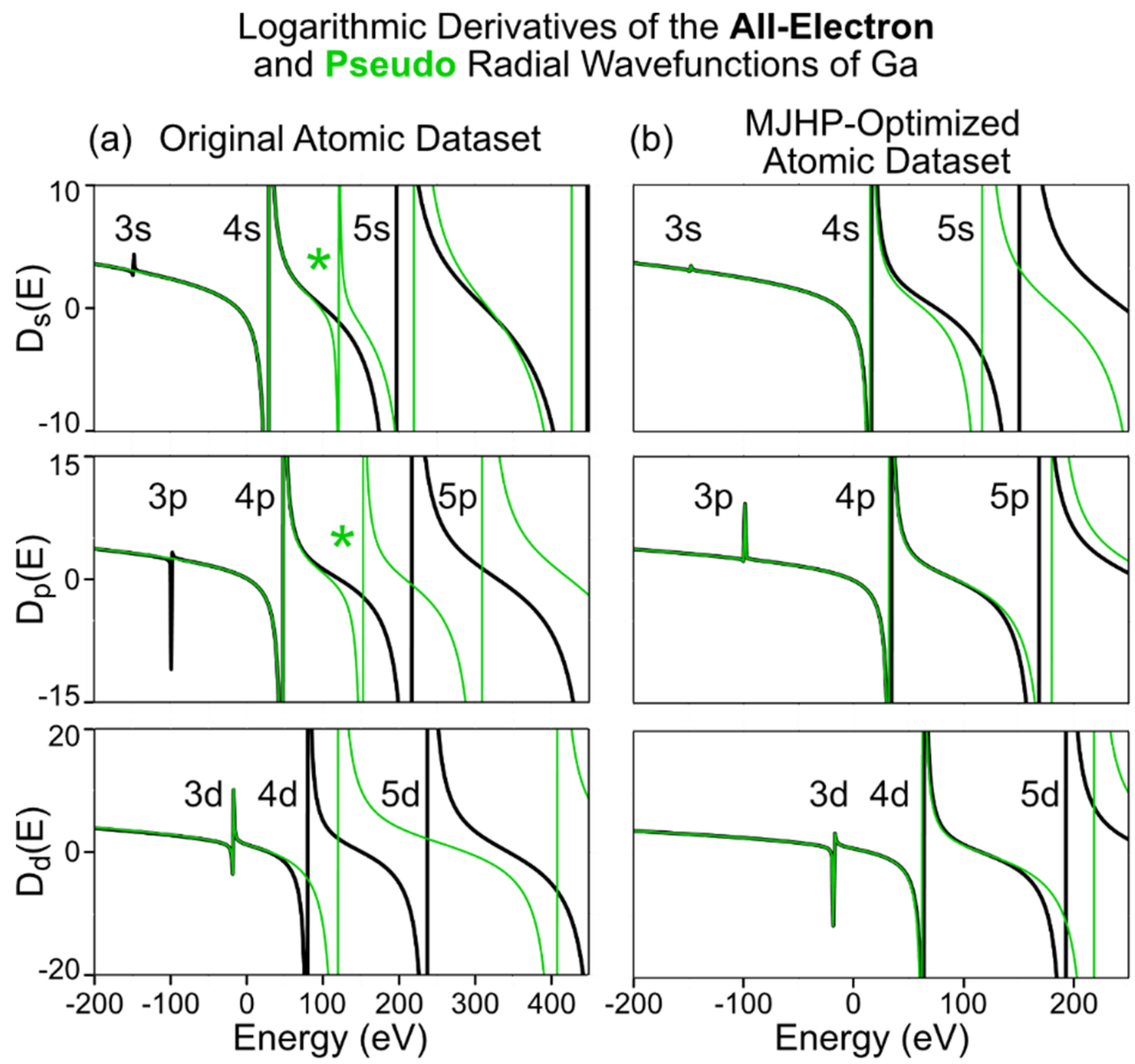

Figure S12. All-electron and pseudo-radial wavefunction logarithmic derivative plots for Ga (atomic) calculated using the (a) original and (b) optimized atomic dataset.

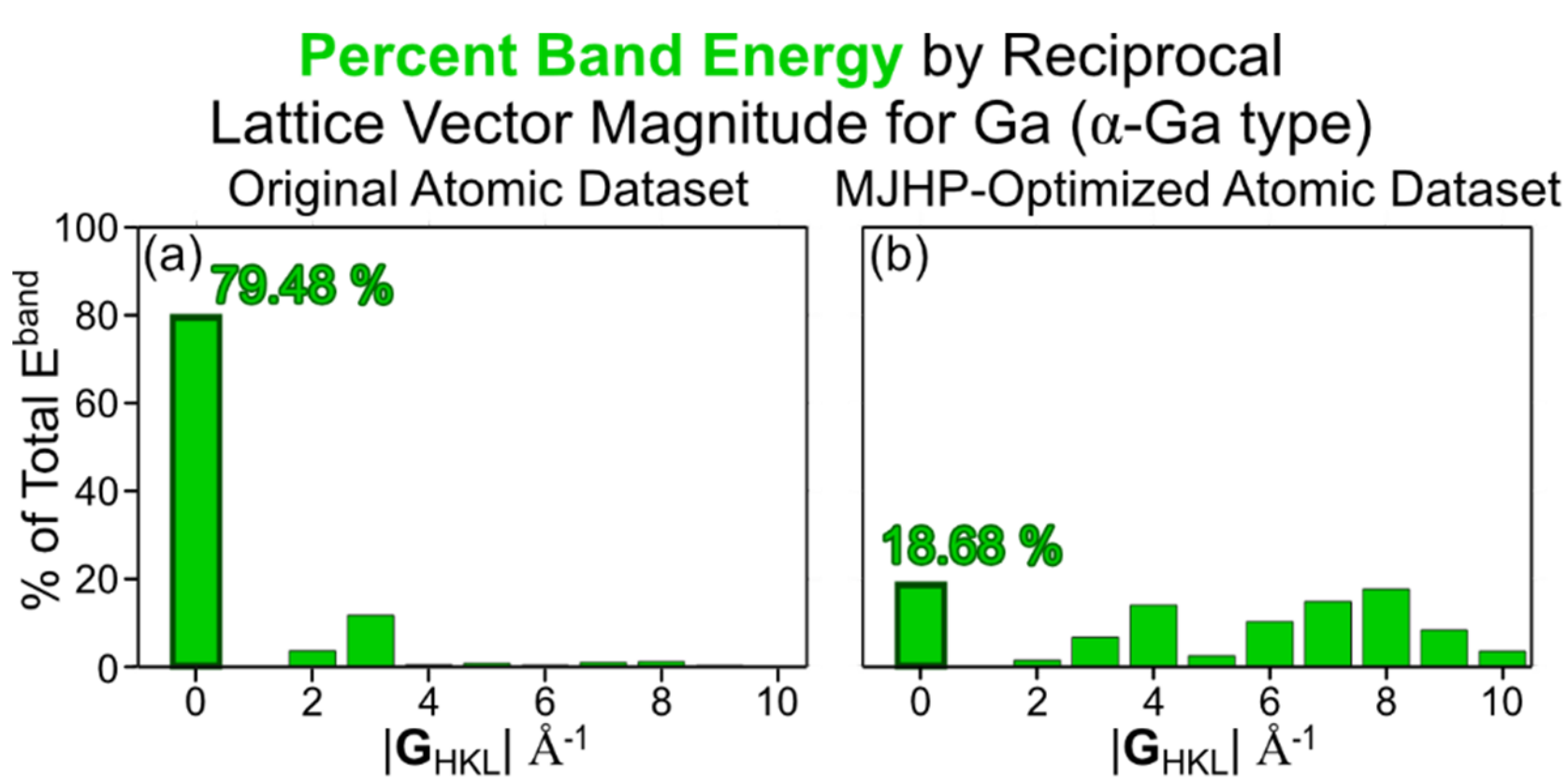

Figure S13. Comparison of the distribution of the percent of the band energy by the reciprocal lattice vector magnitude for Ga (α-Ga type) calculated using the (a) original and (b) optimized atomic dataset, truncated after $|\mathbf{G}_{HKL}|$ exceeds 10 Å^{-1}.

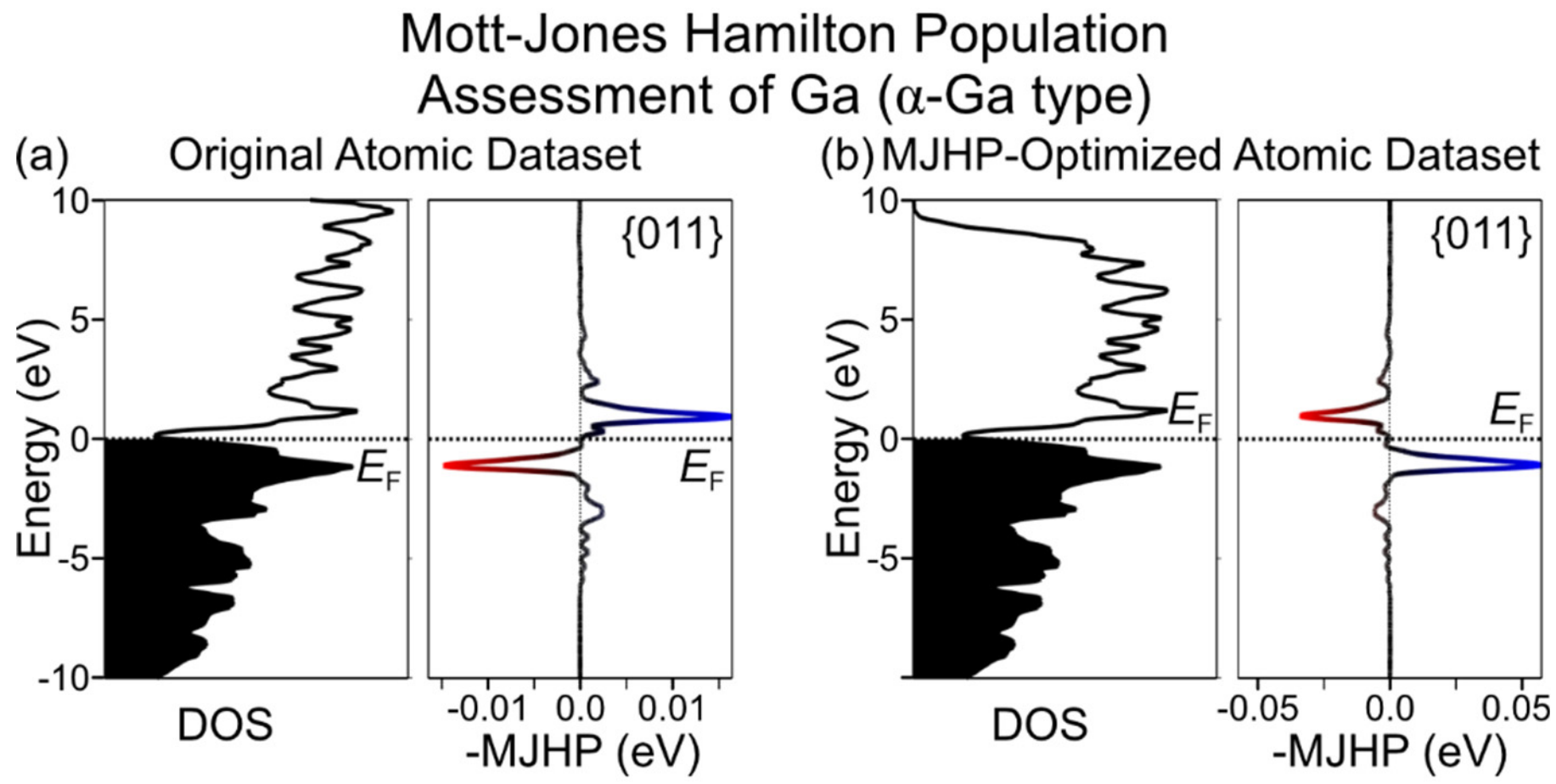

Figure S14. Comparison of the DOS distribution and $MJHP_{HKL}(E)$ curve for the {011} family of reciprocal lattice vectors in Ga (α-Ga type) for the (a) original and (b) optimized atomic dataset.

Pd (fcc-Cu type)

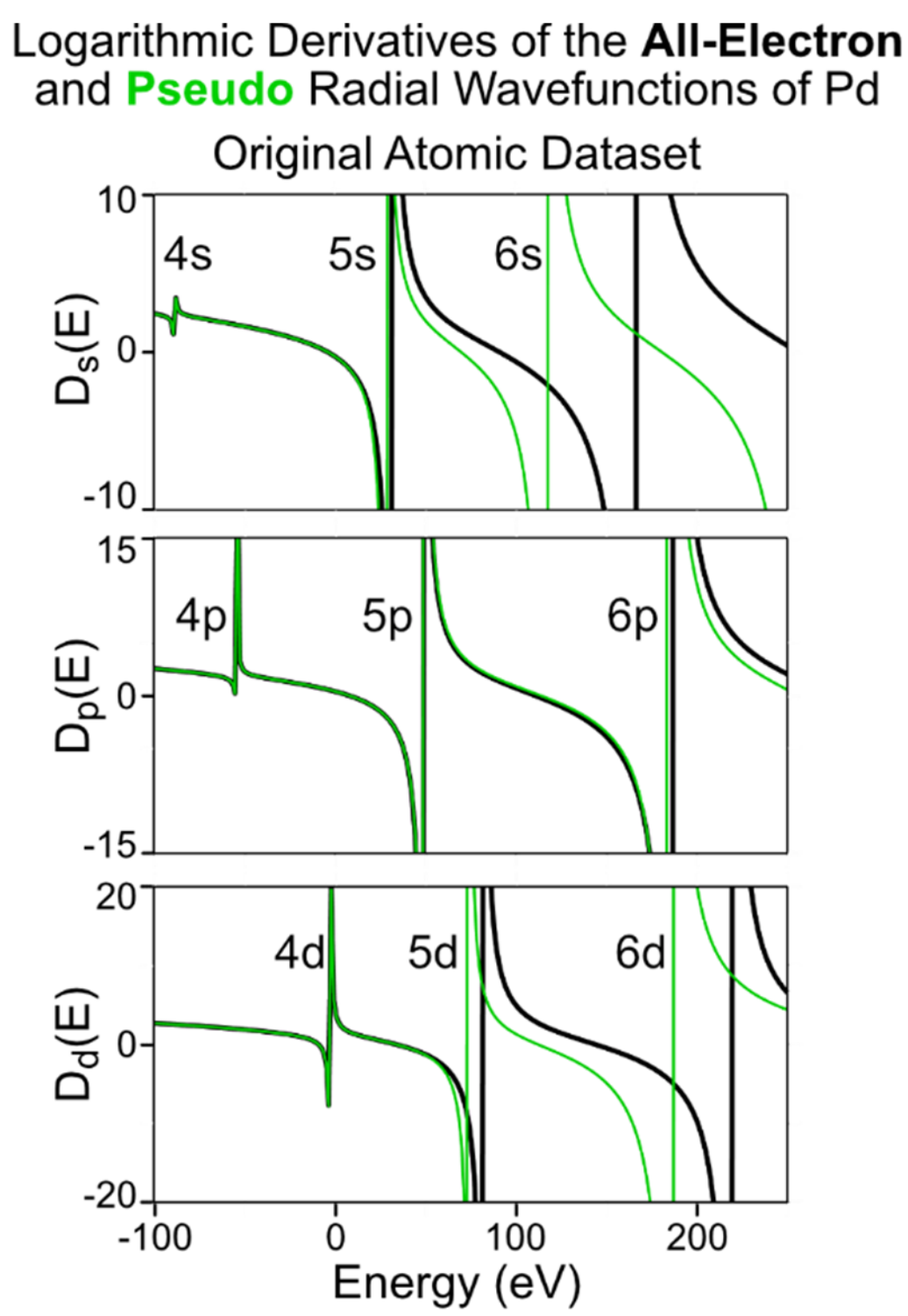

Figure S15. All-electron and pseudo-radial wavefunction logarithmic derivative plots of Pd (atomic) calculated using the original atomic dataset.

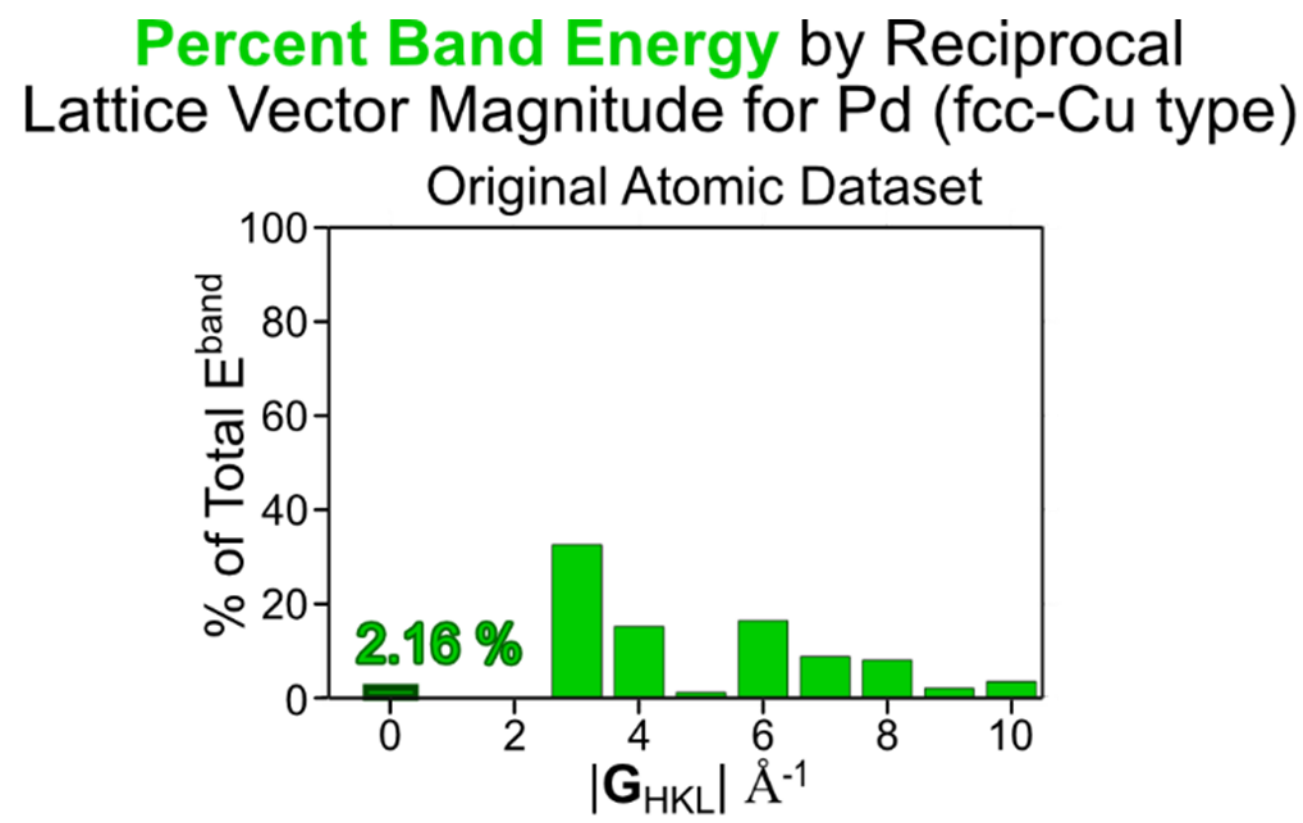

Figure S16. Comparison of the distribution of the percent of the band energy by the reciprocal lattice vector magnitude for Pd (fcc-Cu type) calculated using the original atomic dataset, truncated after $|\mathbf{G}_{HKL}|$ exceeds 10 Å^{-1}.

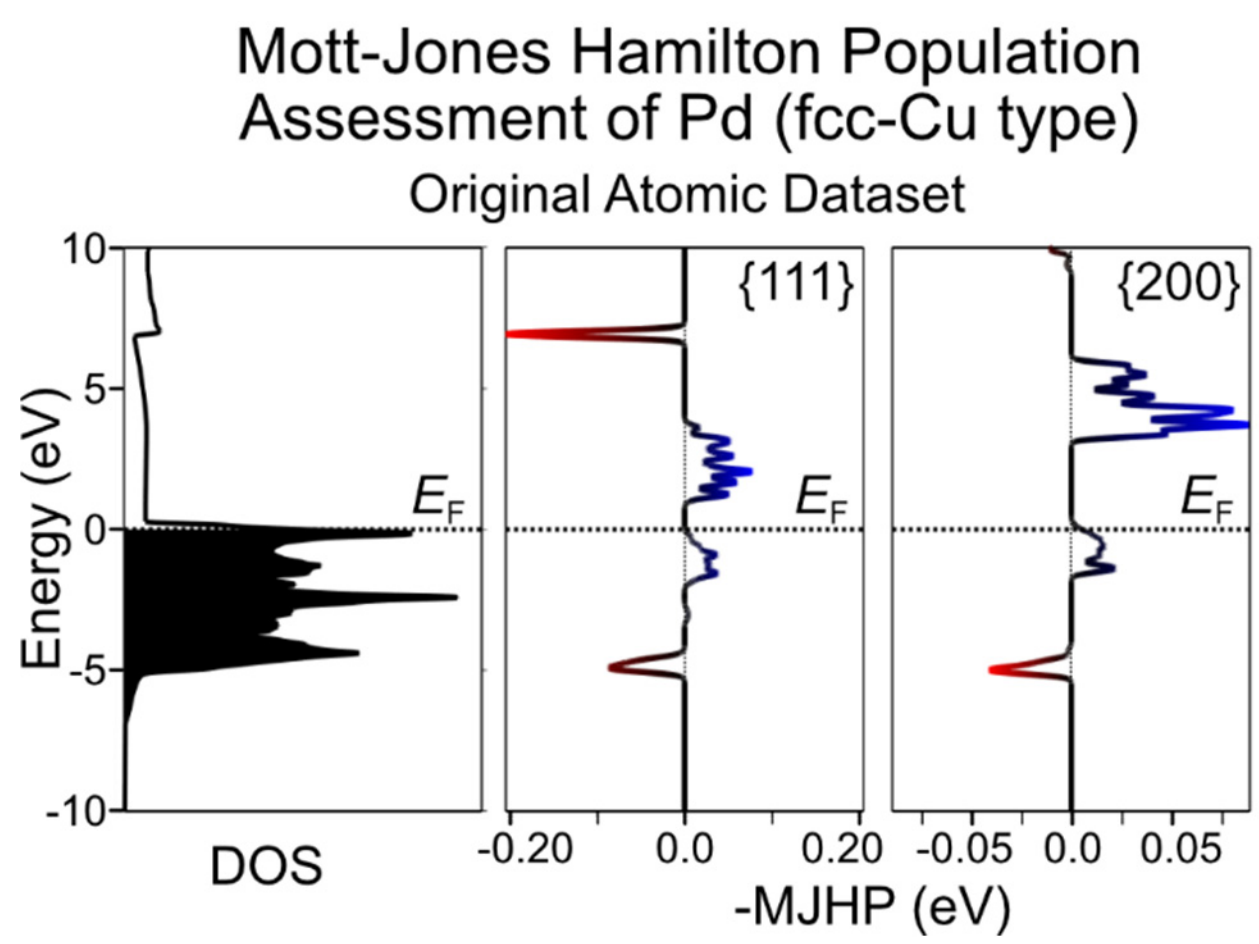

Figure S17. Comparison of the DOS distribution and $MJHP_{HKL}(E)$ curves for the {111} and {200} family of reciprocal lattice vectors in Pd (fcc-Cu type) for the original atomic dataset.

Ag (fcc-Cu type)

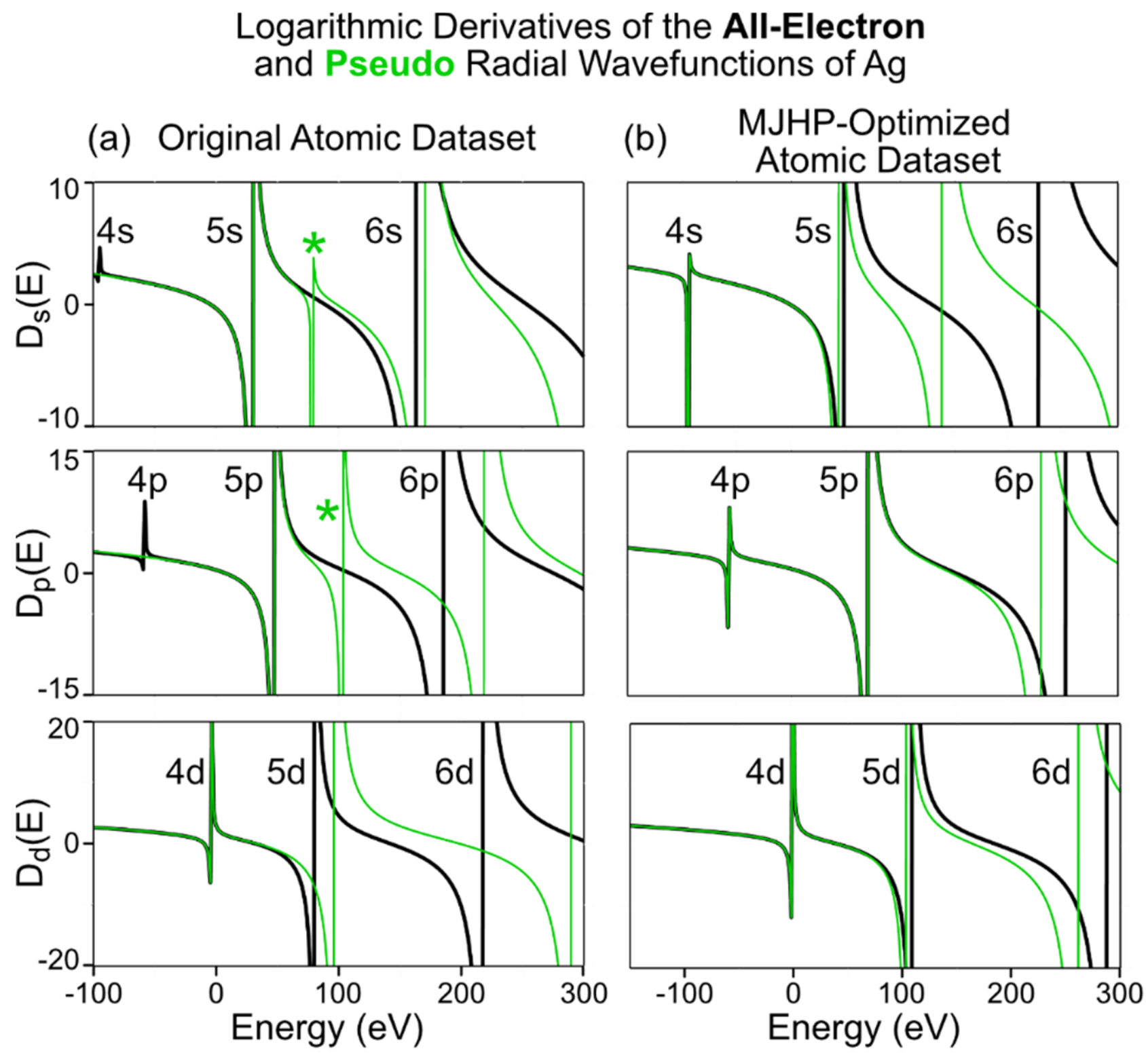

Figure S18. All-electron and pseudo radial wavefunction logarithmic derivative plots for Ag (atomic) calculated using the (a) original and (b) optimized atomic dataset.

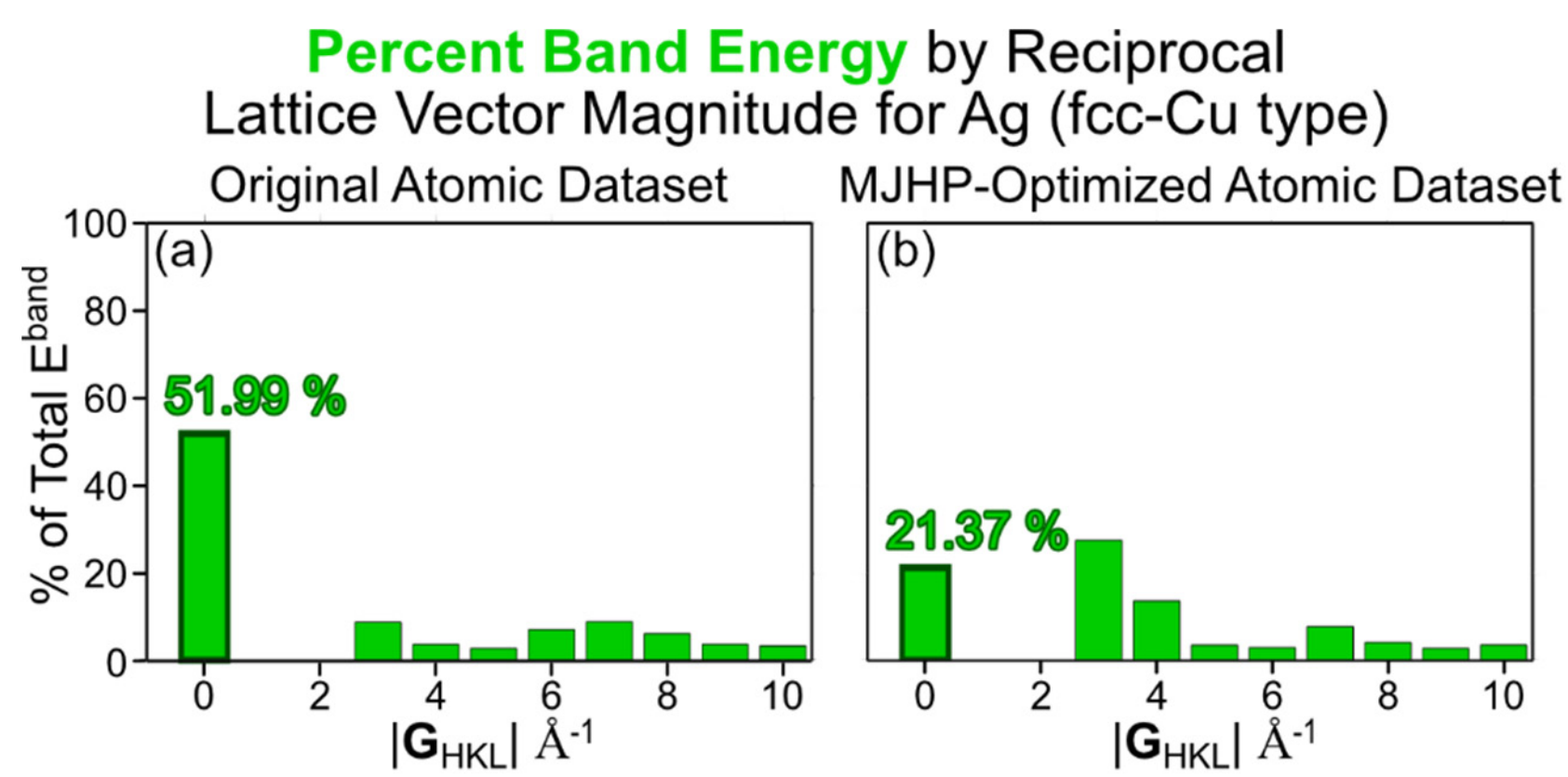

Figure S19. Comparison of the distribution of the percent of the band energy by the reciprocal lattice vector magnitude for Ag (fcc-Cu type) calculated using the (a) original and (b) optimized atomic dataset, truncated after $|\mathbf{G}_{HKL}|$exceeds 10 Å^{-1}.

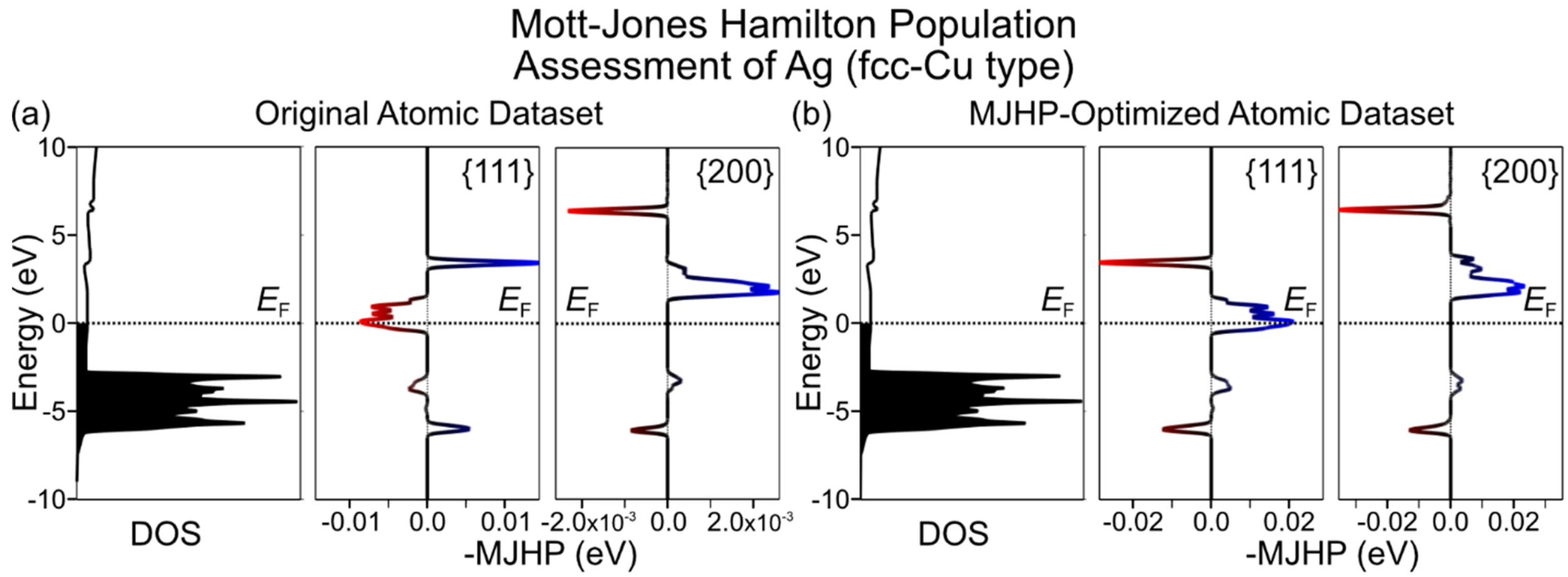

Figure S20. Comparison of the DOS distribution and $MJHP_{HKL}(E)$ curves for the {111} and {200} family of reciprocal lattice vectors in Ag (fcc-Cu type) for the (a) original and (b) optimized atomic dataset.

Cd (hcp-Mg type)

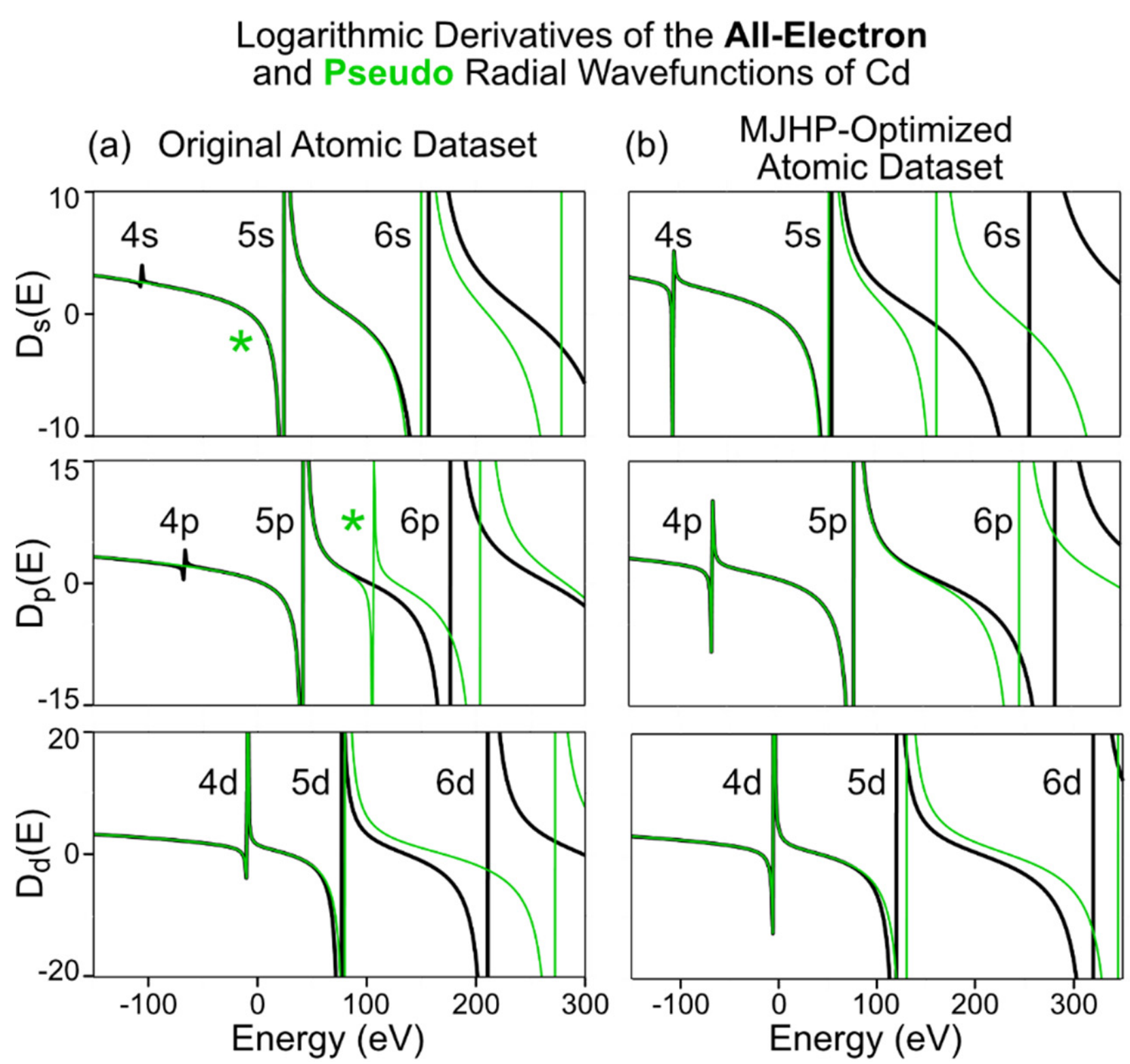

Figure S21. All-electron and pseudo radial wavefunction logarithmic derivative plots for Cd (atomic) calculated using the (a) original and (b) optimized atomic dataset.

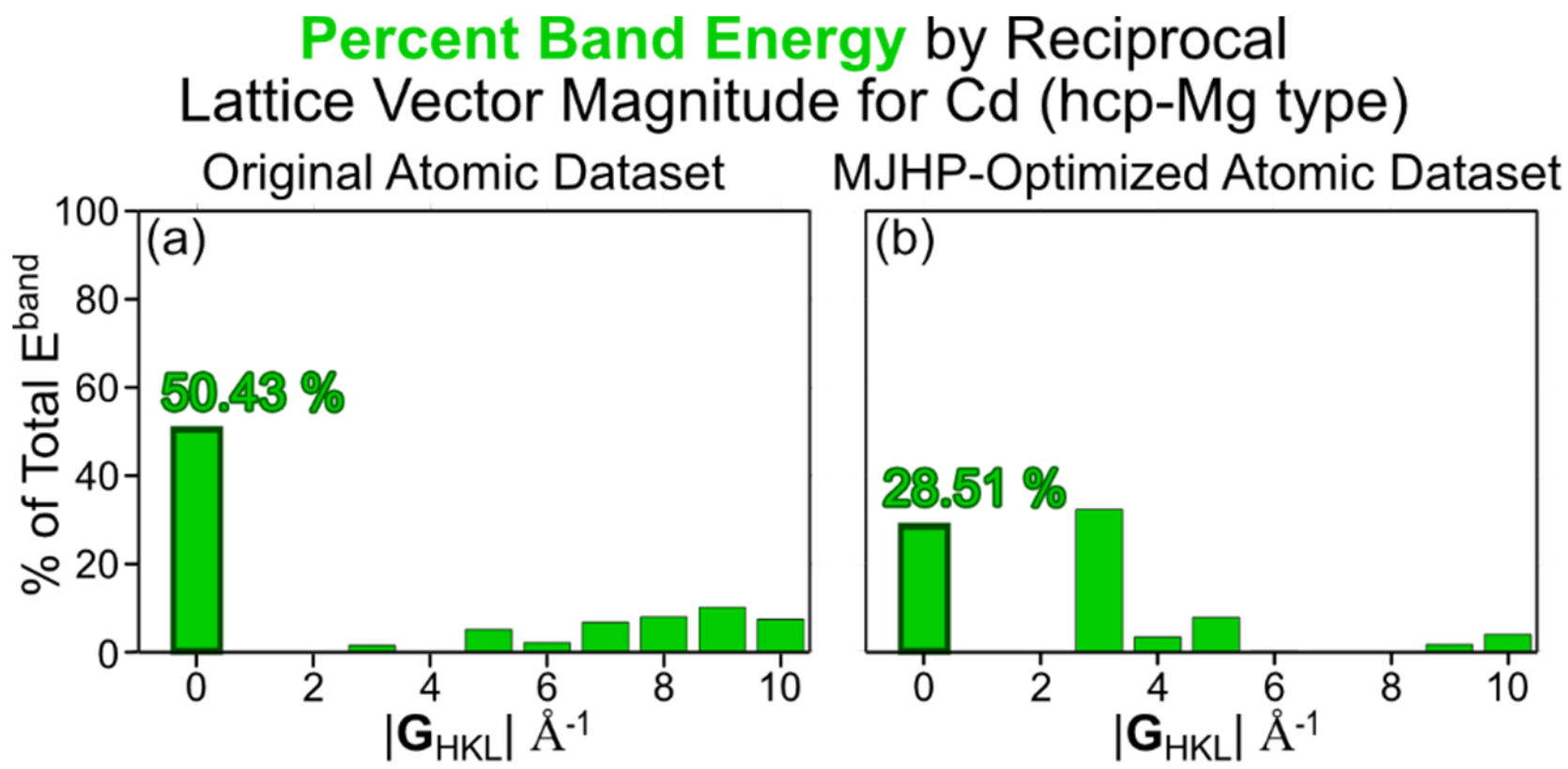

Figure S22. Comparison of the distribution of the percent of the band energy by the reciprocal lattice vector magnitude for Cd (hcp-Mg type) calculated using the (a) original and (b) optimized atomic dataset, truncated after $|\mathbf{G}_{HKL}|$ exceeds 10 Å^{-1}.

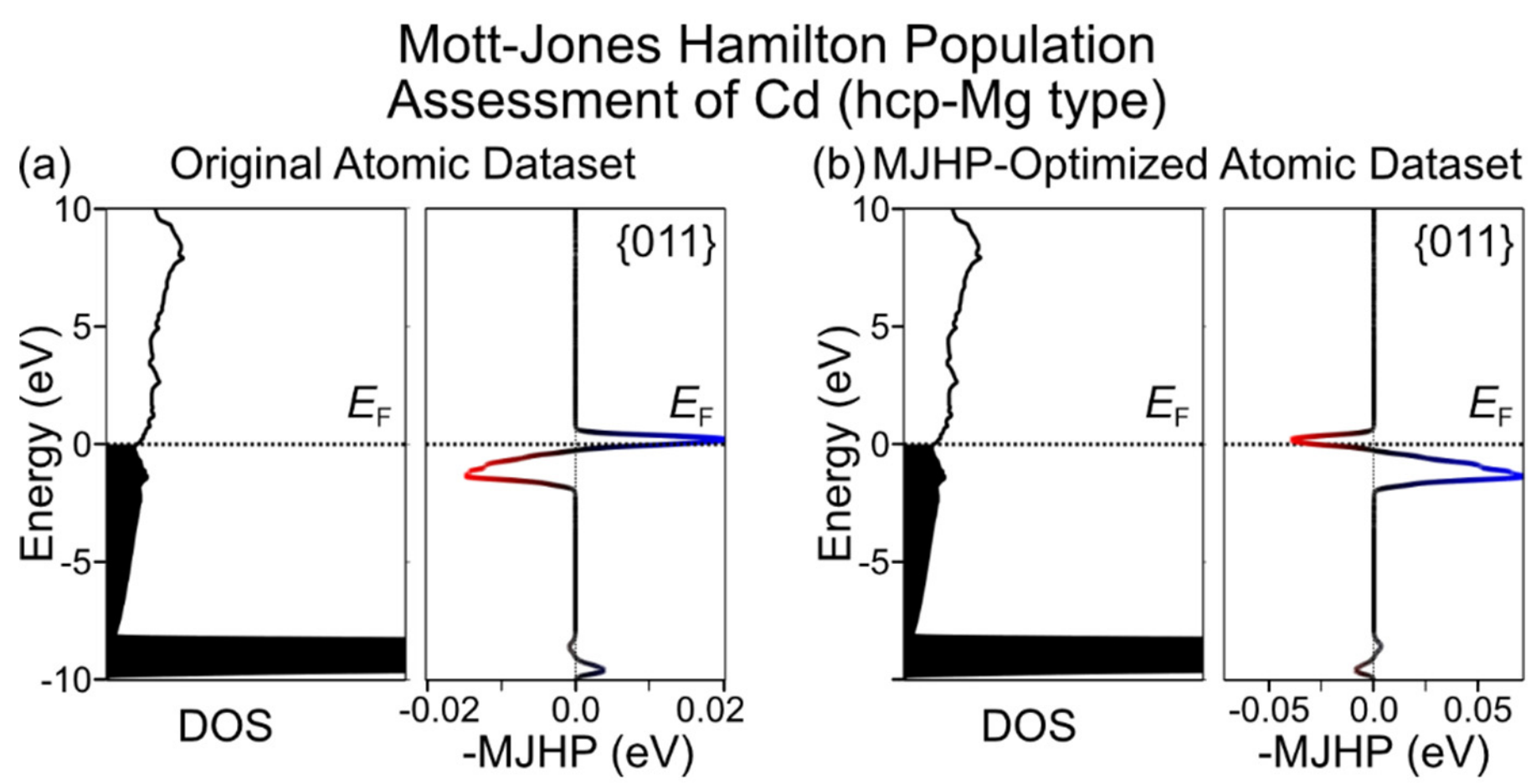

Figure S23. Comparison of the DOS distribution and $MJHP_{HKL}(E)$ curve for the {011} family of reciprocal lattice vectors in Cd (hcp-Mg type) for the (a) original and (b) optimized atomic dataset.

Pt (fcc-Cu type)

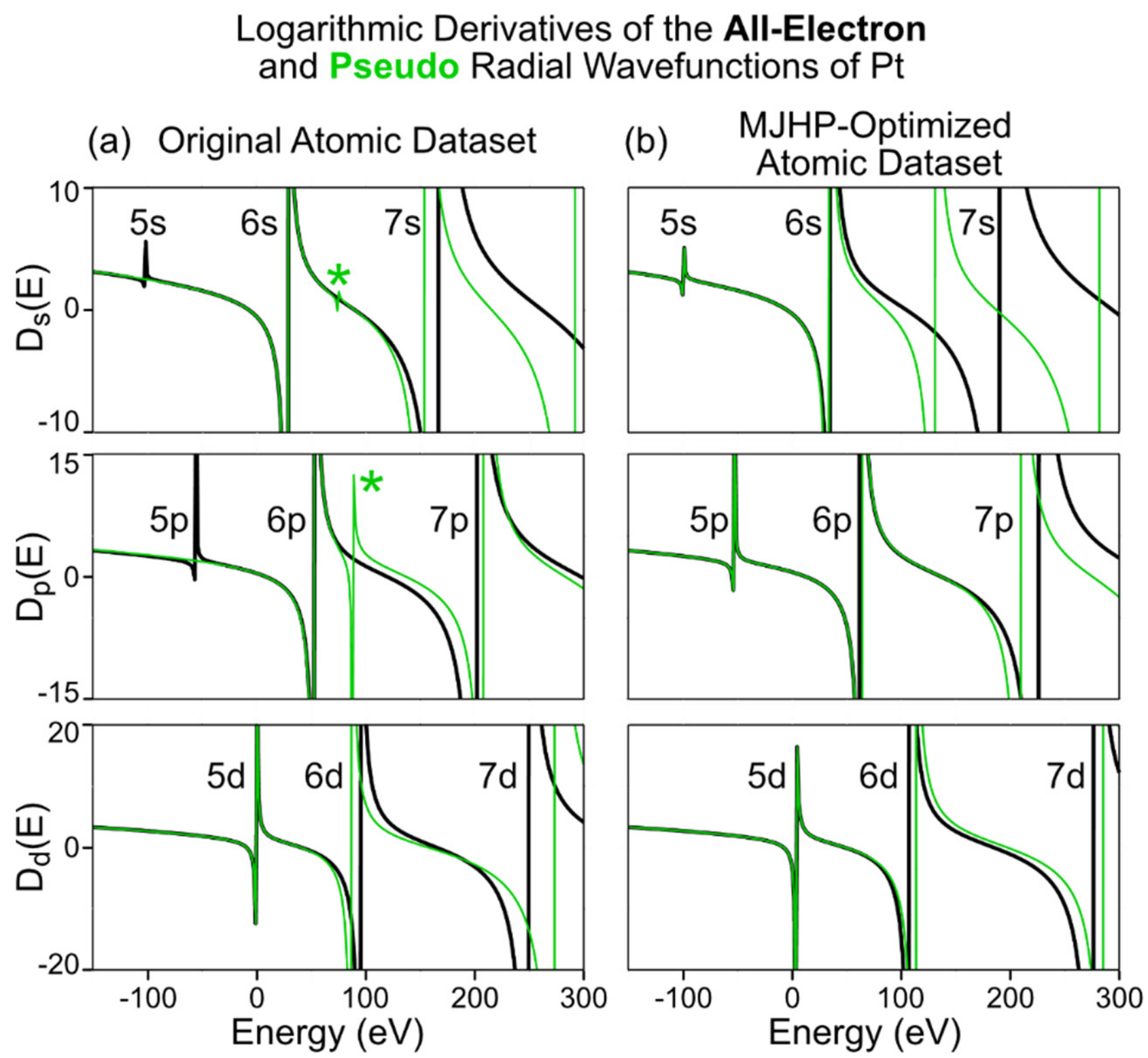

Figure S24. All-electron and pseudo radial wavefunction logarithmic derivative plots for Pt (atomic) calculated using the (a) original and (b) optimized atomic dataset.

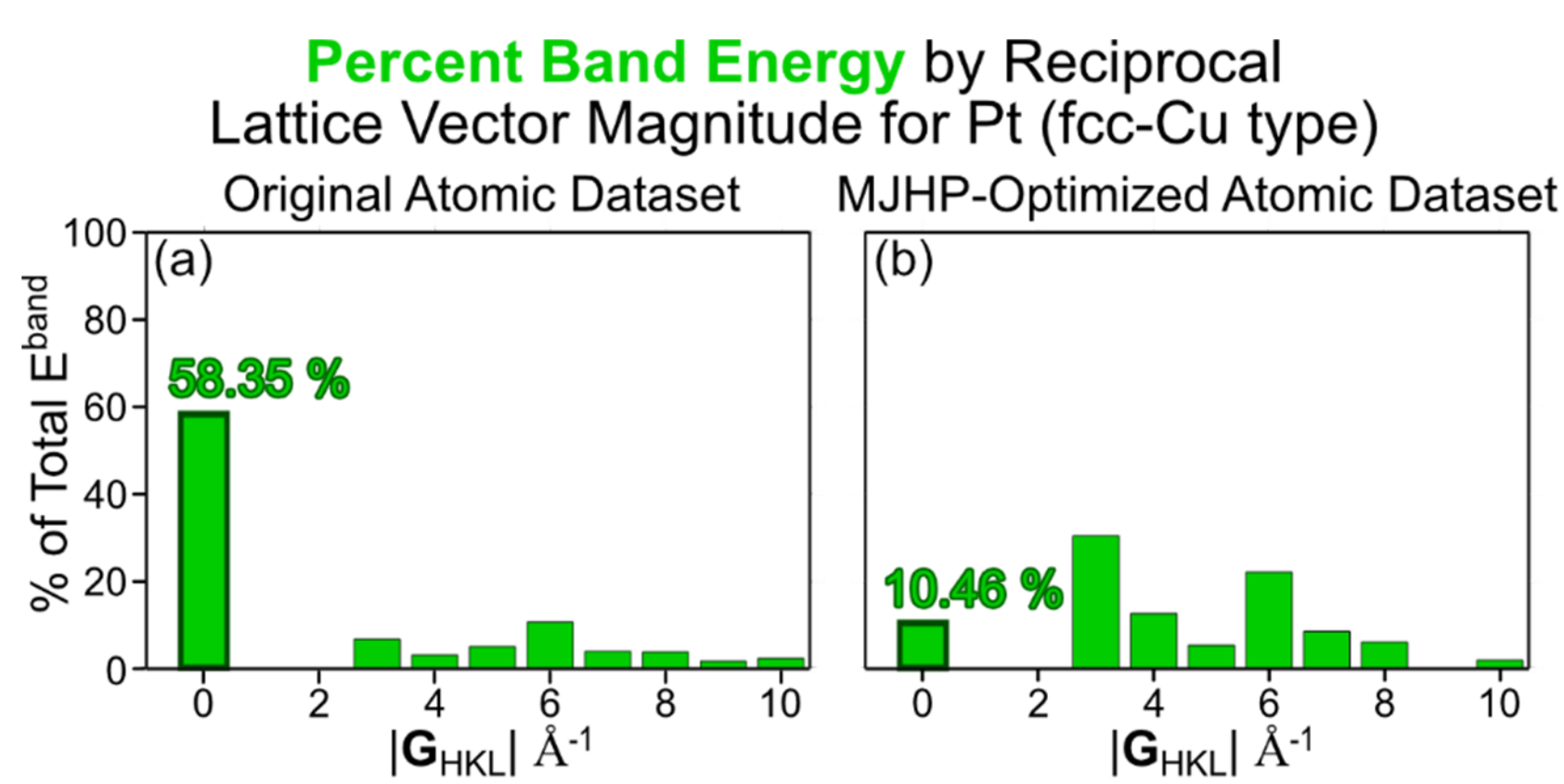

Figure S25. Comparison of the distribution of the percent of the band energy by the reciprocal lattice vector magnitude for Pt (fcc-Cu type) calculated using the (a) original and (b) optimized atomic dataset, truncated after $|\mathbf{G}_{HKL}|$ exceeds 10 Å^{-1}.

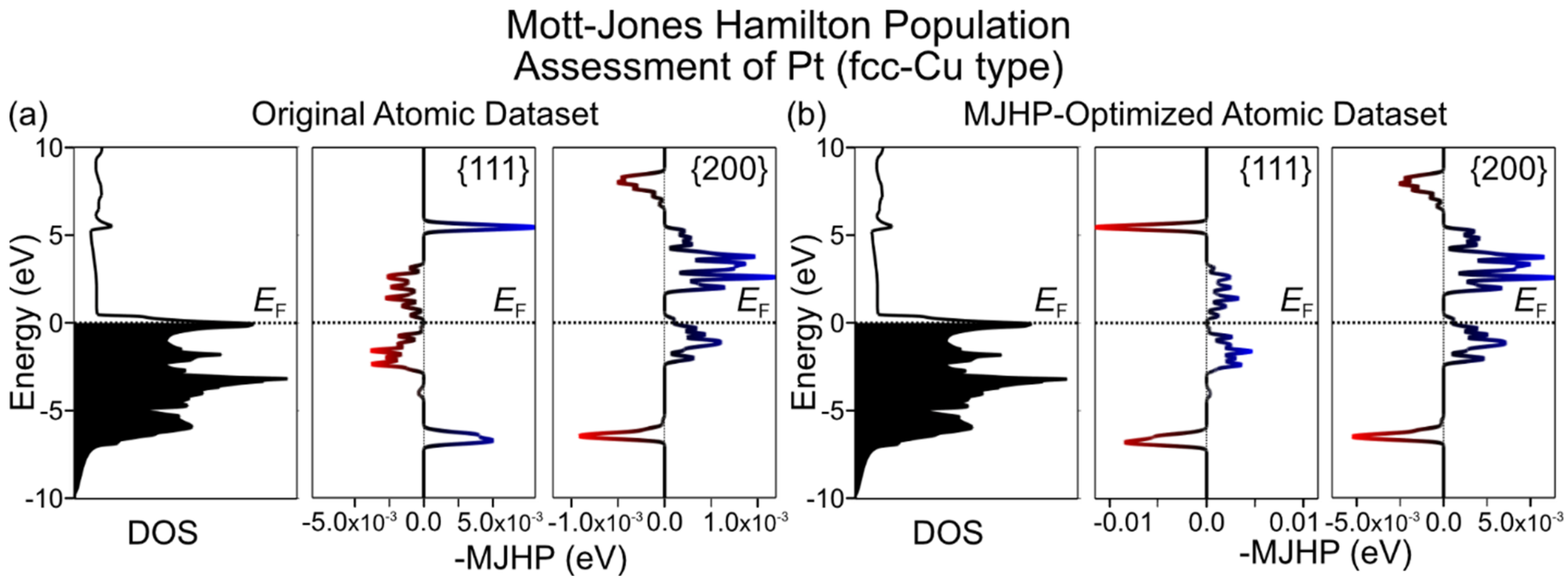

Figure S26. Comparison of the DOS distribution and $MJHP_{HKL}(E)$ curves for the {111} and {200} family of reciprocal lattice vectors in Pt (fcc-Cu type) for the (a) original and (b) optimized atomic dataset.

Au (fcc-Cu type)

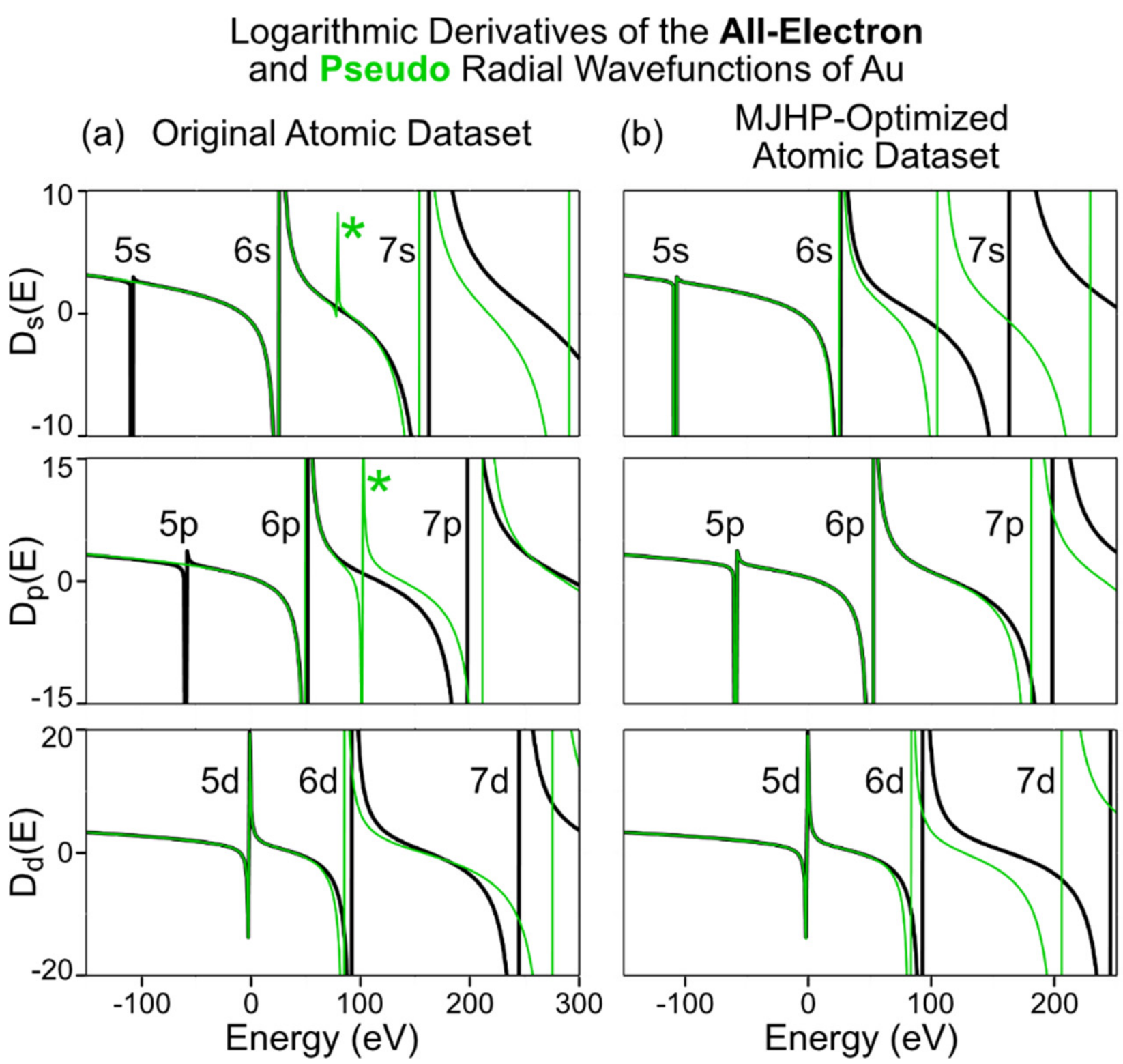

Figure S27. All-electron and pseudo radial wavefunction logarithmic derivative plots for Au (atomic) calculated using the (a) original and (b) optimized atomic dataset.

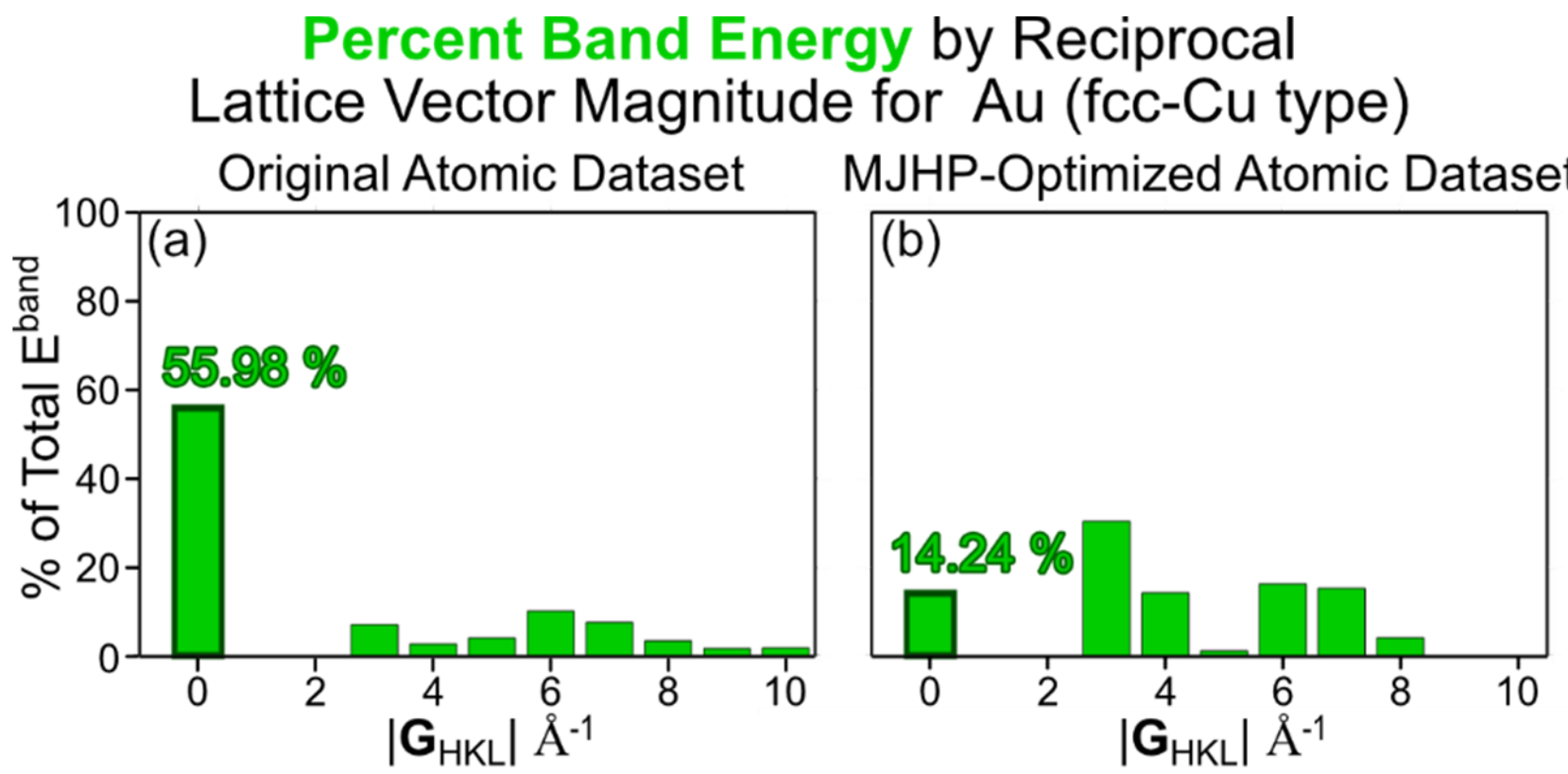

Figure S28. Comparison of the distribution of the percent of the band energy by the reciprocal lattice vector magnitude for Au (fcc-Cu type) calculated using the (a) original and (b) optimized atomic dataset, truncated after $|\mathbf{G}_{HKL}|$ exceeds 10 Å^{-1}.

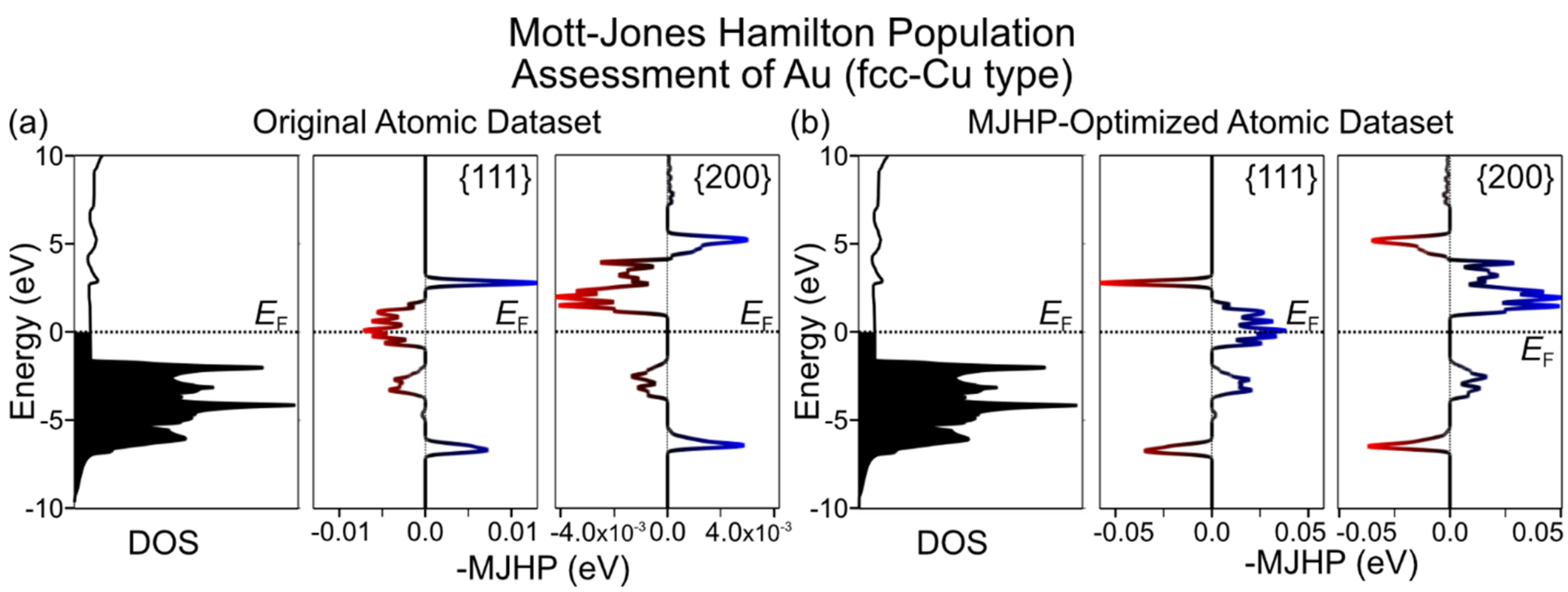

Figure S29. Comparison of the DOS distribution and $MJHP_{HKL}(E)$ curves for the {111} and {200} family of reciprocal lattice vectors in Au (fcc-Cu type) for the (a) original and (b) optimized atomic dataset.

S5. Computational Details and Optimized Geometries for the Binary Compounds

Table S30. Computational details for the geometrical optimizations using ABINIT.

Structure	Calc. Type	Coarse E Cutoff (Ha)	Fine E Cutoff (Ha)	*k*-point Mesh	No. *k*-points	Coarse FFT Grid	Fine FFT Grid	Total E/Cell (Ha)
CuZn (CsCl-type)	HGH-NC	115	-	12×12×12	84	64×64×64	-	-108.5766
CuZn (CsCl-type)	original	22	26	12×12×12	84	30×30×30	30×30×30	-262.2826
CuZn (CsCl-type)	optimized	24	30	12×12×12	84	30×30×30	32×32×32	-425.6926
AgCd (CsCl-type)	HGH-NC	50	-	12×12×12	84	48×48×48	-	-83.0591
AgCd (CsCl-type)	original	16	32	12×12×12	84	30×30×30	40×40×40	-85.7771
AgCd (CsCl-type)	optimized	20	24	12×12×12	84	30×30×30	32×32×32	-316.7733
$AlNi_3$ ($AuCu_3$-type)	HGH-NC	95	-	8×8×8	35	60×60×60	-	-113.9393
$AlNi_3$ ($AuCu_3$-type)	original	38	42	8×8×8	35	40×40×40	40×40×40	-514.7755
$AlNi_3$ ($AuCu_3$-type)	optimized	26	38	8×8×8	35	32×32×32	40×40×40	-594.2764

Table S31. Computational details for ABINIT calculations used for the MJHP and DOS distribution analysis.

Structure	Calc. Type	Coarse E Cutoff (Ha)	Fine E Cutoff (Ha)	*k*-point Mesh	No. *k*-points	Coarse FFT Grid	Fine FFT Grid	Total E/Cell (Ha)
CuZn (CsCl-type)	HGH-NC	115	-	48×48×48	2925	54×54×54	-	-108.5768
CuZn (CsCl-type)	original	22	26	48×48×48	2925	24×24×24	27×27×27	-262.2827
CuZn (CsCl-type)	optimized	24	30	48×48×48	2925	27×27×27	30×30×30	-425.6927
AgCd (CsCl-type)	HGH-NC	50	-	48×48×48	2925	40×40×40	-	-83.0592
AgCd (CsCl-type)	original	16	32	48×48×48	2925	24×24×24	36×36×36	-85.7772
AgCd (CsCl-type)	optimized	20	24	48×48×48	2925	27×27×27	30×30×30	-316.7733
$AlNi_3$ ($AuCu_3$-type)	HGH-NC	95	-	32×32×32	969	60×60×60	-	-113.9394
$AlNi_3$ ($AuCu_3$-type)	original	38	42	32×32×32	969	40×40×40	40×40×40	-514.7755
$AlNi_3$ ($AuCu_3$-type)	optimized	26	38	32×32×32	969	32×32×32	40×40×40	-594.2765

Table S32. Optimized cell parameters from ABINIT.

Structure	Atomic Dataset	a (Å)	b (Å)	c (Å)	α	β	γ
CuZn (CsCl-type)	HGH-NC	2.8867	2.8867	2.8867	90	90	90
CuZn (CsCl-type)	original	2.9673	2.9673	2.9673	90	90	90
CuZn (CsCl-type)	optimized	2.9618	2.9618	2.9618	90	90	90
AgCd (CsCl-type)	HGH-NC	3.2787	3.2787	3.2787	90	90	90
AgCd (CsCl-type)	original	3.3865	3.3865	3.3865	90	90	90
AgCd (CsCl-type)	optimized	3.3853	3.3853	3.3853	90	90	90
$AlNi_3$ ($AuCu_3$-type)	HGH-NC	3.4810	3.4810	3.4810	90	90	90
$AlNi_3$ ($AuCu_3$-type)	original	3.5730	3.5730	3.5730	90	90	90
$AlNi_3$ ($AuCu_3$-type)	optimized	3.5737	3.5737	3.5737	90	90	90

Table S33. Fractional atomic coordinates for CuZn (CsCl-type).

Wyckoff	Atom	x	y	z
1a	Cu1	0	0	0
1b	Zn1	0.5	0.5	0.5

Table S34. Fractional atomic coordinates for AgCd (CsCl-type).

Wyckoff	Atom	x	y	z
1a	Ag1	0	0	0
1b	Cd1	0.5	0.5	0.5

Table S35. Fractional atomic coordinates for $AlNi_3$ ($AuCu_3$-type).

Wyckoff	Atom	x	y	z
1a	Al1	0	0	0
3c	Ni1	0	0.5	0.5
	Ni2	0.5	0	0.5
	Ni3	0.5	0.5	0

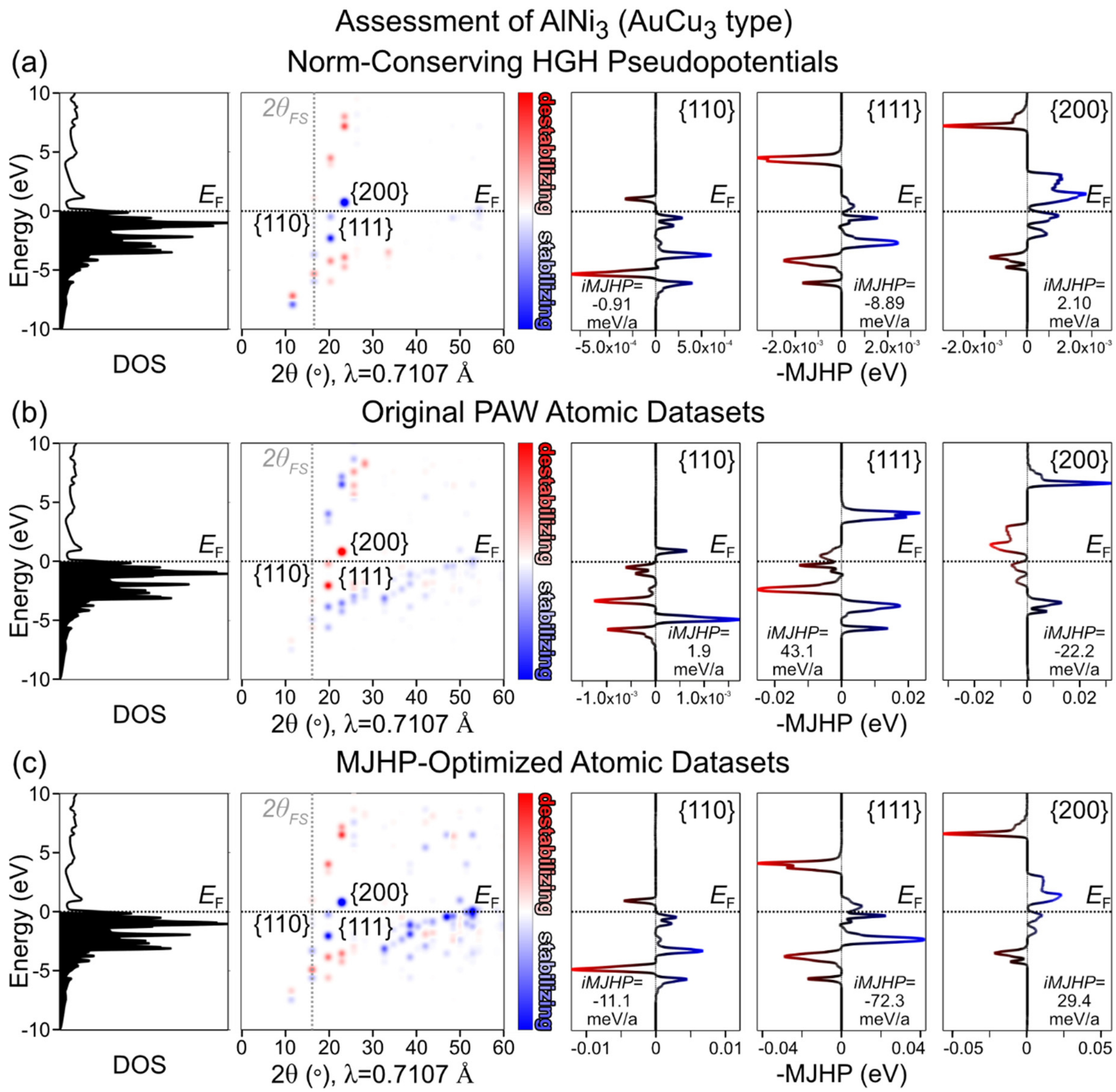

Figure S30. Comparison of the DOS distribution, $MJHP(E, 2\theta)$ plot, and $MJHP_{HKL}(E)$ curves for the $\{110\}$, $\{111\}$, and $\{200\}$ family of reciprocal lattice vectors in $AlNi_3$ ($AuCu_3$-type) for the (a) HGH norm-conserving pseudopotential DFT method, (b) the PAW method using the original atomic datasets, and (c) the MJHP-optimized atomic datasets within the PAW method.

S6. Dependence of MJHP Results for CuZn on the Δ_{shell} and σ Parameters.

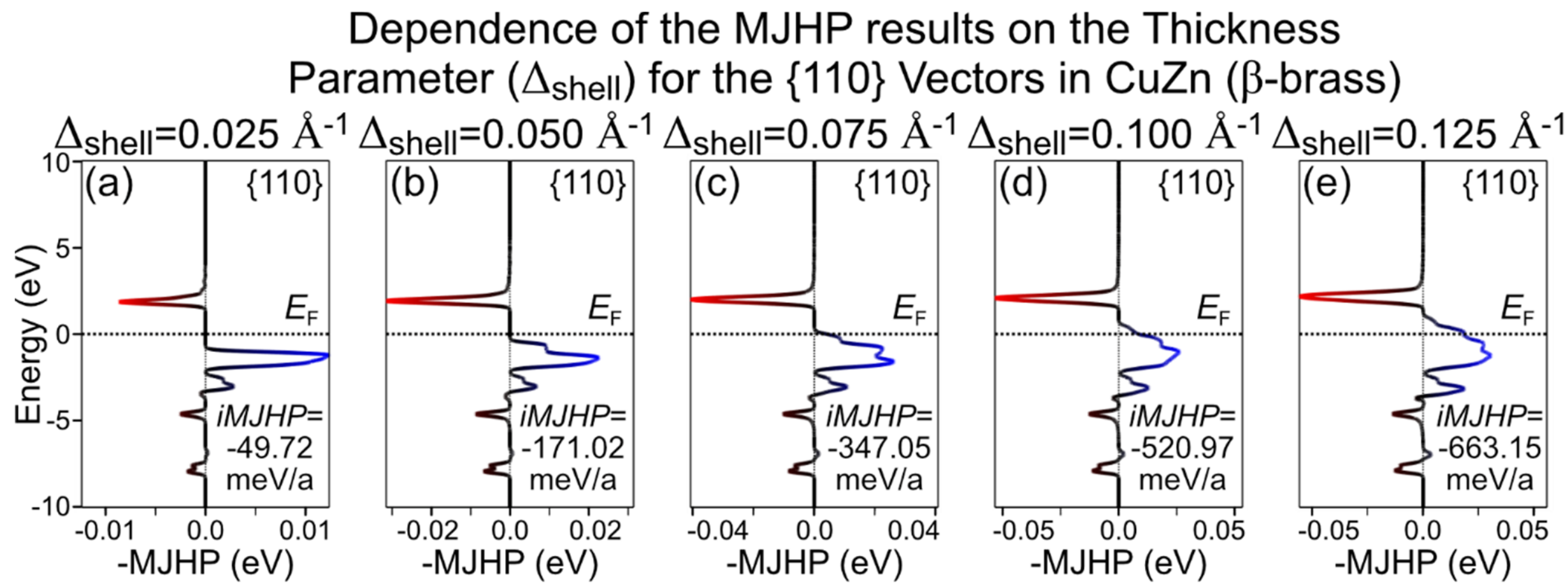

Figure S31. $MJHP_{HKL}(E)$ curves for the {110} family of reciprocal lattice vectors for β-brass, CuZn with varying thickness (Δ_{shell}) parameter values: (a) 0.025 Å^{-1}, (b) 0.050 Å^{-1}, (c) 0.075 Å^{-1}, (d) 0.100 Å^{-1}, and (e) 0.125 Å^{-1}.

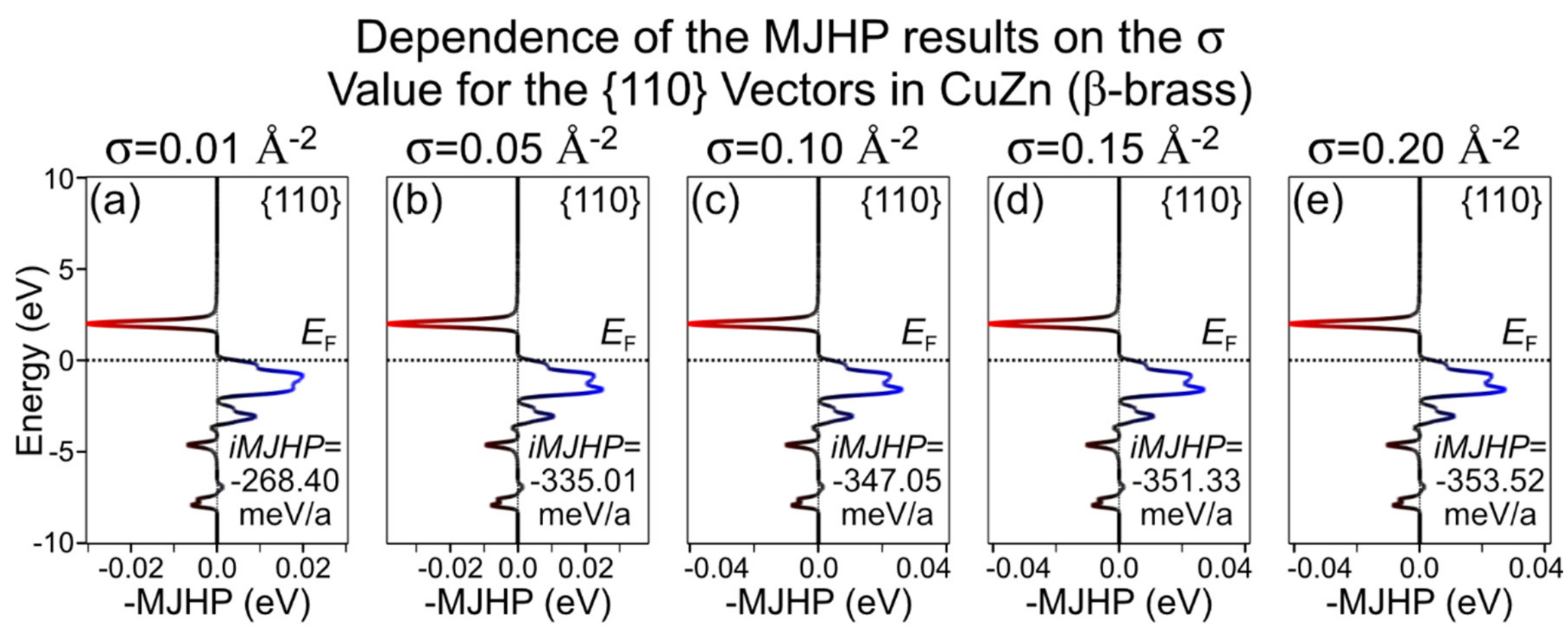

Figure S32. $MJHP_{HKL}(E)$ curves for the {110} family of reciprocal lattice vectors for β-brass, CuZn with varying σ values: (a) 0.01 Å^{-2}, (b) 0.05 Å^{-2}, (c) 0.10 Å^{-2}, (d) 0.15 Å^{-2}, and (e) 0.20 Å^{-2}.

S7. MJHP Results for Additional Structures Adopting the Archetypical Packings.

Computational details and optimized geometries for hypothetical hexagonal-close packed structures: Ga, Pd, Ag, CsCl-type structures: AlNi,[1] AuAl,[2] and PdGa,[3] and $AuCu_3$-type structures: $AuCu_3$[4] and $PdCu_3$[5].

Table S36. Computational details for the geometrical optimizations using ABINIT.

Structure	Coarse E Cutoff (Ha)	Fine E Cutoff (Ha)	*k*-point Mesh	No. *k*-points	Coarse FFT Grid	Fine FFT Grid	Total E/Cell (Ha)
Ga (hcp-Mg type)	20	24	16×16×8	150	24×24×45	27×27×48	-520.3081
Pd (hcp-Mg type)	22	32	16×16×8	150	27×27×48	30×30×60	-257.6399
Ag (hcp-Mg type)	22	32	16×16×8	150	27×27×48	30×30×60	-295.9346
AlNi (CsCl-type)	24	30	12×12×12	84	30×30×30	32×32×32	-252.5202
AuAl (CsCl-type)	18	26	12×12×12	84	30×30×30	32×32×32	-219.4080
PdGa (CsCl-type)	20	24	12×12×12	84	30×30×30	30×30×30	-389.0150
$AuCu_3$ ($AuCu_3$-type)	20	30	8×8×8	35	36×36×36	45×45×45	-731.6828
$PdCu_3$ ($AuCu_3$-type)	20	32	8×8×8	35	36×36×36	45×45×45	-722.7244

Table S37. Computational details for ABINIT calculations used for the MJHP and DOS distribution analysis.

Structure	Coarse E Cutoff (Ha)	Fine E Cutoff (Ha)	*k*-point Mesh	No. *k*-points	Coarse FFT Grid	Fine FFT Grid	Total E/Cell (Ha)
Ga (hcp-Mg type)	20	24	32×32×16	918	24×24×40	27×27×45	-520.3081
Pd (hcp-Mg type)	22	32	32×32×16	918	24×24×40	30×30×48	-257.6400
Ag (hcp-Mg type)	22	32	32×32×16	918	24×24×40	30×30×48	-295.9347
AlNi (CsCl-type)	24	30	48×48×48	2925	27×27×27	30×30×30	-252.5203
AuAl (CsCl-type)	18	26	48×48×48	2925	24×24×24	30×30×30	-219.4081
PdGa (CsCl-type)	20	24	48×48×48	2925	24×24×24	27×27×27	-389.0151
$AuCu_3$ ($AuCu_3$-type)	20	30	32×32×32	969	30×30×30	36×36×36	-731.6829
$PdCu_3$ ($AuCu_3$-type)	20	32	32×32×32	969	30×30×30	36×36×36	-722.7245

Table S38. Optimized cell parameters from ABINIT.

Structure	*a* (Å)	*b* (Å)	*c* (Å)	α	β	γ
Ga (hcp-Mg type)	5.6415	5.6415	9.3357	90	90	120
Pd (hcp-Mg type)	5.2634	5.2634	8.7043	90	90	120
Ag (hcp-Mg type)	5.5358	5.5358	9.0754	90	90	120
AlNi (CsCl-type)	5.4755	5.4755	5.4755	90	90	90
AuAl (CsCl-type)	6.0950	6.0950	6.0950	90	90	90
PdGa (CsCl-type)	5.8916	5.8916	5.8916	90	90	90
$AuCu_3$ ($AuCu_3$-type)	7.1451	7.1451	7.1451	90	90	90
$PdCu_3$ ($AuCu_3$-type)	7.0276	7.0276	7.0276	90	90	90

Table S39. Fractional atomic coordinates for the hypothetical hexagonal close-packed (hcp-Mg type) elemental structures: Ga, Pd, and Ag.

Wyckoff	Site	*x*	*y*	*z*
2*c*	1	1/3	2/3	0.25
	2	2/3	1/3	0.75

Table S40. Fractional atomic coordinates for AlNi (CsCl-type).

Wyckoff	Site	*x*	*y*	*z*
1*a*	Ni1	0	0	0
1*b*	Al1	0.5	0.5	0.5

Table S41. Fractional atomic coordinates for AuAl (CsCl-type).

Wyckoff	Site	*x*	*y*	*z*
1*a*	Al1	0	0	0
1*b*	Au1	0.5	0.5	0.5

Table S42. Fractional atomic coordinates for PdGa (CsCl-type).

Wyckoff	Site	*x*	*y*	*z*
1*a*	Ga1	0	0	0
1*b*	Pd1	0.5	0.5	0.5

Table S43. Fractional atomic coordinates for $AuCu_3$ ($AuCu_3$-type).

Wyckoff	Site	*x*	*y*	*z*
1*a*	Au1	0	0	0
3*c*	Cu1	0	0.5	0.5
	Cu2	0.5	0	0.5
	Cu3	0.5	0.5	0

Table S44. Fractional atomic coordinates for $PdCu_3$ ($AuCu_3$-type).

Wyckoff	Site	*x*	*y*	*z*
1*a*	Pd1	0	0	0
3*c*	Cu1	0	0.5	0.5
	Cu2	0.5	0	0.5
	Cu3	0.5	0.5	0

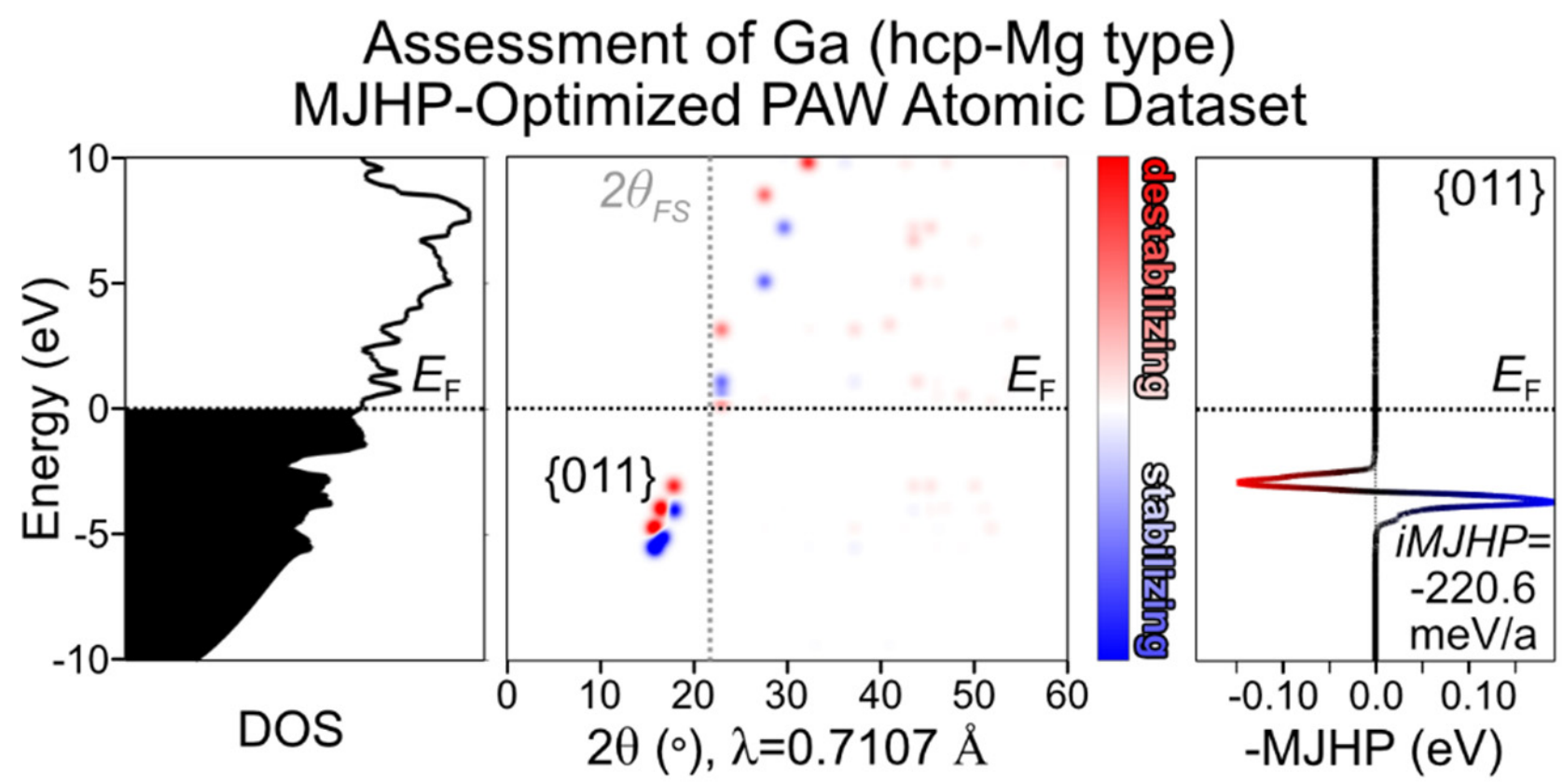

Figure S33. The DOS distribution, $MJHP(E,2\theta)$ plot, and $MJHP_{HKL}(E)$ curve for the {110} family of reciprocal lattice vectors in Ga (hcp-Mg type) using the PAW method with the MJHP optimized atomic dataset.

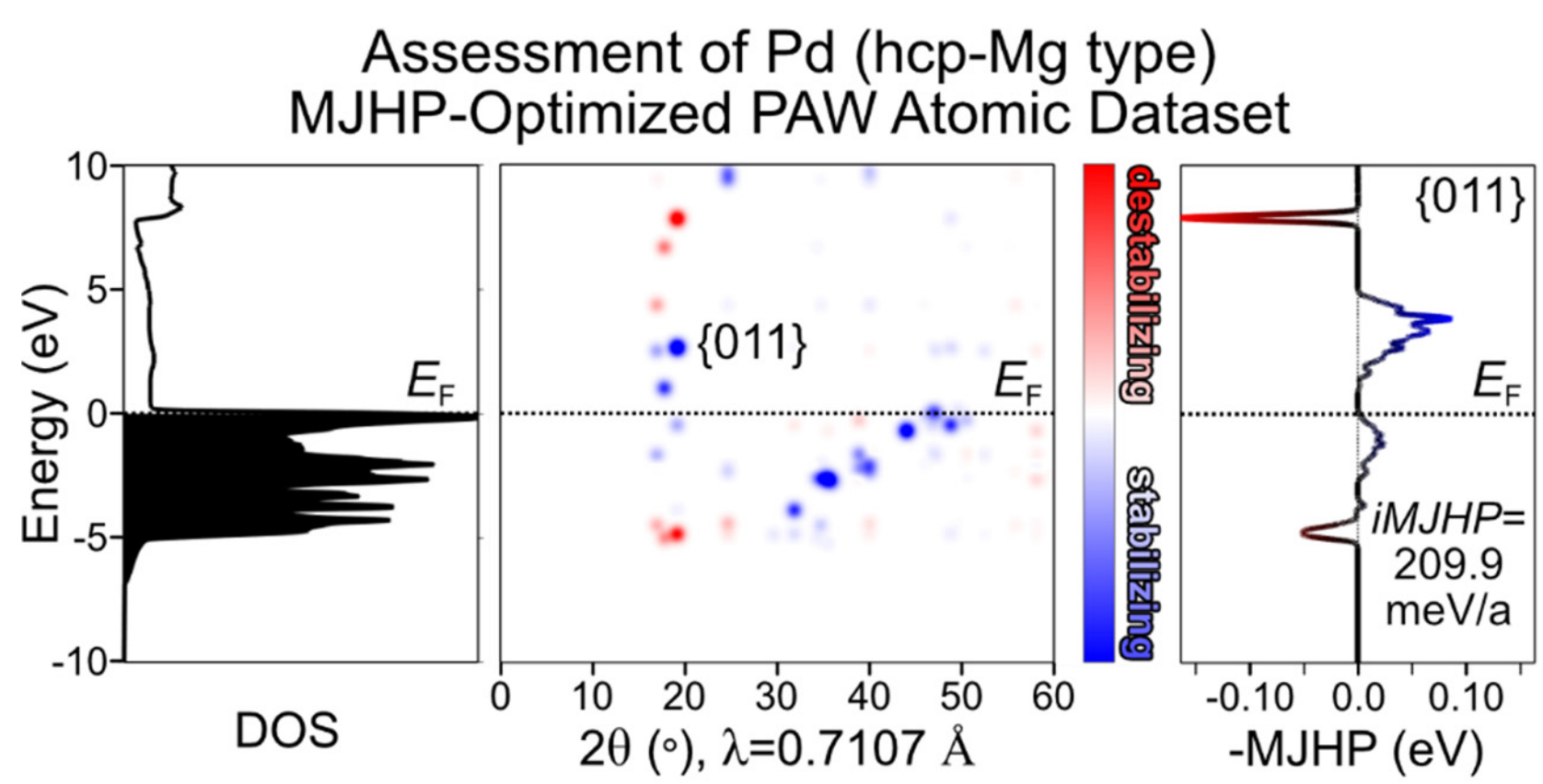

Figure S34. The DOS distribution, $MJHP(E,2\theta)$ plot, and $MJHP_{HKL}(E)$ curve for the {110} family of reciprocal lattice vectors in Pd (hcp-Mg type) using the PAW method with the MJHP optimized atomic dataset.

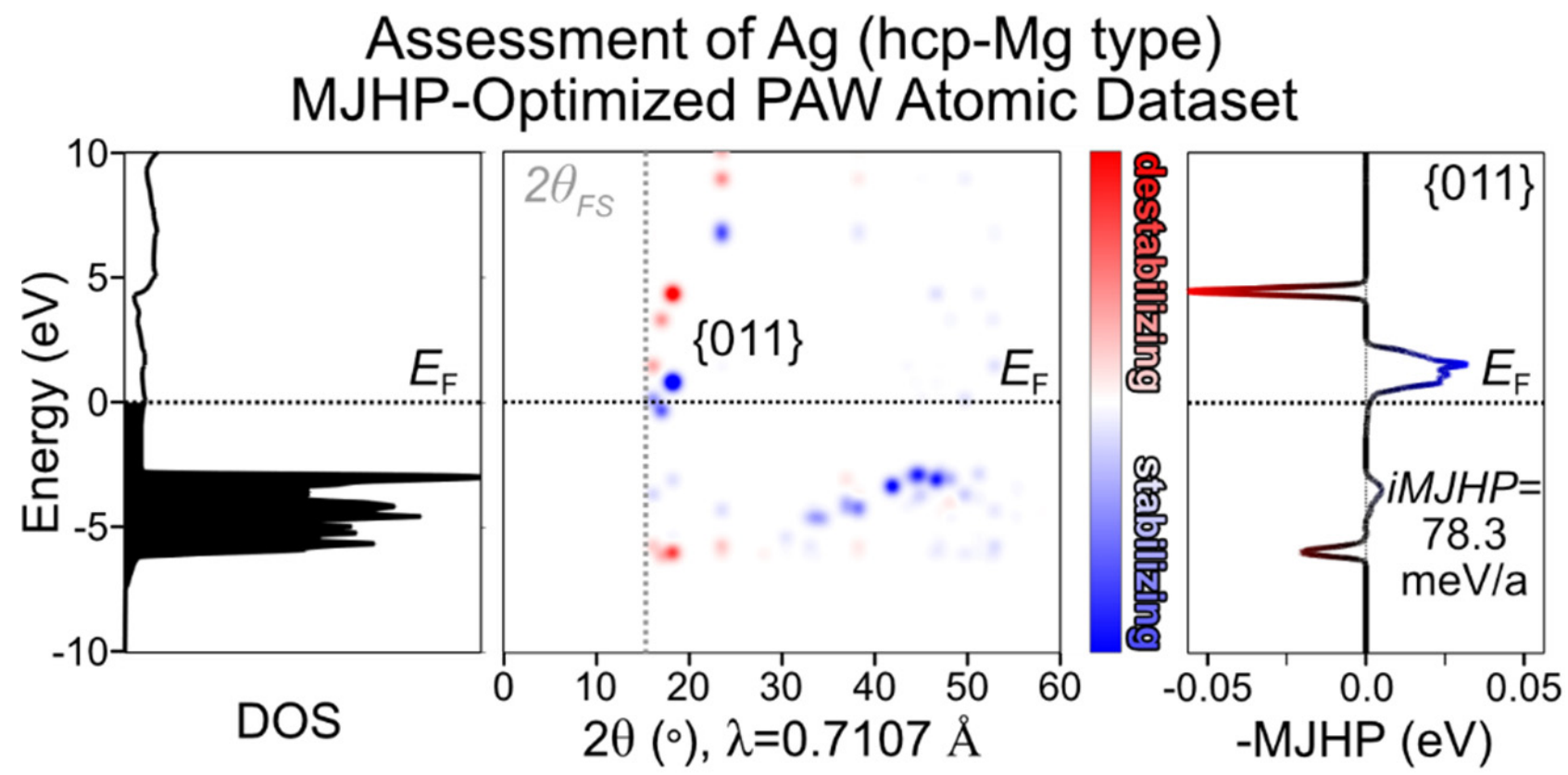

Figure S35. The DOS distribution, $MJHP(E, 2\theta)$ plot, and $MJHP_{HKL}(E)$ curve for the $\{110\}$ family of reciprocal lattice vectors in Ag (hcp-Mg type) using the PAW method with the MJHP optimized atomic dataset.

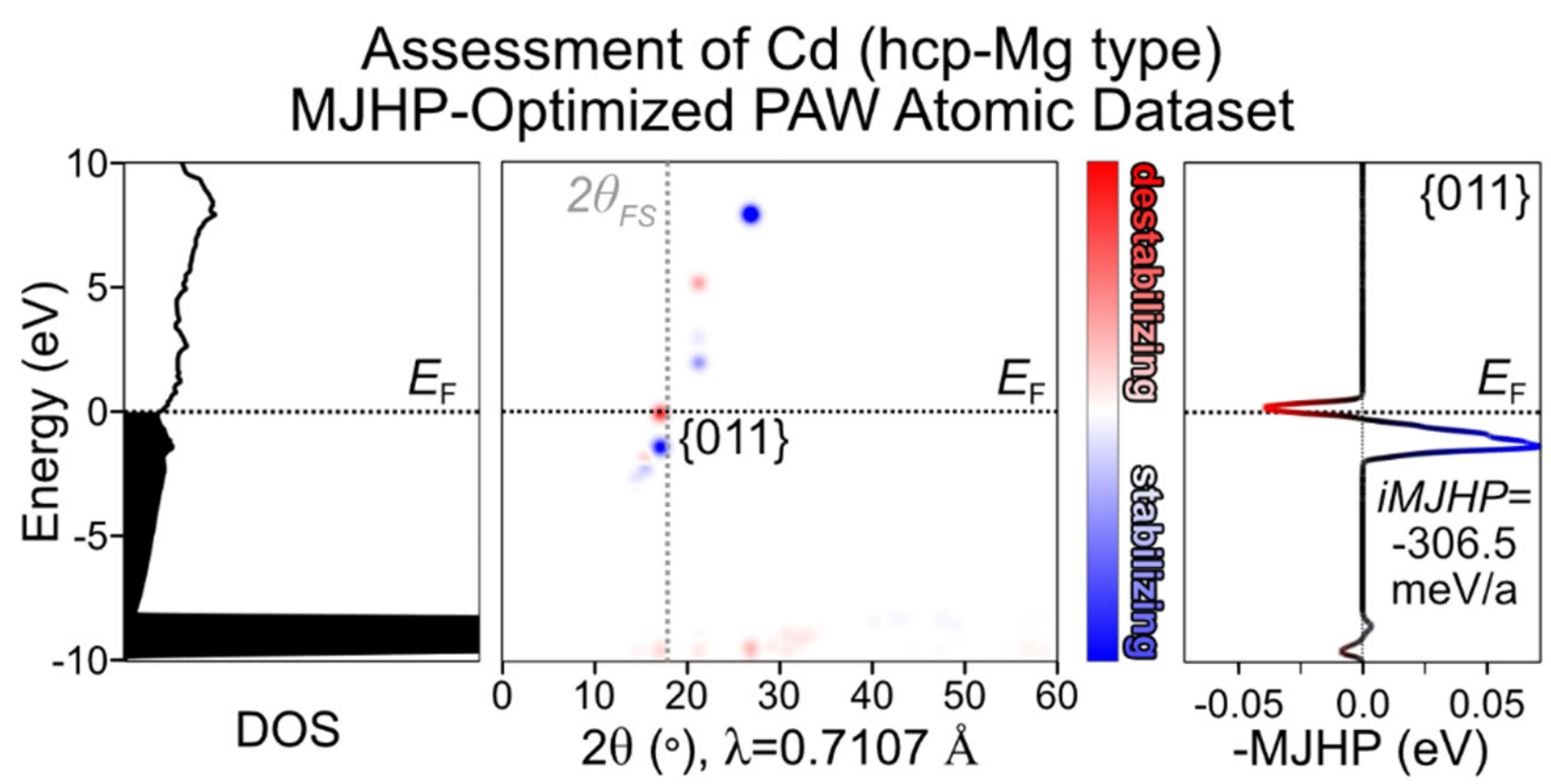

Figure S36. The DOS distribution, $MJHP(E, 2\theta)$ plot, and $MJHP_{HKL}(E)$ curve for the $\{110\}$ family of reciprocal lattice vectors in Cd (hcp-Mg type) using the PAW method with the MJHP optimized atomic dataset.

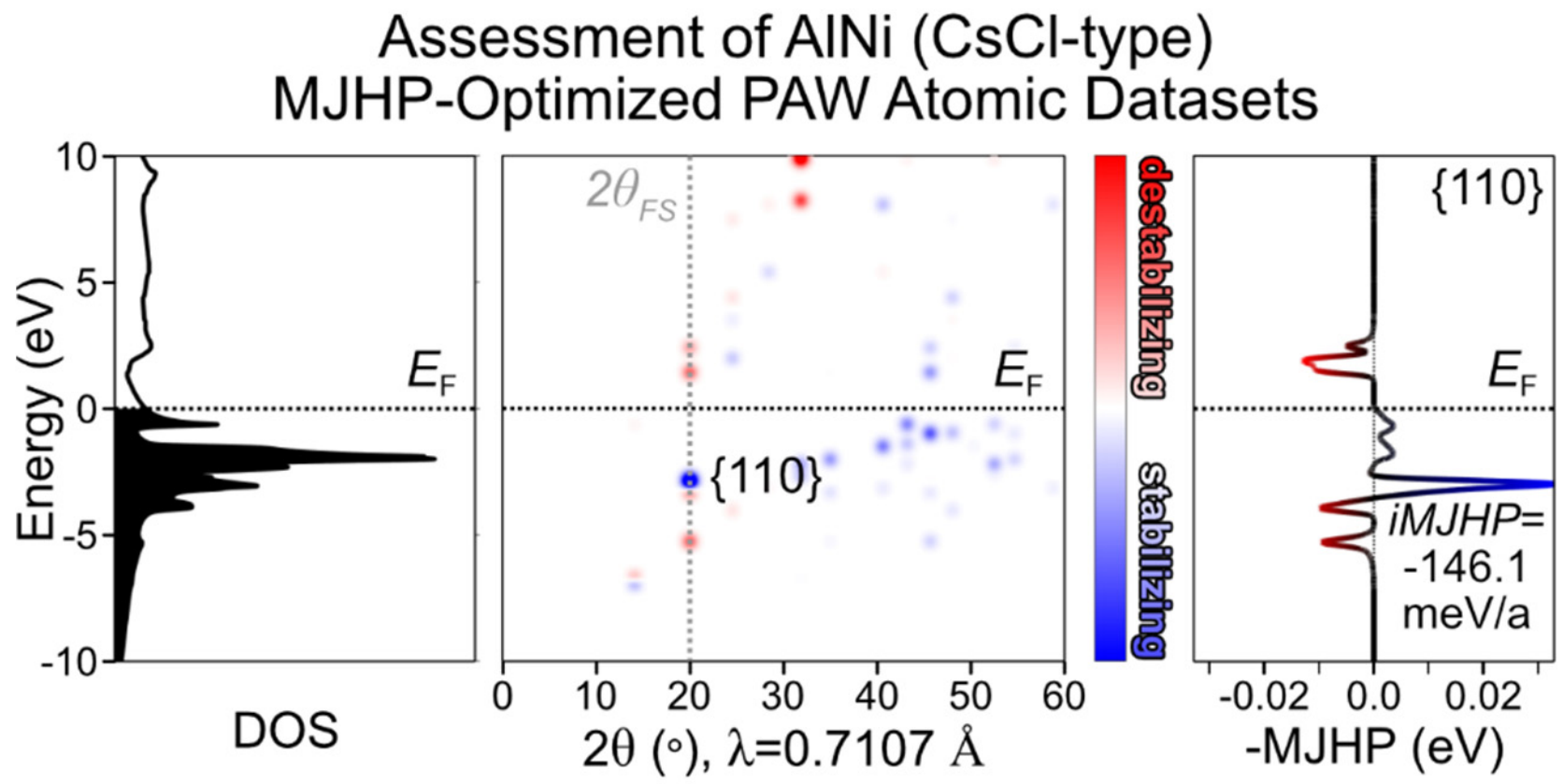

Figure S37. DOS distribution, $MJHP(E, 2\theta)$ plot, and $MJHP_{HKL}(E)$ curve for the {110} family of reciprocal lattice vectors in AlNi (CsCl-type) using the PAW method with the MJHP optimized atomic dataset.

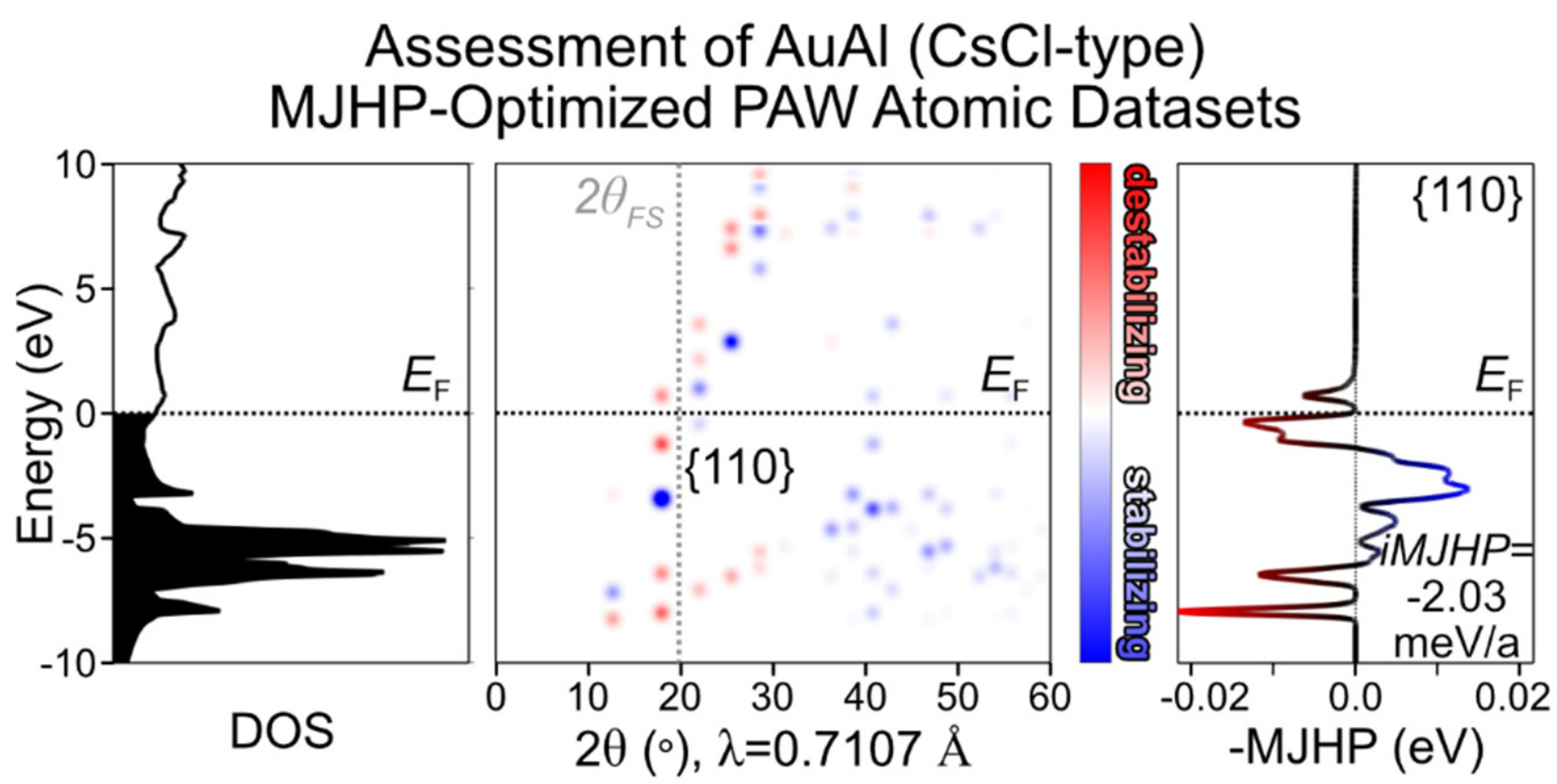

Figure S38. DOS distribution, $MJHP(E, 2\theta)$ plot, and $MJHP_{HKL}(E)$ curve for the {110} family of reciprocal lattice vectors in AuAl (CsCl-type) using the PAW method with the MJHP optimized atomic dataset.

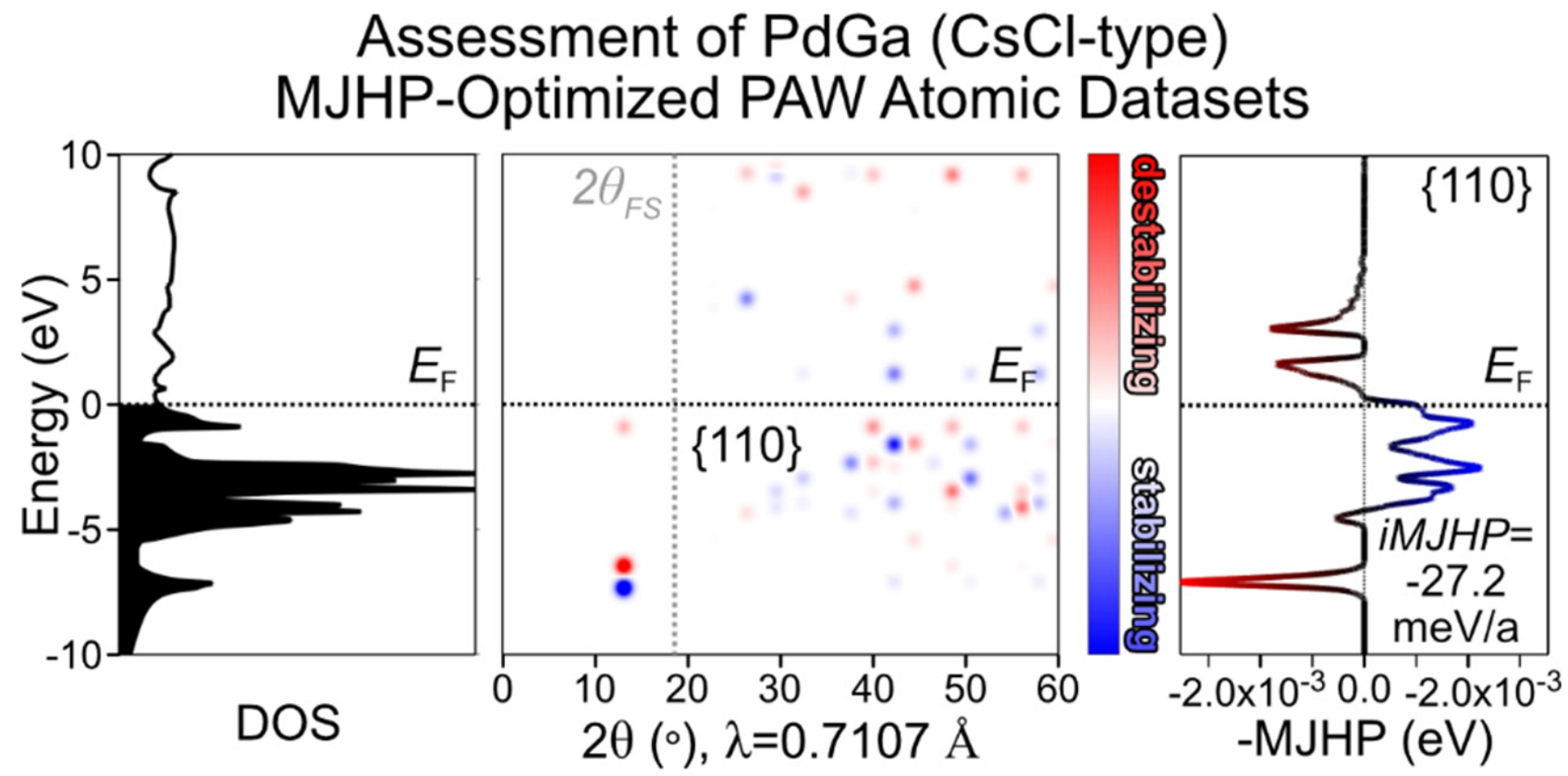

Figure S39. DOS distribution, $MJHP(E, 2\theta)$ plot, and $MJHP_{HKL}(E)$ curve for the {110} family of reciprocal lattice vectors in PdGa (CsCl-type) using the PAW method with the MJHP optimized atomic dataset.

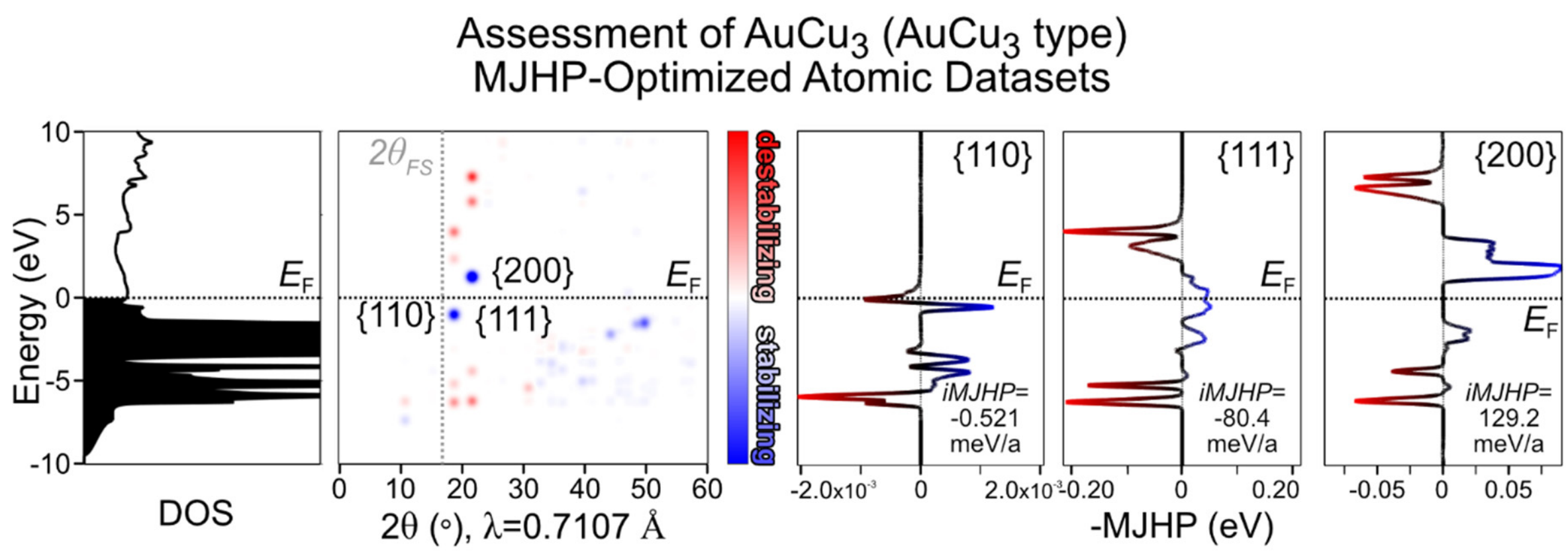

Figure S40. DOS distribution, $MJHP(E, 2\theta)$ plot, and $MJHP_{HKL}(E)$ curves for the {110}, {111}, and {200} families of reciprocal lattice vectors in $AuCu_3$ ($AuCu_3$-type) using the PAW method with the MJHP optimized atomic dataset.

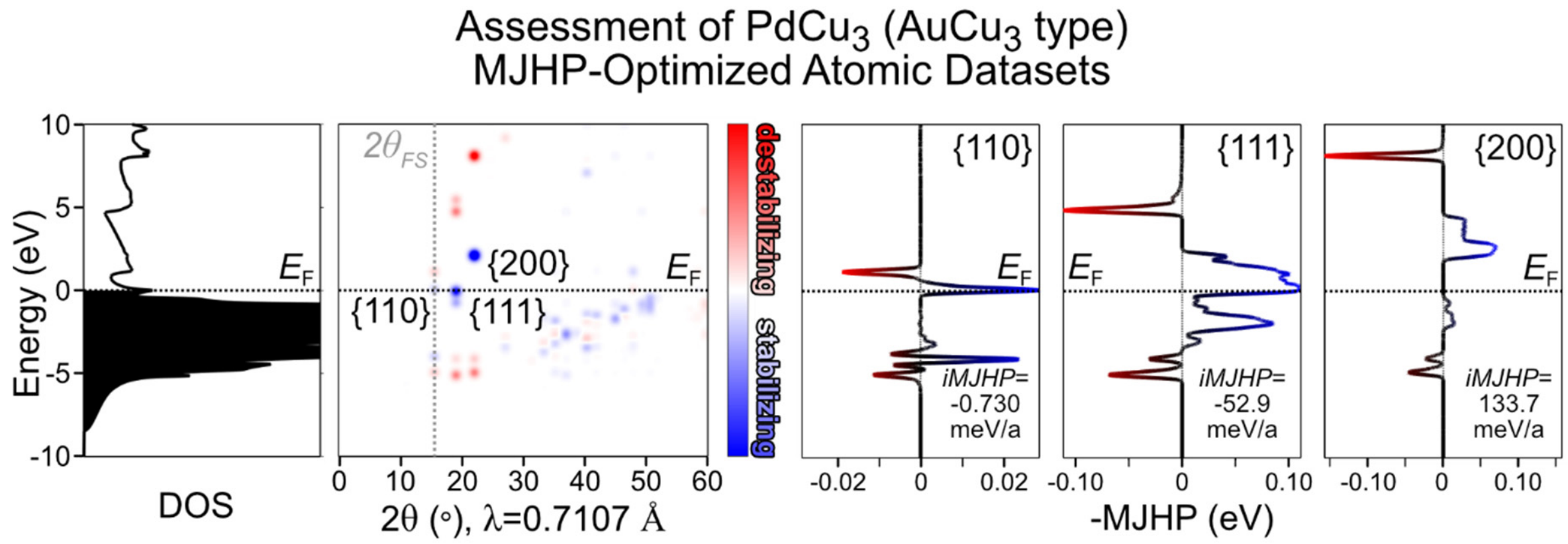

Figure S41. DOS distribution, $MJHP(E, 2\theta)$ plot, and $MJHP_{HKL}(E)$ curves for the {110}, {111}, and {200} families of reciprocal lattice vectors in $PdCu_3$ ($AuCu_3$-type) using the PAW method with the MJHP optimized atomic dataset.

REFERENCES

(1) Becker, K.; Ebert, F. Röntgenspektroskopie an Metallverbindungen. *Z. Phys.* **1923**, *16,* 165-169.
(2) Abramyan, G. K.; Pilyankevich, A. N.; Frantsevich, I. N. Structure of the Compound AuAl in the Form of a Thin Film. *Sov. Phys. Dokl.* **1971**, *16,* 138.
(3) Bhan, S.; Kudielka, H. Ordered bcc-Phases at High Temperatures in Alloys of Transition Metals and B-Subgroup Elements. *Int. J. Mater. Res.* **1978**, *69,* 333-336.
(4) Blanc, M.; Richter, K.; Schiebold, E. Eine Prüfung der Tammannschen Theorie der Resistenzgrenzen am System Gold–Kupfer. Aufstellung neuer Gesichtspunkte. *Ann. Phys.* **1928**, *391,* 929-1005.
(5) Jones, D. M.; Owen, E. A. Experimental Study of the Variation of the Degree of Order with Temperature in a Copper-Palladium Alloy. *Proc. Phys. Soc. B* **1954**, *67,* 297.
